# Supplementary material for: Prevalence and impact of combined vision and hearing (dual sensory) impairment: A scoping review
Source: PLOS Glob Public Health. 2023 May 16;3(5):e0001905. doi: 10.1371/journal.pgph.0001905 (PMC10187940; doi:10.1371/journal.pgph.0001905)
Supplement: S1 Checklist — (DOCX) [file pgph.0001905.s001.docx]

**Preferred Reporting Items for Systematic reviews and Meta-Analyses extension for Scoping Reviews (PRISMA-ScR) Checklist**

| **SECTION** | **ITEM** | **PRISMA-ScR CHECKLIST ITEM** | **REPORTED ON PAGE #** |
| --- | --- | --- | --- |
| **TITLE** | | | |
| Title | 1 | Identify the report as a scoping review. | 1 |
| **ABSTRACT** | | | |
| Structured summary | 2 | Provide a structured summary that includes (as applicable): background, objectives, eligibility criteria, sources of evidence, charting methods, results, and conclusions that relate to the review questions and objectives. | 2 |
| **INTRODUCTION** | | | |
| Rationale | 3 | Describe the rationale for the review in the context of what is already known. Explain why the review questions/objectives lend themselves to a scoping review approach. | 3 |
| Objectives | 4 | Provide an explicit statement of the questions and objectives being addressed with reference to their key elements (e.g., population or participants, concepts, and context) or other relevant key elements used to conceptualize the review questions and/or objectives. | 3 |
| **METHODS** | | | |
| Protocol and registration | 5 | Indicate whether a review protocol exists; state if and where it can be accessed (e.g., a Web address); and if available, provide registration information, including the registration number. | 3 |
| Eligibility criteria | 6 | Specify characteristics of the sources of evidence used as eligibility criteria (e.g., years considered, language, and publication status), and provide a rationale. | 4 |
| Information sources* | 7 | Describe all information sources in the search (e.g., databases with dates of coverage and contact with authors to identify additional sources), as well as the date the most recent search was executed. | 4 |
| Search | 8 | Present the full electronic search strategy for at least 1 database, including any limits used, such that it could be repeated. | Supplementary  material |
| Selection of sources of evidence† | 9 | State the process for selecting sources of evidence (i.e., screening and eligibility) included in the scoping review. | 4 |
| Data charting process‡ | 10 | Describe the methods of charting data from the included sources of evidence (e.g., calibrated forms or forms that have been tested by the team before their use, and whether data charting was done independently or in duplicate) and any processes for obtaining and confirming data from investigators. | 5 |
| Data items | 11 | List and define all variables for which data were sought and any assumptions and simplifications made. | 5 |
| Critical appraisal of individual sources of evidence§ | 12 | If done, provide a rationale for conducting a critical appraisal of included sources of evidence; describe the methods used and how this information was used in any data synthesis (if appropriate). | N/A |
| Synthesis of results | 13 | Describe the methods of handling and summarizing the data that were charted. | 5 |
| **RESULTS** | | | |
| Selection of sources of evidence | 14 | Give numbers of sources of evidence screened, assessed for eligibility, and included in the review, with reasons for exclusions at each stage, ideally using a flow diagram. | 6 |
| Characteristics of sources of evidence | 15 | For each source of evidence, present characteristics for which data were charted and provide the citations. | Supplementary material |
| Critical appraisal within sources of evidence | 16 | If done, present data on critical appraisal of included sources of evidence (see item 12). | N/A |
| Results of individual sources of evidence | 17 | For each included source of evidence, present the relevant data that were charted that relate to the review questions and objectives. | Supplementary material |
| Synthesis of results | 18 | Summarize and/or present the charting results as they relate to the review questions and objectives. | 6-17 |
| **DISCUSSION** | | | |
| Summary of evidence | 19 | Summarize the main results (including an overview of concepts, themes, and types of evidence available), link to the review questions and objectives, and consider the relevance to key groups. | 17 |
| Limitations | 20 | Discuss the limitations of the scoping review process. | 19 |
| Conclusions | 21 | Provide a general interpretation of the results with respect to the review questions and objectives, as well as potential implications and/or next steps. | 19 |
| **FUNDING** | | | |
| Funding | 22 | Describe sources of funding for the included sources of evidence, as well as sources of funding for the scoping review. Describe the role of the funders of the scoping review. | 25 |

**S2 Text: Search strategy**

**Search strategy (Medline)**

1     exp hearing loss/ (68101)

2     Persons With Hearing Impairments/ (2667)

3     Hearing Disorders/ (14506)

4     (hearing adj3 (loss or impair$)).tw. (54240)

5     (deaf or deafness).tw. (29117)

6     (hard adj2 hearing).tw. (1373)

7     or/1-6 (107053)

8     exp Blindness/ (24168)

9     Vision, Low/ (3326)

10     Visually Impaired Persons/ (2391)

11     ((low$ or impair$ or partial$ or loss$) adj2 (vision or visual$ or sight$)).tw. (48082)

12     blindness.tw. (27159)

13     blind.tw. (187642)

14     (double adj1 blind$).tw. (148626)

15     double-blind$.tw. (148540)

16     (triple adj1 blind$).tw. (938)

17     triple-blind$.tw. (874)

18     or/14-17 (149490)

19     13 not 18 (49156)

20     8 or 9 or 10 or 11 or 12 or 19 (125937)

21     7 and 20 (4724)

22     (dual sensory adj2 (impair$ or loss)).tw. (159)

23     (deaf adj1 blind).tw. (263)

24     deafblind.tw. (54)

25     (deafness adj3 blindness).tw. (484)

26     (low adj2 vision adj5 (deaf$ or hearing)).tw. (21)

27     (visual adj2 impair$ adj5 (deaf$ or hearing)).tw. (450)

28     (vision adj2 loss adj5 (deaf$ or hearing)).tw. (245)

29     (sight adj2 loss adj5 (deaf$ or hearing)).tw. (16)

30     (sight adj2 impair$ adj5 (deaf$ or hearing)).tw. (13)

31     (hearing adj2 loss adj5 (blind$ or vision)).tw. (439)

32     (hearing adj2 impair$ adj5 (blind$ or vision)).tw. (366)

33     or/22-32 (2155)

34     21 or 33 (5031)

35     Prevalence/ (283471)

36     Epidemiology/ (12307)

37     Epidemiological Monitoring/ (6990)

38     prevalence.tw. (602128)

39     impact$.tw. (1048808)

40     exp Mortality/ (373797)

41     Death Certificates/ (5237)

42     Life Expectancy/ (17194)

43     years lived.tw. (905)

44     mortality.tw. (728960)

45     death$.tw. (788627)

46     survival.tw. (911110)

47     (fatality or fatalities).tw. (26740)

48     exp Activities of Daily Living/ (99758)

49     ((everyday or function$) adj2 living).tw. (2813)

50     Social Support/ (69137)

51     Social isolation/ (13010)

52     (participat$ or autonomy or independence or independent).tw. (1387187)

53     Interpersonal Relations/ (70704)

54     Communication Disorders/ (2220)

55     (social$ adj3 (factor$ or interact$ or isolat$ or network$ or support$ or participat$ or relationship$)).tw. (116790)

56     (communicat$ or relationship$).tw. (1523571)

57     Vulnerable Populations/ (10037)

58     (vulnerab$ or maltreatment or harm or abuse or protect$ or safeguard$).tw. (1044893)

59     adult protection.tw. (38)

60     at risk.tw. (168471)

61     "Quality of Life"/ (188486)

62     Value of Life/ (5685)

63     Quality Adjusted Life Year/ (11816)

64     (quality adj2 life).tw. (269976)

65     (QUALY$ or DALY$ or HRQOL).tw. (18549)

66     (sf36 or sf 36 or short form 36 or shortform 36).tw. (24982)

67     life satisfaction.tw. (7329)

68     (well adj1 being).tw. (73751)

69     (self adj2 rate$ adj2 health$).tw. (7593)

70     Accidental Falls/ (23542)

71     Risk/ (120960)

72     (fall$ or accident$).tw. (305836)

73     Cognitive Dysfunction/ (15306)

74     Dementia/ (49736)

75     dementia.tw. (103349)

76     (cognitive adj2 (function$ or impair$)).tw. (114982)

77     (cognitive adj2 (declin$ or dysfunction$)).tw. (36560)

78     Mental Health/ (36585)

79     Anxiety Disorders/ (32993)

80     (anxiety or anxious).tw. (188314)

81     Depressive Disorder/ (71978)

82     (depression or depressed).tw. (378183)

83     mental health.tw. (135687)

84     or/35-83 (7581870)

85     34 and 84 (2230)

86     exp case reports/ (2079015)

87     (case adj2 report$).tw. (482133)

88     86 or 87 (2178800)

89     85 not 88 (2024)

90     limit 89 to (address or autobiography or bibliography or biography or clinical trial, veterinary or clinical trials, veterinary as topic or comment or editorial or "expression of concern" or festschrift or interactive tutorial or interview or letter or news or observational study, veterinary or personal narrative or portrait or randomized controlled trial, veterinary or video-audio media or webcast) (29)

91     89 not 90 (1995)

**S1 Table:** Studies reporting prevalence of dual sensory impairment (DSI) by age group

| First author; year of publication | Study design; setting | Sample size | Definition of DSI | Estimated prevalence of DSI, % (95% CI if reported) |
| --- | --- | --- | --- | --- |
| All ages | | | | |
| **Population-based** | | | | |
| Khandekar et al. (2004); Oman | CS | 11,400 | V: <6/120 better eye H: 3FA (500,1000,2000) ≥26dB better ear | 0.25 (0.15, 0.34); Partial double disability: 0.35 (0.24, 0.47) |
| Mactaggart et al. (2017); India& | CS | 3,574 | V: <6/12 better eye H: ≥41 dB adults; ≥35 children PTA better ear | 1.91 (1.52, 2.38) |
| **Rehabilitation centre or register-based** | | | | |
| Dammeyer et al. (2010); Denmark | CS; register | 190 | V: <6/60 eye not specified H: 3FA (500,1000,2000) ≥80dB; ear not specified | 0.003  1:15 000 children, 1:34 000 adults |
| Wittich et al. (2012); Canada | Case series report; rehab centre | 564 | V: <6/18 better eye H: ≥25dB PTA better ear | 0.01 (0.01, 0.16) |
| Children | | | | |
| Linden-Bostrom et al. (2015); Sweden | CS; school | 7,793 | V/H: self-report single binary question | 0.30 (0.18, 0.45) |
| Only adults (≥18 years) | | | | |
| **Population-based** | | | | |
| Armstrong et al. (2016); USA | CS | 2006: 2,591 2010: 3,956 | V/H: self-report single binary question | 2006: 2.70 (2.11,3.40);  2010: 2.96 (3.45, 3.53) |
| Armstrong et al. (2021); USA | CS | ARIC: 936  BSLA: 420 | V/H: self-report single binary question | ARIC: 44.3 (41.2,47.5)  BSLA: 17.4 (13.90, 21.40) |
| Caban et al. (2005); USA** | CS | 195,801 | V: self-report single categorical question H: self-report single binary question | 3.3 (3.20, 3.40) |
| Lam et al. (2006); USA | CS | 116,796 | V/H: self-report single binary question | 1.25 (1.18, 1.32) |
| Loprinzi et al. (2013)^^; USA | CS | 1,445 | V: ≤6/12 either eye H: >25dB PTA better ear | 2.0 (1.34, 2.87) |
| Loprinzi et al. (2013); USA^^$ | CS | 567 | V/H: self-report single question with Likert scale | 3.0 (1.80, 4.75) |
| Swenor et al. (2013); USA | CS | NS | V: <6/12 better eye  H: >25 PTA better ear | 40-49 years: 0.1; ≥80 years: 11.3 |
| Olakunde et al. (2020); USA | CS | 468,303 | V/H: self-report, single binary question | 0.30 (0.20, 0.31) |
| Pardhan et al. (2020); Spain | CS | 23,809 | V/H: self-report, single question categorical | 3.90 (3.70, 4.20) |
| Reed et al. (2020); USA | CS/RCS; | 10,748 | V/H: self-report, single question Likert | 1.13 (0.90, 1.30) |
| Khurana et al. (2021)’ England | CS | 7,546 | V: self-report, single question with Likert scale  H: self-report, single question binary | 3.72 (3.30, 4.18) |
| Dammeyer et al. (2013); Denmark | CS | 916 | not specified | 0.02 (0.00, 0.07) |
| Jung et al. (2022); Korea$ | PCS | 771,128 | V: <6/12 better eye  H: >40dB PTA at least one ear | 1.13 (1.11, 1.15) (aged ≥30 years) |
| **Clinic- or register-based** | | | | |
| Smith et al. (2008); USA | RCS; clinic | 400 | V: <6/12 better eye H: ≥40dB PTA better ear | 5.0 (3.1, 6.9) |
| Meuwese-Jongejeugd et al. (2008); The Netherlands$ | CS; other | 1,359 | V: <6/12 better eye  H: >25dB PTA better ear | 5.00 (3.90, 6.20) |
| Older adults | | | | |
| 40+ | | | | |
| **Population-based** | |  |  |  |
| Dawes et al. (2014); United Kingdom | CS | 116,682 | V: <6/7.5 in better eye H: poor performance on Digit Triple Test | 3.10 (3.30, 3.20) |
| Fischer et al. (2009); USA | PCS | 1,854 | V: <6/12 better eye H: >25dB PTA better ear | 7.2 (6.10, 8.50) |
| Miyawaki et al. (2019); Japan | RCS | 9,522 | V/H: self-report single categorical question | 0.97 (0.77, 1.19) |
| Ma et al. (2021); China### | PCS | 13,097 | V/H: self-report single question with Likert scale | 5.50 (5.10, 5.90) |
| Liu et al. (2022); China### | PCS | 13,690 | V/H: self-report multiple questions | 10.10 (9.60, 10.60) |
| Hajek et al (2022); Germany | PCS | 5,138 | V/H: self-report single question binary | 15.9 (14.9, 16.9) |
| Zhao et al. (2021); China### | CS;pop | 13,914 | V/H self-report single question categorical | 2.10 (1.90, 2.40) |
| **Clinic- or register-based** | |  |  |  |
| Ho et al. (2021); Singapore | CS; care home | 123 | V: <6/12 better eye  H: >40dB PTA better ear | 78.90 (71.60, 86.10) |
| ≥45 years | | | | |
| Fuller et al. (2018); USA | CS | 7,210,535 | V/H: self-report single binary question | 1.62 (1.61, 1.64) |
| Klein et al. (1998); USA | PCS | 3,397 | V: poor ARMD grade (using grading system) H: >25dB in better ear | 15.10 (13.91, 16.35) |
| Lee et al. (2005); USA | PCS | 60,997 | V/H: self-report single binary question | 2.35 (2.23, 2.47)  Higher prevalence in M |
| Mick et al. (2018); Canada* | CS | 21,241 | V/H: self-report single question with Likert scale | 1.90 (1.60, 2.10) |
| Mick et al. (2021); Canada* | CS | 29,007 | V: <6/9.5 better eye H: >25dB PTA better ear | 9.56 (9.22, 9.90) |
| Sun et al. (2020); China### | PCS | 37,076 | V/H: clinical measurement, but not VA or audiometry (clinician judgment) | 20.96 (20.60, 21.40) |
| Rong et al. (2020); China### | CS | 10,575 | V: self-report multiple questions  H: self-report single question with Likert scale | 58.60 (57.90, 59.30) |
| ≥50 years | | | | |
| **Population-based** | | | | |
| Kiely et al. (2016); Australia# | PCS | 4,160 | V: <6/12 eye not specified H: >25dB PTA better ear | 8.15 (7.33, 9.02) |
| Liljas et al. (2018); England | PCS | 4,621 | V/H: self-report single question with Likert scale | 3.87 (3.33, 4.47) |
| Maharani et al. (2020); USA | CS | 19,618 | V/H: self-report single question with Likert scale | 5.02 (4.71, 5.33) |
| Mitoku et al. (2016); Japan | PCS | 1,754 | V: VA chart definition not clear H: self-report single categorical question | 18.24 (16.46, 20.13) |
| Maharani et al. (2018); Multiple countries | CS | 45,805 | V/H: self-report single question with Likert scale | England: 5.89 (5.46, 6.33)  USA: 7.10 (6.67, 7.76) |
| Viljanen et al. (2014); Multiple European countries | CS | 27,536 | V/H: self-report single categorical question | Pooled: 5.9   - Belgium: 5.40 (4.70, 6.19) - Sweden: 1.70 (1.27, 2.23) - France: 6.90 (6.04, 7.87) - Italy: 10.30 (99.13, 11.54) - Austria: 4.00 (3.16, 5.00) - Denmark: 4.80 (3.84, 5.99) - Germany: 4.80 (4.05, 5.63) - Greece: 5.20 (4.38, 6.10) - Switzerland: 1.60 (0.90, 2.56) - Spain: 9.40 (8.24, 10.64) - The Netherlands: 4.00 (3.31, 4.78) |
| Graue-Hernández et al. (2019); Mexico | CS | 1,511 | V: <6/18 better eye H: self-report multiple question | 4.37 (3.39, 5.52) |
| Beall et al. (1986); Nepal | CS | 117 | V: VA ≤6/60 better eye H: ≥60dB; frequencies/average NS; ear NS | Overall prevalence not presented; 50-60 year-old men: 2.1;  70 year-old men: 29.0 |
| Bright et al. (2020); Multiple& | CS | Cameroon: 3,567  India: 3,573 | V: <6/18 better eye H: ≥41dB PTA in better ear | India: 4.40 (3.00, 6.40)  Cameroon: 4.80 (3.00, 7.70) |
| Shakarchi et al. (2020); USA | CS;pop | 13,092 | V/H: self-report single question with Likert scale | 8.10 (7.64, 8.58) |
| Ogliari et al. (2021); Multiple | PCS | 50,986 | V/H: self-report single question with Likert scale | 7.60 (7.30, 7.80) |
| **Clinic, care home or register based** | | | | |
| Mudie et al. (2018); USA$ | CS; clinic | 220 | V: mean deviation on visual field testing worse than -5dB better eye H: >25dB better ear | 19.53 (14.46, 25.47) |
| Vreeken et al. (2014); Multiple European countries | CS; vision rehab centre | 1,396 | V/H: self-report multiple question | Pooled: 44.8 (42.2,47.5)   - Belgium: 32.71 (27.14, 38.67) - The Netherlands: 47.83 (44.82, 50.85) |
| Yamada et al. (2014); Multiple European countries | CS; care home | 4,007 | V/H: self-report single categorical question | Pooled: 31.8   - Czech Republic: 39.76 (35.41, 44.23) - England: 14.20 (11.30, 17.57) - Finland: 21.27 (17.54, 25.38) - France: 34.92 (30.67, 39.35) - Germany:33.20 (29.06, 37.54) - Israel: 32.83 (28.83, 37.02) - Italy: 49.90 (45.52, 54.29) - The Netherlands: 27.19 (23.50, 31.12) |
| Lach et al. (2019); NS | CS; care home | 225 | V: <6/15; eye not specified H: ≥40dB PTA better ear | 29.78 (23.88, 36.21) |
| ≥55 years | | | | |
| **Population-based** | | | | |
| Chia et al. (2006); Australia#  Schneider et al.# (2012); Australia | PCS | 2,015 | V: <6/12 better eye H: >25dB PTA better ear | Presenting VA: 6.04 (5.04, 7.23)  Corrected VA; 2.1 corrected VA  Prevalence higher in women |
| Gopinath et al. (2014); Australia# | PCS | 2,443 | V: <6/12 better eye H: >25dB PTA better ear | 3.89 (3.16, 4.73) |
| Gopinath et al. (2021); Australia# | PCS | 1,085 | V: <6/12 better eye H: >25dB PTA better ear | 13.50 (11.57, 15.73) |
| Capella-McDonnall et al. (2005); USA** | CS | 9,832 | V: self-report single binary question H: self-report single categorical question | 7.34 (6.69, 8.02) |
| Reuben et al. (1999); USA | PCS | 5,677 | V: <6/12 better eye H: >40dB at 1 or/and 2kHz in better ear  (also measured V/H: self-report single question binary) | Clinical measurement: 4.60 (3.24, 6.31) Self-report: 4.09 (3.17, 5.20) |
| Raina et al. (2004); Canada | CS | 16,613 | V/H: self-report single binary question | 3.00 (2.74, 3.27) |
| **Clinic-based** | | | | |
| Keller et al. (1999); USA | CS; clinic | 576 | Vision: ≤20/70 in better eye (near vision) Hearing: fail whisper voice test | 13.02 (10.38, 16.05) |
| ≥60 years | | | | |
| **Population-based** | | | | |
| Kulmala et al. (2009); Finland$ | PCS | 428 | V: <6/60 eye not specified H: ≥21 dB PTA better ear | 17.29 (13.83, 21.21) |
| Mueller-Schotte et al. (2018); Netherlands | PCS | 9,319 | V/H: self-report single binary question | 18.17 (17.39, 18.97) |
| Chou et al. (2004); Hong Kong | CS | 2,003 | V/H: self-report single categorical question | 6.50 (5.45, 7.66) |
| Kwon et al. (2015); South Korea^^^ | CS | 5,260 | V: <6/18 better eye H: >40dB PTA better ear | 5.10 (4.52, 5.72) |
| Tareque et al. (2019); Singapore | PCS | 3,452 | V/H: self-report single question with Likert scale | 11.62 (10.57, 12.73) |
| Heine et al. (2019); China### | CS | 8,268 | V/H: self-report single question with Likert scale | 57.20 (56.12, 58.27) |
| Deepthi et al. (2012); India | CS | 257 | V: <6/18 better eye H: >25dB PTA better ear | 37.14 (29.97, 44.76) |
| Harithasan et al. (2020); Malaysia | CS | 229 | V: <6/12 better eye H: ≥26 PTA better ear | 8.30 (5.07, 12.65) |
| Byeon et al. (2021); Korea | PCS | 6,520 | V/H: self-report single question with Likert scale | 40.40 (39.20, 41.60) |
| Gadkaree et al. (2016); USA | CS | 276,233 | V/H self-report single question with Likert scale | 9.87 (6.36, 14.44) |
| Ramamurthy et al. (2014); India | CS | 175 | V: <6/18 better eye  H: 3FA >40dB better ear | 17.71 (12.80, 24.00) |
| Phua et al. (2022); Singapore | CS | 4,077 | V/H: self-report single question with Likert scale | 12.80 (11.80, 13.90) |
| **Clinic or other based** | | | | |
| Hickson et al. (1999); Australia | PCS; community centres, senior citizens organisations, local newspapers, TV | 240 | V: <6/9 eye not specified H: >25dB PTA better ear | 18.75 (14.02, 24.27) |
| Cosh et al. (2018); Norway | PCS; clinic | 2,890 | V: <6/9 eye not specified H: self-report single question binary | 6.77 (5.57, 7.92) |
| Marmamula et al. (2021); India | CS; CH | 867 | V: <6/18 better eye  H: self-report multiple questions | 5.80 (4.30, 7.50) |
| Kwan et al. (2022); China | PCS; CH | 2,233 | V/H: not specified/unclear | 12.20 (10.90, 13.70) |
| ≥65 years | | | | |
| **Population-based** | | | | |
| Heine et al. (2019); Australia | RCS | 1,000 | V/H: self-report single categorical question | 11.00 (9.13, 13.11) |
| Kiely et al. (2013); Australia*** | PCS | 1,611 | V: <6/12 better eye H: >25dB PTA better ear | 21.11 (18.87, 23.48) |
| Liu et al. (2015); USA | PCS | 3,871 | V: self-report single binary question H: self-report single categorical question | 4.73 (4.08, 5.44) |
| Simning et al. (2018); USA | PCS | 7,507 | V/H: self-report single binary question | 1.30 (1.04, 1.56) |
| Fisher et al. (2014); Canada | PCS | 4,926 | V: <6/15 better eye H: ≥35dB PTA better ear | 6.96 (6.27, 7.71) |
| Schneck et al. (2012); USA | CS | 446 | V: <6/18 either eye H: >40dB PTA better ear | 3.10 (1.73, 5.21) |
| Campbell et al. (1999); USA** | CS | 8,767 | V/H: self-report multiple question | 8.6 (7.9, 9.3) |
| Deardorff et al. (2019); USA | PCS | 24,009 | V/H: self-report single categorical question | 18.22 (17.74, 18.72) |
| Forbes et al. (1991); Canada | CS | 132,337 | V/H: self-report question type not clear | 4.80 (4.77, 4.83) |
| Grue et al. (2008); Norway | CS | 332 | V: <6/7.5 better eye H: ≥30dB PTA better ear | 30.1 |
| Kim et al. (2015); South Korea^^^ | CS | 3,636 | V: <6/18 either eye H: >40dB PTA better ear | 6.16 (5.40, 6.99) |
| Chou et al. (2008); England** | PCS | 3,782 | V/H: self-report single question with Likert scale | 7.10 (6.31, 7.96) |
| Lyu et al. (2018); South Korea | CS | 3,831 | V/H: self-report single question with Likert scale | 5.85 (5.12, 6.63) |
| Kuo et al (2021); USA | PCS | 7,562 | V/H: self-report multiple questions | 3.10 (2.70, 3.50) |
| Assi et al. (2021); USA | CS | 7,124 | V/H: self-report single question binary | 4.10 (3.70, 4.60) |
| Xie et al. (2021); China### | PCS | 6,563 | V/H: self-report single question with Likert scale | 55.67 (54.40, 56.90) |
| Harada et al. (2008); Japan | CS | 843 | V:<6/12 better eye  H: >30dB 1kHz (ear not specified) | 9.73 (7.81, 11.93) |
| Yorgasson et al. (2022); USA | PCS | 6,338 | V/H: self-report multiple questions | 2.20 (1.90, 2.60) |
| Assi et al. (2020); USA | CS | 10,783 | V/H: self-report single question with Likert scale | 19.98 (19.20, 20.70) |
| Kileen et al. (2022)##; USA | PCS | 7,593 | V/H: self-report multiple questions | 3.30 (2.90, 3.80) |
| Mah et al. (2020); Malaysia | CS | 210 | V: <6/12 better eye  H: >25dB PTA better ear | 10.50 (6.70, 15.40) |
| Steinman et al. (2021)##; USA | PCS | 4,636 | V/H: self-report multiple questions | 2.40 (2.00, 2.90) |
| Maruta et al. (2020); Japan | RCS | 2,190 | V: visual acuity chart (not clear type)  H: self-report single question categorical | 13.50 (12.10, 15.00) |
| Haanes et al. (2021); Denmark | CS | 74 | V: <6/12 better eye, and self report  H: >25dB better ear, and self-report | 22.00 (12.90, 32.70) |
| **Clinic, care home or other based** | | | | |
| Davidson et al. (2019); Canada^ | CS; care home | 352,656 | V/H: self-report single categorical question | 20.47 (20.34, 20.60) |
| Guthrie et al. (2016); Canada^ | CS; home care | 218,850 | V/H: self-report single categorical question | 16.71 (16.56, 16.87) |
| Soto-Perez-de-Celis et al. (2018); USA | CS; clinic | 750 | V/H: self-report single categorical question | 7.33 (5.57, 9.43) |
| Cacchione et al. (2003); USA | CS; clinic | 114 | V: ≤20/70 in better eye (near vision) H: fail whisper voice test | 24.56 (16.98, 33.51) |
| Guthrie et al. (2018); Canada | CS; care home; long term care | 402,402 | V/H: self-report single categorical question | Home care clients: 3.65 (3.58, 3.72)  Long-term care residents: 0.97 (0.91, 1.03) |
| Guthrie et al. (2016); Multiple | CS; home care; long term care | Home care clients: 550,360 Long-term Care residents: 261,296 | V: self-report single binary question H: self-report single categorical question | Home care clients:   - Canada = 14.48 (14.39, 14.58), - US = 15.48 (17.7, 16.26), - Belgium = 8.81 (8.36, 9.28) - Finland 13.39 (12.86, 13.93)   Long term care residents:   - Canada = 25.80 (25.60, 26.00), - US=9.73 (9.51, 9.96), - Belgium = 33.86 (30.49, 37.36) - Finland = 22.17 (24.14, 23.24) |
| Lyu et al. (2018); South Korea | CS | 3,381 | V/H: self-report single question with Likert scale | 5.85 (5.12, 6.63) |
| Shakarchi et al. (2021), USA | PCS | 7,648 | V/H: self-report single question with Likert scale | 7.33 (5.57, 9.43) |
| Morandi et al. (2021); Italy$ | CS; O | 3,038 | V/H: clinician judgement | 9.74 (8.71, 10.85) |
| ≥70 years | | | | |
| **Population-based** | |  |  |  |
| Clark et al. (1999); Australia*** | CS | 1,052 | V: <6/12 eye not specified H: >40dB PTA better ear | 9.70 (7.97, 11.65) |
| Kiely et al. (2018); Australia | CS | 1,393 | V: <6/12 eye not specified H: self-report single question with Likert scale | 4.88 (3.81, 6.15) |
| Bergman et al. (2001); Sweden | PCS | 954 | V: <6/7.5 in better eye H: >=40dB PTA in better ear; | 2.29 (1.40, 3.40) |
| Brennan et al. (2005); USA**  Brennan et al. (2006); USA** | PCS | 5,151 | V/H: self-report single categorical question | 21.00 (19.90, 22.14) |
| Crews et al. (2004); USA** | PCS | 9,447 | V/H: self-report single binary question | 8.20 (7.66, 8.78) |
| Green et al. (2013); USA | RCS | 2,000 | V: <6/12 eye not specified H: self-report single question binary | 3.05 (2.34, 3.90) |
| Tinetti et al. (1995); USA | PCS | 1,103 | V: >50% impaired on chart (unclear) H: fail whisper voice test (> 5 words missed) | 11.00 (9.06, 13.20) |
| ≥75 years | | | | |
| **Population-based** | | | | |
| Lupsakko et al. (2002); Finland | CS | 470 | V: <6/15 eye not specified H: self-report single question binary | 7.02 (4.88, 9.72) |
| Bouscaren et al. (2019); France | PCS | 4,010 | V/H: self-report single binary question | 1.7 |
| Pabst et al. (2021); Germany | PCS | 2,051 | V/H: self-report multiple questions | 6.50 (5.80, 7.40) |
| Hwang et al. (2020); USA | PCS | 3,497 | V/H: self-report single question with Likert scale | 5.10 (4.20, 6.10) |
| **Clinic-based** | | | | |
| Grue et al. (2009); Multiple | CS; clinic | 770 | V/H: self-report single categorical question | Pooled: 20.1 (pooled)   - Denmark: 45.00 (37.14, 53.05) - Finland: 9.80 (5.31, 16.13) - Iceland: 17.80 (12.19, 24.73) - Norway: 10.60 (6.31, 16.47) - Sweden: 15.60 (10.37, 22.20) |
| ≥80 years | | | | |
| **Population-based** | |  |  |  |
| Zhang et al. (2020); China | PCS; clinic | 8,788 | V/H: self-report single categorical question | 9.79 (9.17, 10.43) |
| **Non population-based** | |  |  |  |
| Haanes et al. (2014); Norway | CS; care home | 100 | V: <6/15 better eye H: >40dB PTA better ear | 28.00 (19.14, 38.22) |
| ≥95 years | | | | |
| Cimarolli et al. (2014); USA | CS; clinic | 119 | V/H: self-report single question with Likert scale | 37.61 (28.82, 47.03) |
| Other | | | | |
| Dupuis et al. (2014); Canada | CS | 301 | V: <6/12 better eye H: ≥26 PTA better ear | Distance vision only: 7  Distance and near vision: 7.5 |
| Parada et al. (2021) | PCS | 1,383 | V: <6/12 better eye H: > 25dB PTA better ear | 18.1 (16.2, 20.3) |

*CS=cross sectional; PCS=prospective cohort study; RCS=retrospective cohort study; pop=population-based; V=vision; H=hearing; PTA=pure tone audiometry ARMD=age related macular degeneration;*

^Residential Assessment for Home Care (RAI-HC) study - different years (reported separately)

^^National Health And Nutrition Examination Survey (NHANES) – different sample size, different definition (reported separately)

^^^Korean Health And Nutrition Examination Survey (KHANES) – different age groups (reported separately)

* Canadian Longitudinal Study on Ageing (CLSA) – same wave, different measurement of DSI (reported separately)

** National Health Interview Survey; Longitudinal Supplement on Ageing (NHIS/LSOA) – different years, or different age groups (reported separately)

***Australian Longitudinal Study of Ageing (ALSA) – different age groups (reported separately)

# Blue Mountains Eye Study (BMES) – different age groups and samples (reported separately)

## National Health and Ageing Trends (NHATS) – different waves (reported separately)

### China Health and Retirement Longitudinal Study (CHARLS) – different waves, and age groups (reported separately)

& Same population-based survey (India) – different age groups (reported separately)

$ prevalence reported in a subpopulation

**S2 Table:** Reports Measuring Psychosocial Health Outcomes for people with dual sensory impairment (DSI)

| Study author, year | Country, Region | Country income group (at time of publication) | Study design | Study setting | Age group (years) | Sample size total (n DSI) | Comparator group  **No DSI:** people with HI only, VI only, or neither HI nor VI  **Single impairment:** people with HI only or VI only  **HI only:** people with HI only  **VI only:** people with VI only  **No SI**: people with no HI or VI | Definition of DSI | Outcome category | Result summary (outcome in DSI group relative to other group) |
| --- | --- | --- | --- | --- | --- | --- | --- | --- | --- | --- |
| Amini, 2010 | Iran, North Africa and Middle East | Upper middle | Cross sectional | Other | Adults (18+) | 248 (not stated) | No comparator group | V/H: Not specified | Quality of life | No comparator group |
| Armstrong, 2016 | USA, North America | High | Cross sectional | Population | Adults (18+) | 2006: 2,481 (70);  2010: 3,853 (117) | No SI; HI only; VI only | V/H: self-report single binary question | Depression; Quality of life | Varied |
| Assi, 2021 | USA, North America | High | Cross sectional | Population | Older adults (65+) | 7,124 | No SI; Single impairment | V/H: self-reported single question binary | Depression; Cognitive disorders | Worse |
| Bodsworth, 2011 | UK, Western Europe | High | Cross sectional | Register | Adults (18+)) | 539 (539) | Compared with data from other studies on people without VI/HI, and people with HI or people with VI | V/H: Not specified | Psychological wellbeing | Worse |
| Byeon, 2021 | South Korea | High | Prospective cohort | Population | Older adults (60+) | 6,520 (2631) | No SI; single impairment | V/H: self-reported single question Likert | Cognitive disorders | Worse |
| Cacchione, 2003 | USA, North America | High | Cross sectional | Clinic | Older adults (65+) | 114 (28) | No DSI | V: <=20/70 better eye (near vision) H: Fail whisper voice test | Acute confusion | Worse |
| Capella-McDonnall, 2005 | USA, North America | High | Cross sectional | Population | Older adults (55+) | 9,832 (447) | No SI; HI only; VI only | V: self-report single binary question H: self-report single categorical question | Depression | Worse |
| Chia, 2006 | Australia, Australasia | High | Prospective cohort | Population | Older adults (55+) | 2,015 (116) | No SI; single impairment | V: <6/12 better eye H: >25dB PTA better ear | Quality of life | Worse |
| Chou, 2004 | Hong Kong, Southeast Asia, East Asia, and Oceania | High | Cross sectional | Population | Older adults (60+) | 2,003 (131) | No DSI | V/H: self-report single categorical question | Depression | Worse |
| Chou, 2008 | England, UK, Western Europe | High | Prospective cohort | Population | Older adults (65+) | 3,782 (not stated) | No DSI | V/H: self-report single question with Likert scale | Depression | No difference |
| Cimarolli, 2018 | USA, North America | High | Cross sectional | Population | Older adults (95+) | 119 (not stated) | No DSI | V/H: self-report single question with Likert scale | Depression | Worse |
| Cosh, 2018 | Norway, Western Europe | High | Prospective cohort | Clinic | Older adults (60+) | 2,890 (146) | No DSI | V: <6/9 eye not specified H: self-report single question binary | Depression Neurotic and stress related disorders | Varied |
| Dalby, 2009 | Canada, North America | High | Cross sectional | Register | All ages | 182 (182) | No comparator group | V/H: self-report multiple question | Cognitive disorders;  Psychological wellbeing | No comparator group |
| Dammeyer , 2010 | Denmark, Western Europe | High | Cross sectional | Other | Adults (18+)) | 95 (95) | No comparator group | V/H: Not specified | Depression; Neurotic and stress related disorders; Schizophrenia, schizotypical and delusional;  Developmental disorder;  Behavioural disorder/symptoms | No comparator group |
| Dammeyer, 2010 | Denmark, Western Europe | High | Cross sectional | Population | Adults (18+)) | 117 (117) | No comparator group | V: <=6/60 in better eye H: 3FA (500,1000,2000) >=80dB; ear not specified | Self-evaluation of life/satisfaction with life | No comparator group |
| Dammeyer, 2014 | Denmark, Western Europe | High | Cross sectional | Deafblindness centre | Children (<18 years) | 71 (71) | Children with another developmental disorder (from other studies) | V: <6/60 eye not specified H: Deaf >80dB*; Residual hearing <=80dB; ear not specified; frequencies/average not specified | Developmental disorder | No difference |
| Davidson, 2019 | Canada, North America | High | Cross sectional | Care home | Older adults (65+) | 352,656 (72,188) | No DSI | V/H: self-report single categorical question | Cognitive disorders;  Depression; Loneliness | Worse |
| de la Fuente, 2019 | England, UK, Western Europe | High | Prospective cohort | Population | Older adults (60+) | 3,508 (239) | No SI; | V/H: self-report single question with Likert scale | Cognitive disorders | Worse |
| Deepthi, 2012 | India, South Asia | Low to middle | Cross sectional | Population | Older adults (60+) | 257 (257) | No comparator group | V: <6/18 better eye H: >25dB PTA better ear | Cognitive disorders | Study too small to judge |
| Ehn, 2018 | Sweden, Western Europe | High | Case control study | Register | Adults (18+)) | 47 (47) | No SI (reference group from the population) | V: VA chart used but definition not clear H: Based on PTA but definition not clear | Psychological wellbeing | Worse |
| Fuller, 2018 | USA, North America | High | Cross sectional | Population | Older adults (45+) | 7,210,535 (not stated) | No SI; HI only; VI only | V/H: self-report single binary question | Cognitive disorders | Worse |
| Ge, 2021 | USA, North America | High | Prospective cohort | Population | Older adults (65+) | 295 (52) | No DSI | V: <6/12 better eye  H: >25dB better ear, any frequency | Cognitive disorders | Worse |
| Gopinath, 2013 | Australia, Australasia | High | Prospective cohort | Population | Older adults (55+) | 2,812 (947) | No DSI | V: <6/12 better eye H: >25dB PTA better ear | Cognitive disorders | Worse |
| Guthrie, 2016 | Multiple, Multiple | High | Cross sectional | Other | Older adults (65+) | Home care clients: 550,360 (80,634) Long-term care residents: 261,296 (56,401) | No DSI | V: self-report single binary question H: self-report single categorical question | Depression | Varied |
| Guthrie, 2016 | Canada, North America | High | Cross sectional | Care home | Older adults (65+) | 218,850 (37,047) | No DSI | V/H: self-report multiple question | Depression | Worse |
| Hajek, 2020 | Germany, Western Europe | High | Prospective cohort | Population | Older adults (40+) | 5,138 (815) | No DSI | V/H: self-reported single question binary | Depression; Self-evaluation of life/satisfaction with life; Loneliness; Other: social isolation, self-esteem and autonomy | Worse |
| Han, 2019 | South Korea, Asia Pacific | High | Cross sectional | Population | Older adults (45+) | 5,832 (not stated) | No SI | V/H: self-report single question with Likert scale | Depression | Worse |
| Harada, 2008 | Japan, Asia Pacific | High | Cross sectional | Population | Older adults (65+) | 843 (82) | No SI | V: <6/12 better eye H: >30dB at 1k better ear | Depression | Worse |
| Harithasan, 2020 | Malaysia, Southeast Asia, East Asia, and Oceania | Upper middle | Cross sectional | Population | Older adults (60+) | 229 (19) | No DSI | V: <6/12 better eye H: >=26 PTA better ear | Depression;  Cognitive disorders;  Loneliness;  Quality of life | Varied |
| Harsthorne, 2007 | USA, North America | High | Cross sectional | Register | Children (<18 years) | 98 (55) | Children with CHARGE who were not deafblind | V/H: Parent-report that child was deaf and blind | Cognitive disorders | Varied |
| Hartshorne, 2009 | England, UK, Western Europe | High | Cross sectional | CHARGE syndrome foundation mailing list | Children (<18 years) | 87 (18) | Children with CHARGE who were not deafblind | V/H: Self-reported ""no better than both moderate hearing impairment in the best ear and moderate vision impairment in the best eye" | Sleep disturbance | Worse |
| Heine, 2019 | China, Southeast Asia, East Asia, and Oceania | Upper middle | Cross sectional | Population | Older adults (60+) | 8,268 (not stated) | No DSI | V/H: self-report single question with Likert scale | Depression;  Self-evaluation of life/satisfaction with life | Worse |
| Heine, 2019 | Australia, Australasia | High | Retrospective cohort | Population | Older adults (65+) | 1,000 (110 in 1994; 50 in 2004) | No SI | V/H: self-report single question categorical | Depression | Worse |
| Hersh, 2013 | Multiple, Western Europe | High | Qualitative | Register | Unknown | 27 (27) | No comparator group | V/H: Not specified | Quality of life | No comparator group |
| Ho, 2021 | Singapore | High | Cross sectional | Care home | Older adults (40+) | 123 (97) | No DSI | V: <6/12 better eye  H: PTA >40dB better ear | Quality of life | Worse |
| Hong, 2016 | Australia, Australasia | High | Prospective cohort | Population | Older adults (49+) | 3,654 (93) | No SI | V: <6/12 worse eye H: >40dB in better ear | Cognitive disorders | No difference |
| Hovaldt, 2022 | Denmark, Western Europe | High | Cross sectional | Register | Older adults (50+0 | 290 (290) | No comparator group | V/H: not specified (register) | Thoughts of self-harm or suicide | No comparator group |
| Huddle, 2016 | USA, North America | High | Cross sectional | Population | Older adults (70+) | 1,669 (291) | No SI | V: self-report single question binary H: >=25dB PTA in better ear | Psychological wellbeing | Worse |
| Hwang, 2020 | USA, North America | High | Prospective cohort | Population | Older adults (75+) | 2,051 (104) | No DSI | V/H: self-reported multiple questions | Cognitive disorders | Varied |
| Khil, 2015 | Germany, Western Europe | High | Cross sectional | Population | 25-74 | 1,102 (187) | No SI (no visual/auditory/olfactory/gustatory impairment) | V: <6/12 worse eye H: >30dB 3FA in worse ear | Quality of life | Varied |
| Khurana, 2021 | England, Western Europe | High | Cross sectional | Population | 16+ | 7,546 (281) | No DSI | V: self-report single question Likert  H: self-report single question binary | Suicide ideation and suicide attempt | Worse |
| Kiely, 2013 | Australia, Australasia | High | Prospective cohort | Population | Older adults (65+) | 1,611 (not stated) | No SI | V: <6/12 better eye H: >25dB PTA better ear | Depression | Worse |
| Kiely, 2018 | Australia, Australasia | High | Cross sectional | Population | 72-79 | 1,393 (68) | No SI | V: <6/12 eye not specified H: >25dB PTA better ear | Psychological wellbeing | Varied |
| Killeen, 2022 | USA, North America | High | Prospective cohort | Population | Older adults (65+) | 7,593 (331) | No DSI | V/H: self-report multiple questions | Depression | Worse |
| Kim, 2015 | South Korea, Asia Pacific | High | Cross sectional | Population | Older adults (65+) | 3,636 (224) | No SI | V: <6/18 worse eye H: >40dB in better ear | Psychological wellbeing | Worse |
| Kuo, 2021 | USA, North America | High | Cross sectional | Population | Older adults (65+) | 7,562 (303) | No DSI | V/H: self-report multiple questions | Cognitive disorders | Worse |
| Kwan, 2022 | Hong Kong, Asia Pacific | High | Prospective cohort | Care home | Older adults (60+) | 2,233 (273) | No DSI | V/H: self-report single question | Cognitive disorders | Worse |
| Kwon, 2015 | South Korea, Asia Pacific | High | Cross sectional | Population | Older adults (60+) | 5,260 (268) | No SI | V: <6/18 better eye H: >40dB PTA better ear | Depression;  Neurotic and stress related disorders | The same |
| Lach, 2019 | Not specified, Not specified | Unknown | Cross sectional | Care home | Older adults (50+) | 225 (67) | No DSI | V: <6/15; eye not specified H: >=40dB PTA better ear | Depression; Neurotic and stress related disorders; Cognitive disorders | Varied |
| Lehane, 2018 | Denmark, Western Europe | High | Cross sectional | Population | Adults (18+) | 316 (183) | HI only; VI only | V: <6/9 eye not specified H: >=26 db PTA in better ear | Psychological wellbeing | No difference |
| Liljas, 2018 | England, UK, Western Europe | High | Prospective cohort | Population | Older adults (50+) | 4,621 (179) | No SI | V/H: self-report single question with Likert scale | Depression | Worse |
| Lin, 2004 | USA, North America | High | Cross sectional | Population | Older adults (65+) | 6,112 (not stated) | No DSI | V: <6/12 in better eye H: >=40dB at 2kHz in better ear | Cognitive disorders | Worse |
| Liu, 2022 | China, Asia Pacific | Upper middle | Prospective cohort | Population | Older adults (40+) | 13,690 (1,376) | No DSI | V/H: self-report multiple questions | Depression | Worse |
| Loprinzi, 2013 | USA, North America | High | Cross sectional | Population | Adults (18+)) | 567 (17) | No SI | V/H: self-report single question with Likert scale | Depression | Worse |
| Luo, 2018 | China, Southeast Asia, East Asia, and Oceania | Upper middle | Cross sectional | Population | Older adults (50+) | 250,752 (5,277) | No DSI | V: <6/120 better eye H: >40dB in better ear | Cognitive disorders | Worse |
| Lupsakko, 2002 | Finland, Western Europe | High | Cross sectional | Population | Older adults (75+) | 470 (33) | No SI | V: <6/15 better eye H: self-report single question binary | Depression | Varied |
| Lyu, 2018 | South Korea, Asia Pacific | High | Cross sectional | Population | Older adults (50+) | 3,831 (not stated) | No SI | V/H: self-report single question with Likert scale | Depression;  Cognitive disorders | Varied |
| Ma, 2021 | China, Asia Pacific | Upper middle | Prospective cohort | Population | Older adults (45+) | 13,097 (723) | No DSI | V/H: self-report, single question Likert | Episodic memory | Worse |
| Mah, 2020 | Malaysia | Upper middle | Cross sectional | Population | Older adults (65+) | 210 (22) | No DSI | V: <6/12 better eye  H: >25dB PTA better ear | Cognitive disorders | Worse |
| Maharani, 2018 | Multiple, Multiple | High | Cross sectional | Population | Older adults (50+) | 45,805 (not stated) | No SI | V/H: self-report single question with Likert scale | Cognitive disorders | Worse |
| Maharani, 2020 | USA, North America | High | Cross sectional | Population | Older adults (50+) | 19,618 (984) | No SI | V/H: self-report single question with Likert scale | Cognitive disorders | Worse |
| Marmamula, 2021 | India, South Asia | Lower middle income | Cross sectional | Care home | Older adults (60+) | 897 (50) | No DSI | V:<6/18 better eye  H: self-report multiple questions | Depression | Worse |
| Maruta, 2019 | Japan, Asia Pacific | High | Retrospective cohort | Register | Older adults (50+) | 2,190 (295) | No SI | V: VA chart definition not clear H: self-report single categorical question | Cognitive disorders | Worse |
| Maruta, 2020 | Japan, Asia Pacific | High | Retrospective cohort | Population | Older adults (65+) | 2,190 | No DSI | V: VA chart definition not clear H: self-report single categorical question | Cognitive disorders | Worse |
| McDonnall, 2009 | USA, North America | High | Retrospective cohort | Population | Older adults (50+) | 2,689 (not stated) | No SI | V/H: self-report single question with Likert scale | Depression | Worse |
| McDonnall, 2011 | USA, North America | High | Prospective cohort | Population | Older adults (50+) | 2,688 (not stated) | No SI | V/H: self-report single question with Likert scale | Depression | Worse |
| McDonnall, 2011 | USA, North America | High | Prospective cohort | Population | Older adults (50+) | 2,688 (not stated) | No SI | V/H: self-report single question with Likert scale | Depression | Varied |
| Meuwese- Jongejeugd, 2008 | The Netherlands, Western Europe | High | Cross sectional | Clinic | Adults (18+) | 1,359 (77) | Compared severity of ID, and whether or not had Down's Syndrome | V: <6/12 better eye  H: >25dB PTA better ear | Intellectual disability | Worse |
| Michalowsky, 2019 | Germany, Western Europe | High | Case control | Clinic | Older adults (65+) | 122,708 (not stated) | People without dementia | V/H: Not specified | Cognitive disorders | No difference |
| Mick, 2018 | Canada, North America | High | Cross sectional | Population | 45-85 | 21,241 (not stated) | No DSI | V/H: self-report single question with Likert scale | Loneliness | Worse |
| Mitoku, 2016 | Japan, Asia Pacific | High | Prospective cohort | Population | Older adults (50+) | 1,754 (320) | No SI | V: VA chart definition not clear H: self-report single categorical question | Cognitive disorders | Worse |
| Morandi, 2021 | Italy, Western Europe | High | Cross sectional | Hospital | Older adults (65+) | 3,038 (296) | No DSI | V/H: clinician judgement | Delirium | Worse |
| Mudie, 2018 | USA, North America | High | Cross sectional | Clinic | Older adults (50+) | 220 (42) | No SI; HI only; VI only | V: Mean deviation on visual field testing worse than -5dB better eye H: >25dB better ear | Cognitive disorders | No difference |
| Pabst, 2021 | Germany, Western Europe | High | Prospective cohort | Population | Older adults (75+) | 3,497 (229) | No DSI | V/H: self-report single question Likert | Cognitive disorders | No difference |
| Parada, 2021 | USA, North America | High | Prospective cohort | Population | Adults (not specified) | 1,383 (251) | No DSI | V: <6/12 better eye  H: >25dB PTA better ear | Cognitive disorders | Worse |
| Pardhan, 2021 | Spain, Western Europe | High | Prospective cohort | Population | 15+ | 23,089 (908) | No DSI | V/H: self-report single question categorical | Depression; anxiety | Worse |
| Pardhan, 2020 | Spain, Western Europe | High | Prospective cohort | Population | 15+ | 23,089 (908) | No DSI | V/H: self-report single question categorical | Depression; anxiety | Worse |
| Phua, 2022 | Singapore | High | Cross sectional | Population | Older adults (60+) | 4,077 (523) | No DSI | V/H: self-report single question Likert | Depression; Loneliness; Psychological wellbeing; Quality of life | Worse |
| Rong, 2020 | China, Asia Pacific | Upper middle | Cross sectional | Population | Older adults (45+) | 18,038 (10,575) | No DSI | V: self-report multiple questions  H: self-report single question Likert | Depression; Cognitive disorders | Worse |
| Simning, 2018 | USA, North America | High | Prospective cohort | Population | Older adults (65+) | 7,507 (125) | No S | V/H: self-report single binary question | Depression;  Neurotic and stress related disorders | Worse |
| Soto-Perez-de-Celis, 2018 | USA, North America | High | Cross sectional | Clinic | Older adults (65+) | 750 (55) | No SI | V/H: self-report single categorical question | Depression;  Neurotic and stress related disorders | Worse |
| Teh, 2006 | Singapore, Asia Pacific | High | Retrospective cohort | Clinic | Older adults (50+) | 112 (36) | No DSI | V: self-report single question binary H: Fail whisper voice test | Depression;  Cognitive disorders | Varied |
| Turunen-Tahari, 2017 | Sweden, Western Europe | High | Retrospective cohort | Register | Adults (18+)) | 2,319 (543) | HI only | V: self-report single question binary H: >70dB PTA in better ear | Depression;  Quality of life | Worse |
| UrquetaAlfaro, 2020 | Canada, North America | High | Cross sectional | Clinic | Older adults (65+) | 200 (73) | HI only; VI only | V/H: self-report single question categorical | Depression; Loneliness; Psychological wellbeing | No difference |
| Viljanen, 2013 | Finland, Western Europe | High | Prospective cohort | Finnish Twin Cohort (same-sex twins) | 63 to 76-year-old women twins | 434 (not stated) | No SI (no vision/hearing/balance difficulties) | V/H: self-report single question categorical | Neurotic and stress related disorders;  Self-evaluation of life/satisfaction with life | Worse |
| Wahl, 2013 | Germany, Western Europe | High | Cross sectional | Clinic | 75-94 | 430 (43) | No SI | V: <6/18 worse eye H: >=35dB PTA in better ear | Cognitive disorders;  Loneliness;  Psychological wellbeing | Varied |
| Xie, 2021 | China, Asia Pacific | Upper middle | Prospective cohort | Population | Older adults (65+) | 6,353 (3537) (baseline) 2,703 (followup) | No DSI | V/H: self-report single question Likert | Depression | Worse |
| Yamada, 2014 | Multiple, Western Europe | High | Cross sectional | Care home | Older adults (50+) | 4,007 (1,275) | No SI | V/H: self-report single categorical question | Depression;  Cognitive disorders | Worse |
| Yamada, 2015 | Multiple, Western Europe | High | Prospective cohort | Care home | Older adults (50+) | 4,156 (400) | No SI | V/H: self-report single categorical question | Behavioural disorder/symptoms | Worse |
| Yamada, 2016 | Multiple, Western Europe | High | Prospective cohort | Care home | Older adults (50+) | 1,989 (122) | No SI; Single impairment | V/H: self-report single categorical question | Cognitive disorders | Worse |
| Yorgason, 2022 | USA, North America | High | Cross sectional | Population | Older adults (65+) | 3,338 (140) | No DSI | V/H: self-report multiple questions | Cognitive disorders | Worse |
| Zhao, 2021 | China, Asia Pacific | High | Cross sectional | Population | Older adults (40+) | 13,914 (294) | No DSI | V/H: self-report single question categorical | Cognitive disorders | Worse |

**S3 Table**: Reports measuring participation outcomes for people with dual sensory impairment (DSI)

| Study author, year | Country, Region | Country income group (at time of publication) | Study design | Study setting/recruitment | Age group (years) | Sample size (n DSI) | Comparator group  **No DSI:** people with HI only, VI only, or neither HI nor VI  **Single impairment:** people with HI only or VI only  **HI only:** people with HI only  **VI only:** people with VI only  **No SI**: people with no HI or VI | Definition of DSI | Outcome measured | Result summary (outcome in DSI group compared to other group) |
| --- | --- | --- | --- | --- | --- | --- | --- | --- | --- | --- |
| Assi, 2020 | USA, North America | High | Cross sectional | Population | All ages | 10,783 (2,154) | No DSI | V/H: self-report single question Likert | Activities of daily living | Worse |
| Assi, 2021 | USA, North America | High | Cross sectional | Population | Older adults (65+) | 7,124 (293) | No SI; Single impairment | V/H: self-reported single question binary | Self care activities; household activities | Worse |
| Beall, 1986 | Nepal, South Asia | Low to middle | Cross sectional | Population | Older men (50+) | 117 (not stated) | HI only; VI only | V: VA <=6/60 better eye H: >=60dB; frequencies/average NS; ear NS | Activities of daily living | Worse |
| Bouscaren, 2019 | France, Western Europe | High | Cross sectional | Population | Older adults (75+) | 4,010 (not stated; 1.7%) | No SI | V/H: self-report single binary question | Activities of daily living | Worse |
| Brennan, 2005  Brennan 2006 | USA, North America | High | Prospective cohort | Population | Older adults (70+) | 5,151 (390) | No SI; HI only; VI only | V/H: self-report single categorical question | Activities of daily living | Worse |
| Campbell, 1999 | USA, North America | High | Cross sectional | Population | Older adults (65+) | 8,767 (675) | No SI | V/H: self-report multiple question | Activities of daily living; Participation | Worse |
| Chou, 2004 | Hong Kong, Southeast Asia, East Asia, and Oceania | High | Cross sectional | Population | Older adults (60+) | 2,003 (131) | No DSI | V/H: self-report single categorical question | Activities of daily living | Worse |
| Cimarolli, 2014 | USA, North America | High | Cross sectional | Population | Older adults (95+) | 119 (44) | No DSI | V/H: self-report single question with Likert scale | Activities of daily living | Worse |
| Cimarolli, 2018 | USA, North America | High | Cross sectional | Population | Older adults (95+) | 119 (not stated) | No DSI | V/H: self-report single question with Likert scale | Activities of daily living | Worse |
| Clark, 1999 | Australia, Australasia | High | Cross sectional | Population | Older adults (70+) | 1,052 (102) | No SI; HI only; VI only | V: <6/12 eye not specified H: >40dB PTA better ear | Activities of daily living;  Social participation | No difference |
| Crews, 2004 | USA, North America | High | Prospective cohort | Population | Older adults (70+) | 9,447 (779) | No SI | V/H: self-report single binary question | Activities of daily living | Worse |
| Crowe, 2018 | Denmark, Western Europe | High | Cross sectional | Register | Older adults (50+) | 513 (513) | Compared severity of loss (vision and hearing separately) | V/H: not specified | Communication participation | Varied |
| Dalby, 2009 | Canada, North America | High | Cross sectional | Register | All ages | 182 (182) | No comparator group | V/H: self-report multiple question | Communication | No comparator group |
| Deepthi, 2012 | India, South Asia | Low to middle | Cross sectional | Population | Older adults (60+) | 257 (257) | No comparator group | V: <6/18 better eye H: >25dB PTA better ear | Activities of daily living | No comparator group |
| Ehn, 2018 | Sweden, Western Europe | High | Case control | Register | Adults (18+)) | 47 (47) | No SI (reference group from the population) | V: VA chart used but definition not clear H: Based on PTA but definition not clear | Activities of daily living; social participation | Worse |
| Figueiredo, 2013 | Brazil, Latin America | Upper middle | Case series | Clinic | 20-57 years | 11 (11) | No comparator group | V/H: Clinical diagnosis of Ushers syndrome | Communication; work and leisure activities | No comparator group |
| Fletcher, 2013 | Canada, North America | High | Qualitative | Population | 44-88 years | 7 (7) | No comparator group | V/H: not specified | Activities of daily living; communication; independence; self-regulation (goal pursuit) | No comparator group |
| Fuller, 2018 | USA, North America | High | Cross sectional | Population | Older adults (45+) | 7,210,535 (not stated) | No SI; HI only; VI only | V/H: self-report single binary question | Activities of daily living; Independence | Worse |
| Gopinath, 2017 | Australia, Australasia | High | Prospective cohort | Population | Older adults (55+) | 2,956 (544) | No SI | V: <6/12 better eye H: >25dB in better ear | Retirement | No difference |
| Grue, 2009 | Multiple, Western Europe | High | Cross sectional | Clinic | Older adults (75+) | 770 (155) | No DSI | V/H: self-report single categorical question | Activities of daily living | No difference |
| Guthrie, 2018 | Canada, North America | High | Cross sectional | Other | Older adults (65+) | 402,402 (11,940) | No SI; HI only; VI only | V/H: self-report single categorical question | Activities of daily living | Worse |
| Haanes, 2014 | Norway, Western Europe | High | Cross sectional | Care home | Older adults (80+) | 93 (26) | No comparator group | V: <=6/15 better eye H: >40dB PTA better ear | Activities of daily living; Independence | No comparator group |
| Hajek, 2020 | Germany, Western Europe | High | Prospective cohort | Population | Older adults (40+) | 5,138 (815) | No DSI | V/H: self-reported single question binary | Social isolation | Worse |
| Harada, 2008 | Japan, Asia Pacific | High | Cross sectional | Population | Older adults (65+) | 843 (82) | No SI | V: <6/12 better eye H: >30dB at 1k better ear | Activities of daily living | Worse |
| Heine, 2019 | Australia, Australasia | High | Retrospective cohort | Population | Older adults (65+) | 1,000 (110 in 1994; 50 in 2004) | No SI | V/H: self-report single question categorical | Activities of daily living; Social participation; Independence | Worse |
| Heine, 2019 | China, Southeast Asia, East Asia, and Oceania | Upper middle | Cross sectional | Clinic | (Adults (18+)) | 8,268 (4,298) | No DSI | V/H: self-report single question with Likert scale | Activities of daily living | Worse |
| Heyl, 2012 | Germany, Western Europe | High | Cross sectional | Population | Older adults (75+) | 430 (43) | No SI; HI only; VIonly | V: <6/12 better eye H: >=35dB PTA in better ear | Activities of daily living | Worse |
| Jaiswal, 2019 | Iran, North Africa and Middle East | Low to middle | Qualitative | Population | (Adults (18+)) | 16 (16) | No comparator group | V/H: Formal diagnosis of deafblindness (method not specified) | Social participation;  Independence; Self-regulation (goal pursuit) | No comparator group |
| Keller, 1999 | USA, North America | High | Cross sectional | Clinic | Older adults (55+) | 576 (75) | No SI | Vision: <=20/70 in better eye (near vision) Hearing: Fail whisper voice test | Activities of daily living | Worse |
| Kwon, 2015 | South Korea, Asia Pacific | High | Cross sectional | Population | Older adults (60+) | 5,260 (268) | No SI | V: <6/18 better eye H: >40dB PTA better ear | Activities of daily living | Worse |
| Lach, 2019 | Not specified, Not specified | Unknown | Cross sectional | Care home | Older adults (50+) | 225 (67) | No DSI | V: <6/15; eye not specified H: >=40dB PTA better ear | Social participation | Worse |
| Lee, 2005 | USA, North America | High | Prospective cohort | Population | Older adults (45+) | 60,997 (1,433) | No SI | V/H: self-report single binary question | Activities of daily living | Worse |
| Lehane, 2018 | Denmark, Western Europe | High | Cross sectional | Population | (Adults (18+)) | 316 (183) | HI only; VI only | V: <6/9 eye not specified H: >=26 db PTA in better ear | Communication | Only reported in the DSI group |
| Liljas, 2018 | England, UK, Western Europe | High | Prospective cohort | Population | Older adults (50+) | 4,621 (179) | No SI | V/H: self-report single question with Likert scale | Education;  Wealth | Worse |
| Liu, 2015 | USA, North America | High | Prospective cohort | Population | Older adults (65+) | 3,871 (183) | No SI | V: self-report single binary question H: self-report single categorical question | Activities of daily living | Worse |
| Lupsakko, 2002 | Finland, Western Europe | High | Cross sectional | Population | Older adults (75+) | 470 (33) | No SI | V: <6/15 better eye H: self-report single question binary | Activities of daily living | Worse |
| Mick, 2018 | Canada, North America | High | Cross sectional | Population | 45-85 years | 21,241 (not stated) | No DSI | V/H: self-report single question with Likert scale | Social participation | Worse |
| Moller, 2003 | Sweden, Western Europe | High | Cross sectional | Secondary analysis | Only adults (Adults (18+)) | 27 (27) | No comparator group | V/H: Diagnosis of deafblindness (method not specified) | Participation | No comparator group |
| Mudie, 2018 | USA, North America | High | Cross sectional | Clinic | Older adults (50+) | 220 (42) | No SI; HI only; VI only | V: Mean deviation on visual field testing worse than -5dB better eye H: >25dB better ear | Activities of daily living; social participation | Worse |
| Mueller-Schotte, 2018 | Netherlands, Western Europe | High | Prospective cohort | Population | Older adults (60+) | 9,319 (1,693) | No SI | V/H: self-report single binary question | Activities of daily living | Worse |
| Petrovsky, 2019 | USA, North America | High | Secondary analysis | Care home | Older adults (65+) | 213 (81) | HI only; VI only | V: <=6/15 in better eye H: >40dB at 1kHz in better ear | Social participation | No difference |
| Phua, 2022 | Singapore | High | Cross sectional | Population | Older adults (60+) | 4,077 (523) | No DSI | V/H: self-report single question Likert | Social participation | Worse |
| Raina, 2004 | Canada, North America | High | Cross sectional | Population | Older adults (55+) | 16,613 (not stated) | HI only; VI only | V/H: self-report single binary question | Activities of daily living | Varied |
| Reuben, 1999 | USA, North America | High | Prospective cohort | Population | Older adults (55+) | 5,646 (36) | No SI | V: 6/12 better eye H: >40dB at 1 or/and 2kHz in better ear | Activities of daily living | Worse |
| Roets-Merken, 2017 | Netherlands, Western Europe | High | Qualitative | Care home | 82-98 years | 47 (47) | No comparator group | V:best-corrected visual acuity of <=0.3 diopter or with a visual field of <30 H: >40dB PTA better ear | Participation; Independence | No comparator group |
| Shakarchi, 2020 | USA. North America | High | Cross sectional | Population | Older adults (50+) | 13,092 (1,061) | No DSI; no SI; VI only; HI only | V/H: self-report single question Likert | Every day discrimination | Worse |
| Simning, 2018 | USA, North America | High | Prospective cohort | Population | Older adults (65+) | 7,507 (125) | No SI | V/H: self-report single binary question | Activities of daily living | Worse |
| Soto-Perez-de-Celis, 2018 | USA, North America | High | Cross sectional | Clinic | Older adults (65+) | 750 (55) | No SI | V/H: self-report single categorical question | Activities of daily living | Worse |
| Tareque, 2019 | Singapore, Asia Pacific | High | Prospective cohort | Population | Older adults (60+) | 3,452 (401) | No SI | V/H: self-report single question with Likert scale | Activities of daily living | Worse |
| Teh, 2006 | Singapore, Asia Pacific | High | Retrospective cohort | Clinic | Older adults (50+) | 112 (36) | No DSI | V: self-report single question binary H: Fail whisper voice test | Activities of daily living | Worse |
| Tinetti, 1995 | USA, North America | High | Prospective cohort | Population | Older adults (70+) | 927 (11) | No SI | V: >50% impaired on VA chart (unclear) H: Fail whisper voice test (> 5 words missed) | Activities of daily living | No difference |
| Viljanen, 2014 | Multiple, Western Europe | High | Cross sectional | Population | Older adults (50+) | 27,536 (not stated; 4.0%) | No SI | V/H: self-report single categorical question | Social participation | Worse |
| Wahl, 2013 | Germany, Western Europe | High | Cross sectional | Clinic | 75-94 | 430 (43) | No SI | V: <6/18 worse eye H: >=35dB PTA in better ear | Activities of daily living; social participation; self-regulation (goal pursuit) | Varied |

**S4 Table:** Reports measuring physical health outcomes for people with dual sensory impairment (DSI)

| Study author, year | Country, Region | Country income group (at time of publication) | Study design | Study setting/recruitment | Age group (years) | Sample size (n DSI) | Comparator group  **No DSI:** people with HI only, VI only, or neither HI nor VI  **Single impairment:** people with HI only or VI only  **HI only:** people with HI only  **VI only:** people with VI only  **No SI**: people with no HI or VI | Definition of DSI | Measured outcome | Outcome in DSI group compared to other group |
| --- | --- | --- | --- | --- | --- | --- | --- | --- | --- | --- |
| Appollonio, 1995 | Italy, Western Europe | High | Prospective cohort | Population | Older adults (70-75 years) | 1,303 (8) | No SI; HI only; VI only | V: <=6/15 in better eye H: Fail whisper voice test | Mortality | Worse |
| Crews, 2004 | USA, North America | High | Prospective cohort | Population | Older adults (70+) | 9,447 (779) | No SI | V/H: self-report binary question | General health | Worse |
| Crews, 2017 | USA, North America | High | Cross sectional | Population | Older adults (65+) | 36,110 (not stated) | No VI | V: self-report binary question H: self-report single question | General health | Worse |
| Dalby, 2009 | Canada, North America | High | Cross sectional | Register | All ages | 182 (182) | No comparator group | V/H: self-report multiple question | General health | No comparator |
| Dammeyer, 2010 | Denmark, Western Europe | High | Cross sectional | Population | (Adults (18+)) | 123 (123) | No comparator group | V: <=6/60 better eye H: 3FA (500,1000,2000) >=80dB; ear not specified | General health | No comparator |
| Davidson, 2019 | Canada, North America | High | Cross sectional | Care home | Older adults (65+) | 352,656 (72,188) | No DSI | V/H: self-report single categorical question | Physical functioning | Worse |
| Deardorff, 2019 | USA, North America | High | Prospective cohort | Population | Older adults (65+) | 24,009 (4,375) | No SI | V/H: self-report single categorical question | Health care costs | Worse |
| Deardorff, 2020 | USA, North America | High | Retrospective cohort | Population | Older adults (65+) | 15,999 (NS) | HI only; VI only | V: self-report single question Likert  H: self-report multiple questions | Hospitalisation | Reported only in DSI group |
| Ehn, 2018 | Sweden, Western Europe | High | Case control | Register | Only adults (Adults (18+)) | 47 (47) | No SI (reference group from the population) | V: VA chart used but definition not clear H: Based on PTA but definition not clear | General health | Worse |
| Fisher, 2014 | Iceland, Western Europe | High | Prospective cohort | Population | 67-98 years | 4,926 (343) | No SI | V: <6/15 eye not specified H: >40dB at 1 or/and 2kHz in better ear | Mortality | Varied |
| Fisher, 2016 | USA, North America | High | Cross sectional | Population | Older adults (50+) | 6,654 (316) | No comparator group | V/H: self-report single categorical question | Physical functioning | No comparator |
| Gopinath, 2013 | Australia, Australasia | High | Prospective cohort | Population | Older adults (55+) | 2,812 (947) | No DSI | V: <6/12 better eye H: >25dB PTA better ear | General health;  Mortality | Worse |
| Gopinath, 2014 | Australia, Australasia | High | Prospective cohort | Population | Older adults (55+) | 2,443 (95) | No SI | V: <6/12 better eye H: >25dB PTA better ear | Health behaviours | Worse |
| Gopinath, 2016 | Australia, Australasia | High | Prospective cohort | Population | Older adults (55+) | 1,478 (334) | No SI | V: <6/12 better eye H: >25dB PTA better ear | Physical functioning | Varied |
| Gopinath, 2021 | Australia, Australasia | High | Prospective cohort | Population | Older adults (55+) | 1,085 | No SI | V: <6/12 better eye H: >25dB PTA better ear | Mortality;, successful ageing | Same |
| Graue-Hernández, 2019 | Mexico, Latin America and the Caribbean | Upper middle | Cross sectional | Population | Older adults (50+) | 1,511 (66) | No DSI | V/H: self-report multiple question | General health | Worse |
| Green, 2013 | USA, North America | High | Retrospective cohort | Population | Older adults (70+) | 2,000 (61) | No SI | V: <6/12 eye not specified H: self-report binary | Health behaviours | Varied |
| Grue, 2008 | Norway, Western Europe | High | Cross sectional | Clinic | Older adults (65+) | 332 (not stated; 30.1%) | No comparator group | V: <6/7.5 better eye H: >=30dB PTA in better ear | Physical functioning | No comparator |
| Grue, 2009 | Multiple, Western Europe | High | Cross sectional | Clinic | Older adults (75+) | 770 (155) | No DSI | V/H: self-report single categorical question | Physical functioning | No difference |
| Heine, 2019 | Australia, Australasia | High | Retrospective cohort | Population | Older adults (65+) | 1,000 (110 in 1994; 50 in 2004) | No SI | V/H: self-report single question categorical | General health | Worse |
| Heyl, 2012 | Germany, Western Europe | High | Cross sectional | Population | Older adults (75+) | 430 (43) | No SI; HI only; VI only | V: <6/12 better eye H: >=35dB PTA in better ear | General health | Varied |
| Huddle, 2016 | USA, North America | High | Cross sectional | Population | Older adults (70+) | 1,669 (291) | No SI | V: self-report single question binary H: >=25dB PTA in better ear | General health; health behaviours; hospitalisation | Varied |
| Jin, 2010 | Canada, North America | High | Qualitative | Deaf Blind Association of Ontario | 37-76 years | 7 (7) | No comparator group | Not specified; participants were deafblind | Oral health | No comparator |
| Jung, 2022 | South Korea, Asia Pacific | High | Prospective cohort | Register | 30+ | 77,1128 (8,720) | No DSI | V: <6/12 better eye  H: >40dB PTA at least one ear | Stroke; mortality | Worse |
| Kiely, 2016 | Australia, Australasia | High | Prospective cohort | Population | Older adults (50+) | 4,160 (not stated) | HI only ; VI only | V: <6/12 eye not specified H: >25dB PTA better ear | Mortality | Worse |
| Kulmala, 2009 | Finland, Western Europe | High | Prospective cohort | Population | 63-76 years | 428 (74) | People with good vision | V: <6/60 eye not specified H: >=21 dB PTA in better ear | Physical functioning | Worse |
| Lach, 2019 | Not specified, Not specified | Unknown | Cross sectional | Care home | Older adults (50+) | 225 (67) | No DSI | V: <6/15; eye not specified H: >=40dB PTA better ear | General health; Physical functioning | No difference |
| Lam, 2006 | USA, North America | High | Retrospective cohort | Clinic | Older adults (Adults (18+)) | 116,796 (1,461) | No SI | V/H: self-report binary question | Mortality | Varied |
| Lee, 2007 | USA, North America | High | Retrospective cohort | Population | Adults (18+) | 116,796 (1,461) | No SI | V/H: self-report binary question | Mortality | Varied |
| Lehane, 2017 | Denmark, Western Europe | High | Cross sectional | Register | Older adults (50+) | 45 (45) | Population mean | V/H: Registered as deafblind according to Nordic definition | Sexual health | Worse |
| Liljas, 2018 | England, UK, Western Europe | High | Prospective cohort | Population | Older adults (50+) | 4,621 (179) | No SI | V/H: self-report single question with Likert scale | General health; Physical functioning | Worse |
| Lin, 2004 | USA, North America | High | Cross sectional | Population | Older adults (65+) | 6,112 (not stated) | No DSI | V: <6/12 in better eye H: >=40dB at 2kHz in better ear | Physical functioning | Worse |
| Liu, 2015 | USA, North America | High | Prospective cohort | Population | Older adults (65+) | 3,871 (183) | No SI | V: self-report single binary question H: self-report single categorical question | General health; mortality | Worse |
| Loprinzi, 2013 | USA, North America | High | Cross sectional | Population | 20-84 years | 1,445 (29) | No SI | V: <6/12 better eye H: >25dB PTA better ear | Health behaviours | Worse |
| McDonnall , 2011 | USA, North America | High | Prospective cohort | Population | Older adults (50+) | 2,688 (not stated) | No SI | V/H: self-report single question with Likert scale | Health behaviours | Worse |
| Mitoku, 2016 | Japan, Asia Pacific | High | Prospective cohort | Population | Older adults (50+) | 1,754 (320) | No SI | V: VA chart definition not clear H: self-report single categorical question | Mortality | Worse |
| Miyawaki, 2019 | Japan, Asia Pacific | High | Retrospective cohort | Population | 40-69 years | 9,522 (86) | No comparator group | V/H: self-report single question categorical | Mortality | No comparator |
| Mudie, 2018 | USA, North America | High | Cross sectional | Clinic | Older adults (50+) | 220 (42) | No SI; HI only; VI only | V: Mean deviation on visual field testing worse than -5dB better eye H: >25dB better ear | Physical functioning | No difference |
| Ogliari, 2021 | Multiple | High | Prospective cohort | Population | Older adults (50+) | 50,986 (3853) | No DSI | V/H: self-report single question Likert | Physical functioning | Worse |
| Olakunde, 2020 | USA, North America | High | Cross sectional | Population | Adults (18+) | 468,303 (1349) | No DSI | V/H: self report, single binary question | Sexual health | Same |
| Reed, 2020 | USA, North America | High | Cross sectional/ Retrospective cohort | Population | Adults (18+) | 10,748 (121) | No DSI | V/H: self-report, single question Likert | Health behaviours | Worse |
| Reuben, 1999 | USA, North America | High | Prospective cohort | Population | Older adults (55+) | 5,646 (36) | No SI | V: 6/12 better eye H: >40dB at 1 or/and 2kHz in better ear | Mortality | No difference |
| Shakarchi, 2021 | USA, North America | High | Prospective cohort | Population | Older adults (65+) | 7,648 (697) | No SI | V/H: self-report single question Likert scale | Walking speed | Worse |
| Soto-Perez-de-Celis, 2018 | USA, North America | High | Cross sectional | Clinic | Older adults (65+) | 750 (55) | No SI | V/H: self-report single categorical question | Physical functioning | No difference |
| Steinman, 2021 | USA, North America | High | Prospective cohort | Population | Older adults (65+) | 4,636 (111) | No DSI | V/H: self-report multiple questions | Mortality; self-rated health | Same |
| Sun, 2020 | China | Upper middle | Prospective cohort | Population | - | 37,076 (7,774) | No DSI | V/H: clinician judgement | Mortality | Worse |
| Tareque, 2019 | Singapore, Asia Pacific | High | Prospective cohort | Population | Older adults (60+) | 3,452 (401) | No SI | V/H: self-report single question with Likert scale | Mortality; Physical functioning | Worse |
| Teh, 2006 | Singapore, Asia Pacific | High | Retrospective cohort | Clinic | Older adults (50+) | 112 (36) | DSI | V: self-report single question binary H: Fail whisper voice test | Physical functioning | Varied |
| Tinetti, 1995 | USA, North America | High | Prospective cohort | Population | Older adults (70+) | 927 (11) | No SI | V: >50% impaired on VA chart (unclear) H: Fail whisper voice test (> 5 words missed) | Physical functioning | Worse |
| Wahl, 2013 | Germany, Western Europe | High | Cross sectional | Clinic | 75-94 | 430 (43) | No SI | V: <6/18 worse eye H: >=35dB PTA in better ear | General health | Worse |
| Williams, 2020 | Canada, North America | High | Retrospective cohort | Care home | Older adults (55+) | 371,696 (not stated) | No SI | V/H: self-report single categorical question | Physical functioning; long term care admissions | No difference |
| Yamada, 2014 | Multiple, Western Europe | High | Cross sectional | Care home | Older adults (50+) | 4,007 (1,275) | No SI | V/H: self-report single categorical question | General health | Worse |
| Yamada, 2016 | Multiple, Western Europe | High | Cross sectional | Care home | Older adults (50+) | 2,851 (88) | No SI | V/H: self-report single categorical question | Mortality | Worse |
| Zhang, 2020 | China, Asia Pacific | Upper middle | Prospective cohort | Population | Older adults (80+) | 8,788 (860) | No SI | V/H: self-report single categorical question | Mortality | Worse |
| Zhou, 2022 | China, Asia Pacific | Upper middle | Prospective cohort | Population | Older adults (45+) | 7,623 (3,163) | No DSI | V: self-report multiple questions  H: self-report single question Likert | Falls | Worse |

**S5 Table** : Included systematic reviews

| First author, year | Study design | Number of included studies | Outcome categories examined |
| --- | --- | --- | --- |
| Arcous, 2019 | Scoping review | 21 | Participation; psychosocial; physical |
| Ask Larsen, 2014 | Systematic review | 30 | Definitions |
| Besser, 2018 | Non-systematic review | 7 | Prevalence |
| Carvill, 2001 | Non-systematic review | Not reported | Psychosocial |
| Dammeyer, 2014 | Non-systematic review | Not reported | Psychosocial |
| Dewan, 2012 | Systematic review | 55 | Prevalence |
| Heine, 2015 | Systematic review | 42 | Participation; psychosocial |
| Heine, 2014 | Systematic review | 8 | Psychosocial |
| Jaiswal, 2018 | Scoping review | 54 | Participation |
| Lehane, 2017 | Integrative review | 24 | Psychosocial |
| Schneider, 2011 | Non-systematic review | 40 | Psychosocial; participation; physical |
| Simcock, 2017 | Systematic review | 28 | Psychosocial |
| Simcock, 2017 | Systematic review | 24 | Participation |
| Tseng, 2018 | Systematic review | 23 | Psychosocial |

**S6 Table: Included studies (n=197)**

|  | **Authors** | **Title** | **Year** | **Journal** |
| --- | --- | --- | --- | --- |
| 1 | Amini, Reza; Haghani, Hamid; Masoumi, Mehdi | Quality of life in the Iranian Blind War Survivors in 2007: a cross-sectional study. | 2010 | BMC international health and human rights |
| 2 | Appollonio, I; Carabellese, C; Magni, E; Frattola, L; Trabucchi, M | Sensory impairments and mortality in an elderly community population: a six-year follow-up study. | 1995 | Age and ageing |
| 3 | Arcous, Marine; Putois, Olivier; Dalle-Nazebi, Sophie; Kerbourch, Sylvain; Cariou, Anaelle; Ben Aissa, Ines; Marlin, Sandrine; Potier, Remy | Psychosocial determinants associated with quality of life in people with usher syndrome. A scoping review. | 2019 | Disability and rehabilitation |
| 4 | Armstrong T.W.; Surya S.; Elliott T.R.; Brossart D.F.; Burdine J.N. | Depression and health-related quality of life among persons with sensory disabilities in a health professional shortage area | 2016 | Rehabilitation Psychology |
| 5 | Armstrong, Nicole M; Wang, Hang; E, Jian-Yu; Lin, Frank R; Abraham, Alison G; Ramulu, Pradeep; Resnick, Susan M; Tian, Qu; Simonsick, Eleanor; Gross, Alden L; Schrack, Jennifer A; Ferrucci, Luigi; Agrawal, Yuri | Patterns of Prevalence of Multiple Sensory Impairments among Community-Dwelling Older Adults. | 2021 | The journals of gerontology. Series A, Biological sciences and medical sciences |
| 6 | Ask Larsen, Flemming; Damen, Saskia | Definitions of deafblindness and congenital deafblindness. | 2014 | Research in developmental disabilities |
| 7 | Assi, Lama; Ehrlich, Joshua R; Zhou, Yunshu; Huang, Alison; Kasper, Judith; Lin, Frank R; McKee, Michael M; Reed, Nicholas S; Swenor, Bonnielin K; Deal, Jennifer A | Self-reported dual sensory impairment, dementia, and functional limitations in Medicare beneficiaries. | 2021 | Journal of the American Geriatrics Society |
| 8 | Assi, Lama; Shakarchi, Ahmed F; Sheehan, Orla C; Deal, Jennifer A; Swenor, Bonnielin K; Reed, Nicholas S | Assessment of Sensory Impairment and Health Care Satisfaction Among Medicare Beneficiaries. | 2020 | JAMA network open |
| 9 | Beall, C M; Goldstein, M C | Age differences in sensory and cognitive function in elderly Nepalese. | 1986 | Journal of gerontology |
| 10 | Bergman, Birgitta; Rosenhall, Ulf | Vision and hearing in old age | 2001 | Scandinavian Audiology |
| 11 | Besser, Jana; Stropahl, Maren; Urry, Emily; Launer, Stefan | Comorbidities of hearing loss and the implications of multimorbidity for audiological care. | 2018 | Hearing research |
| 12 | Bodsworth, Sarah M; Clare, Isabel CH; Simblett, Sara K; Deafblind UK | Deafblindness and mental health: Psychological distress and unmet need among adults with dual sensory impairment | 2011 | British Journal of Visual Impairment |
| 13 | Bouscaren, N; Yildiz, H; Dartois, L; Vercambre, M N; Boutron-Ruault, M C | Decline in Instrumental Activities of Daily Living over 4-Year: The Association with Hearing, Visual and Dual Sensory Impairments among Non-Institutionalized Women. | 2019 | The journal of nutrition, health & aging |
| 14 | Brennan, Mark; Horowitz, Amy; Su, Ya-Ping | Dual sensory loss and its impact on everyday competence. | 2005 | The Gerontologist |
| 15 | Brennan, Mark; Su, Ya-ping; Horowitz, Amy | Longitudinal associations between dual sensory impairment and everyday competence among older adults. | 2006 | Journal of rehabilitation research and development |
| 16 | Bright, Tess; McCormick, Ian; Phiri, Mwanaisha; Mulwafu, Wakisa; Burton, Matthew; Polack, Sarah; Mactaggart, Islay; Yip, Jennifer L Y; Swanepoel, De Wet; Kuper, Hannah | Rationale and feasibility of a combined rapid assessment of avoidable blindness and hearing loss protocol. | 2020 | PloS one |
| 17 | Byeon, Gihwan; Oh, Gyu Han; Jhoo, Jin Hyeong; Jang, Jae-Won; Bae, Jong Bin; Han, Ji Won; et al. | Dual Sensory Impairment and Cognitive Impairment in the Korean Longitudinal Elderly Cohort. | 2021 | Neurology |
| 18 | Caban, Alberto J; Lee, David J; Gomez-Marin, Orlando; Lam, Byron L; Zheng, D Diane | Prevalence of concurrent hearing and visual impairment in US adults: The National Health Interview Survey, 1997-2002. | 2005 | American journal of public health |
| 19 | Cacchione, Pamela Z; Culp, Kennith; Dyck, Mary J; Laing, Joan | Risk for acute confusion in sensory-impaired, rural, long-term-care elders. | 2003 | Clinical nursing research |
| 20 | Campbell, V A; Crews, J E; Moriarty, D G; Zack, M M; Blackman, D K | Surveillance for sensory impairment, activity limitation, and health-related quality of life among older adults--United States, 1993-1997. | 1999 | MMWR. CDC surveillance summaries : Morbidity and mortality weekly report. CDC surveillance summaries |
| 21 | Capella-McDonnall, Michele E | The effects of single and dual sensory loss on symptoms of depression in the elderly. | 2005 | International journal of geriatric psychiatry |
| 22 | Carvill, S | Sensory impairments, intellectual disability and psychiatry. | 2001 | Journal of intellectual disability research : JIDR |
| 23 | Chia, Ee-Munn; Mitchell, Paul; Rochtchina, Elena; Foran, Suriya; Golding, Maryanne; Wang, Jie Jin | Association between vision and hearing impairments and their combined effects on quality of life. | 2006 | Archives of ophthalmology |
| 24 | Chou, Kee-Lee | Combined effect of vision and hearing impairment on depression in older adults: evidence from the English Longitudinal Study of Ageing. | 2008 | Journal of affective disorders |
| 25 | Chou, Kee-Lee; Chi, Iris | Combined effect of vision and hearing impairment on depression in elderly Chinese. | 2004 | International journal of geriatric psychiatry |
| 26 | Cimarolli, Verena R; Jopp, Daniela S | Sensory impairments and their associations with functional disability in a sample of the oldest-old. | 2014 | Quality of life research |
| 27 | Cimarolli, Verena R; Jopp, Daniela S; Boerner, Kathrin; Minahan, Jillian | Depressive symptoms in the oldest-old: The role of sensory impairments. | 2018 | Archives of gerontology and geriatrics |
| 28 | Clark, Michael S; Bond, Malcolm J; Sanchez, Linnett | The effect of sensory impairment on the lifestyle activities of older people | 1999 | Australasian Journal on Ageing |
| 29 | Cosh, S; von Hanno, T; Helmer, C; Bertelsen, G; Delcourt, C; Schirmer, H; SENSE-Cog Group | The association amongst visual, hearing, and dual sensory loss with depression and anxiety over 6 years: The Tromso Study. | 2018 | International journal of geriatric psychiatry |
| 30 | Crews, John E; Campbell, Vincent A | Vision impairment and hearing loss among community-dwelling older Americans: implications for health and functioning. | 2004 | American journal of public health |
| 31 | Crews, John E; Chou, Chiu-Fang; Sekar, Swathi; Saaddine, Jinan B | The Prevalence of Chronic Conditions and Poor Health Among People With and Without Vision Impairment, Aged >=65 Years, 2010-2014. | 2017 | American journal of ophthalmology |
| 32 | Crowe K, Hovaldt HB, Dammeyer J. | Communication participation in older adults with dual sensory loss. | 2018 | Speech Lang Hear. |
| 33 | Dalby, Dawn M; Hirdes, John P; Stolee, Paul; Strong, J Graham; Poss, Jeff; Tjam, Erin Y; Bowman, Lindsay; Ashworth, Melody | Characteristics of individuals with congenital and acquired deaf-blindness | 2009 | Journal of Visual Impairment & Blindness |
| 34 | Dammeyer, Jesper | Congenital rubella syndrome and delayed manifestations. | 2010 | International journal of pediatric otorhinolaryngology |
| 35 | Dammeyer, Jesper | Deafblindness: a review of the literature. | 2014 | Scandinavian journal of public health |
| 36 | Dammeyer, Jesper | Characteristics of a Danish population of adults with acquired deafblindness receiving rehabilitation services | 2013 | British Journal of Visual Impairment |
| 37 | Dammeyer, Jesper | Symptoms of autism among children with congenital deafblindness. | 2014 | Journal of autism and developmental disorders |
| 38 | Dammeyer, Jesper | Prevalence and aetiology of congenitally deafblind people in Denmark. | 2010 | International journal of audiology |
| 39 | Dammeyer, Jesper | Mental and behavioral disorders among people with congenital deafblindness. | 2011 | Research in developmental disabilities |
| 40 | Davidson, Jacob G S; Guthrie, Dawn M | Older Adults With a Combination of Vision and Hearing Impairment Experience Higher Rates of Cognitive Impairment, Functional Dependence, and Worse Outcomes Across a Set of Quality Indicators. | 2019 | Journal of aging and health |
| 41 | Dawes, Piers; Dickinson, Christine; Emsley, Richard; Bishop, Paul N; Cruickshanks, Karen J; Edmondson-Jones, Mark; et al | Vision impairment and dual sensory problems in middle age. | 2014 | Ophthalmic & physiological optics |
| 42 | de la Fuente, Javier; Hjelmborg, Jacob; Wod, Mette; de la Torre-Luque, Alejandro; Caballero, Francisco Felix; Christensen, Kaare; Ayuso-Mateos, Jose Luis | Longitudinal Associations of Sensory and Cognitive Functioning: A Structural Equation Modeling Approach. | 2019 | The journals of gerontology. Series B, Psychological sciences and social sciences |
| 43 | Deardorff, William J; Sloane, Richard J; Pavon, Juliessa M; Hastings, Susan N; Whitson, Heather E | Hospitalization Risk Among Older Adults with Sensory Impairments: Development of a Prognostic Model. | 2020 | Journal of the American Geriatrics Society |
| 44 | Deardorff, William James; Liu, Phillip L; Sloane, Richard; Van Houtven, Courtney; Pieper, Carl F; Hastings, Susan Nicole; Cohen, Harvey J; Whitson, Heather E | Association of Sensory and Cognitive Impairment With Healthcare Utilization and Cost in Older Adults. | 2019 | Journal of the American Geriatrics Society |
| 45 | Dewan, Pooja; Gupta, Piyush | Burden of Congenital Rubella Syndrome (CRS) in India: a systematic review. | 2012 | Indian pediatrics |
| 46 | Dupuis, Kate; Pichora-Fuller, M Kathleen; Chasteen, Alison L; Marchuk, Veronica; Singh, Gurjit; Smith, Sherri L | Effects of hearing and vision impairments on the Montreal Cognitive Assessment. | 2015 | Neuropsychology, development, and cognition. Section B, Aging, neuropsychology and cognition |
| 47 | Ehn, Mattias; Wahlqvist, Moa; Danermark, Berth; Dahlstrom, Orjan; Moller, Claes | Health, work, social trust, and financial situation in persons with Usher syndrome type 1. | 2018 | Work (Reading, Mass.) |
| 48 | Figueiredo, Marilia Zannon de Andrade; Chiari, Brasilia Maria; Goulart, Barbara Niegia Garcia de | Communication in deafblind adults with Usher syndrome: retrospective observational study. | 2013 | CoDAS |
| 49 | Fischer, Mary E; Cruickshanks, Karen J; Klein, Barbara E K; Klein, Ronald; Schubert, Carla R; Wiley, Terry L | Multiple sensory impairment and quality of life. | 2009 | Ophthalmic epidemiology |
| 50 | Fisher, Diana E; Ward, Michael M; Hoffman, Howard J; Li, Chuan-Ming; Cotch, Mary Frances | Impact of Sensory Impairments on Functional Disability in Adults With Arthritis. | 2016 | American journal of preventive medicine |
| 51 | Fisher, Diana; Li, Chuan-Ming; Chiu, May S; Themann, Christa L; Petersen, Hannes; Jonasson, Fribert; et al. | Impairments in hearing and vision impact on mortality in older people: the AGES-Reykjavik Study. | 2014 | Age and ageing |
| 52 | Fletcher, Paula C; Guthrie, Dawn M | The lived experiences of individuals with acquired deafblindness: Challenges and the future | 2013 | ?? |
| 53 | Forbes W.F.; Hayward L.M.; Agwani N. | Factors associated with the prevalence of various self-reported impairments among older people residing in the community | 1991 | Canadian Journal of Public Health |
| 54 | Fuller, Spencer D; Mudie, Lucy I; Siordia, Carlos; Swenor, Bonnielin K; Friedman, David S | Nationwide Prevalence of Self-Reported Serious Sensory Impairments and Their Associations with Self-Reported Cognitive and Functional Difficulties. | 2018 | Ophthalmology |
| 55 | Gadkaree, S. K.; Sun, D. Q.; Li, C.; Lin, F. R.; Ferrucci, L.; Simonsick, E. M.; Agrawal, Y. | Does Sensory Function Decline Independently or Concomitantly with Age? Data from the Baltimore Longitudinal Study of Aging | 2016 | Journal of aging research |
| 56 | Ge, Shaoqing; McConnell, Eleanor S; Wu, Bei; Pan, Wei; Dong, XinQi; Plassman, Brenda L | Longitudinal Association Between Hearing Loss, Vision Loss, Dual Sensory Loss, and Cognitive Decline. | 2021 | Journal of the American Geriatrics Society |
| 57 | Glatz, Marlene; Riedl, Regina; Glatz, Wilfried; Schneider, Mona; Wedrich, Andreas; Bolz, Matthias; Strauss, Rupert W | Blindness and visual impairment in Central Europe. | 2022 | PloS one |
| 58 | Gopinath, B; Schneider, J; Flood, V M; McMahon, C M; Burlutsky, G; Leeder, S R; Mitchell, P | Association between diet quality with concurrent vision and hearing impairment in older adults. | 2014 | The journal of nutrition, health & aging |
| 59 | Gopinath, Bamini; Liew, Gerald; Burlutsky, George; McMahon, Catherine M; Mitchell, Paul | Association between vision and hearing impairment and successful aging over five years. | 2021 | Maturitas |
| 60 | Gopinath, Bamini; Liew, Gerald; Burlutsky, George; McMahon, Catherine M; Mitchell, Paul | Visual and hearing impairment and retirement in older adults: A population-based cohort study. | 2017 | Maturitas |
| 61 | Gopinath, Bamini; McMahon, Catherine M; Burlutsky, George; Mitchell, Paul | Hearing and vision impairment and the 5-year incidence of falls in older adults. | 2016 | Age and ageing |
| 62 | Gopinath, Bamini; Schneider, Julie; McMahon, Catherine M; Burlutsky, George; Leeder, Stephen R; Mitchell, Paul | Dual sensory impairment in older adults increases the risk of mortality: a population-based study. | 2013 | PloS one |
| 63 | Graue-Hernandez, Enrique O; Gomez-Dantes, Hector; Romero-Martinez, Martin; Bravo, Gerardo; Arrieta-Camacho, Jesus; Jimenez-Corona, Aida | [Self-reported hearing loss and visual impairment in adults from Central Mexico]. | 2019 | Salud publica de Mexico |
| 64 | Green, Kimberly A; McGwin, Gerald Jr; Owsley, Cynthia | Associations between visual, hearing, and dual sensory impairments and history of motor vehicle collision involvement of older drivers. | 2013 | Journal of the American Geriatrics Society |
| 65 | Grue, Else Vengnes; Kirkevold, Marit; Ranhoff, Anette Hylen | Prevalence of vision, hearing, and combined vision and hearing impairments in patients with hip fractures. | 2009 | Journal of clinical nursing |
| 66 | Grue, Else Vengnes; Ranhoff, Anette Hylen; Noro, Anja; Finne-Soveri, Harriet; Jensdottir, Anna Birna; Ljunggren, Gunnar; Bucht, Gosta; Bjornson, Leif Jan; Jonsen, Elisabeth; Schroll, Marianne; Jonsson, Palmi V | Vision and hearing impairments and their associations with falling and loss of instrumental activities in daily living in acute hospitalized older persons in five Nordic hospitals. | 2009 | Scandinavian journal of caring sciences |
| 67 | Guthrie, Dawn M; Davidson, Jacob G S; Williams, Nicole; Campos, Jennifer; Hunter, Kathleen; Mick, Paul; Orange, Joseph B; Pichora-Fuller, M Kathleen; Phillips, Natalie A; Savundranayagam, Marie Y; Wittich, Walter | Combined impairments in vision, hearing and cognition are associated with greater levels of functional and communication difficulties than cognitive impairment alone: Analysis of interRAI data for home care and long-term care recipients in Ontario. | 2018 | PloS one |
| 68 | Guthrie, Dawn M; Declercq, Anja; Finne-Soveri, Harriet; Fries, Brant E; Hirdes, John P | The Health and Well-Being of Older Adults with Dual Sensory Impairment (DSI) in Four Countries. | 2016 | PloS one |
| 69 | Guthrie, Dawn M; ThÃ©riault, Ã‰ric R; Davidson, Jacob GS | Self-rated health, cognition, and dual sensory impairment are important predictors of depression among home care clients in Ontario | 2016 | Home Health Care Management & Practice |
| 70 | Haanes, Gro Gade; Kirkevold, Marit; Horgen, Gunnar; Hofoss, Dag; Eilertsen, Grethe | Sensory impairments in community health care: a descriptive study of hearing and vision among elderly Norwegians living at home. | 2014 | Journal of multidisciplinary healthcare |
| 71 | Haanes, Gro Gade; Roin, Asa; Petersen, Maria Skaalum | Preventive Home Visit (PHV) Screening of Hearing and Vision Among Older Adults in Torshavn, Faroe Islands: A Feasibility Study in a Small-Scale Community. | 2021 | Journal of multidisciplinary healthcare |
| 72 | Hajek, Andre; Konig, Hans-Helmut | Dual sensory impairment and psychosocial factors. Findings based on a nationally representative sample. | 2020 | Archives of gerontology and geriatrics |
| 73 | Han, J H; Lee, H J; Jung, J; Park, E-C | Effects of self-reported hearing or vision impairment on depressive symptoms: a population-based longitudinal study. | 2019 | Epidemiology and psychiatric sciences |
| 74 | Harada, Sei; Nishiwaki, Yuji; Michikawa, Takehiro; Kikuchi, Yuriko; Iwasawa, Satoko; Nakano, Makiko; Ishigami, Ai; Saito, Hideyuki; Takebayashi, Toru | Gender difference in the relationships between vision and hearing impairments and negative well-being. | 2008 | Preventive medicine |
| 75 | Harithasan, Deepashini; Mukari, Siti Zamratol-Mai Sarah; Ishak, Wan Syafira; Shahar, Suzana; Yeong, Wong Lai | The impact of sensory impairment on cognitive performance, quality of life, depression, and loneliness in older adults. | 2020 | International journal of geriatric psychiatry |
| 76 | Hartshorne, Timothy S; Heussler, Helen S; Dailor, A Nichole; Williams, George L; Papadopoulos, Dimitrios; Brandt, Kimberly K | Sleep disturbances in CHARGE syndrome: types and relationships with behavior and caregiver well-being. | 2009 | Developmental medicine and child neurology |
| 77 | Hartshorne, Timothy S; Nicholas, Jude; Grialou, Tina L; Russ, Joanna M | Executive function in CHARGE syndrome | 2007 | Child Neuropsychology |
| 78 | Heine, Chyrisse; Browning, Colette | Dual Sensory Loss in Older Adults: A Systematic Review. | 2015 | The Gerontologist |
| 79 | Heine, Chyrisse; Browning, Colette J | Mental health and dual sensory loss in older adults: a systematic review. | 2014 | Frontiers in aging neuroscience |
| 80 | Heine, Chyrisse; Browning, Colette J; Gong, Cathy Honge | Sensory Loss in China: Prevalence, Use of Aids, and Impacts on Social Participation. | 2019 | Frontiers in public health |
| 81 | Heine, Chyrisse; Gong, Cathy Honge; Browning, Colette | Dual Sensory Loss, Mental Health, and Wellbeing of Older Adults Living in China. | 2019 | Frontiers in public health |
| 82 | Heine, Chyrisse; Gong, Cathy Honge; Feldman, Susan; Browning, Colette | Older Women in Australia: Facing the Challenges of Dual Sensory Loss. | 2019 | International journal of environmental research and public health |
| 83 | Hersh M.A. | Deafblind people, stigma and the use of communication and mobility assistive devices | 2013 | Technology and Disability |
| 84 | Heyl, Vera; Wahl, Hans-Werner | Managing daily life with age-related sensory loss: cognitive resources gain in importance. | 2012 | Psychology and aging |
| 85 | Hickson, Louise; Lind, Christopher; Worrall, Linda; Yiu, Edwin; Barnett, Heather; Lovie-Kitchin, Jan | Hearing and vision in healthy older Australians: Objective and self-report measures | 1999 | Advances in Speech Language Pathology |
| 86 | Ho, Kam Chun; Fenwick, Eva K; Gupta, Preeti; Gan, Alfred; Loo, Jenny Hy; Ma, Lina; Koh, Gerald; Wong, Tien Y; Lamoureux, Ecosse L; Man, Ryan Ek | Prevalence, Associated Factors and Health-related Quality of Life of Dual Sensory Impairment in Residential Care Facilities in Singapore. | 2021 | Ophthalmic epidemiology |
| 87 | Hong, Thomas; Mitchell, Paul; Burlutsky, George; Liew, Gerald; Wang, Jie Jin | Visual Impairment, Hearing Loss and Cognitive Function in an Older Population: Longitudinal Findings from the Blue Mountains Eye Study. | 2016 | PloS one |
| 88 | Hovaldt HB, Lund R, Lehane CM, Dammeyer J | Relational strain in close social relations among older adults with dual sensory loss | 2019 | Br J Vis Impair. |
| 89 | Hovaldt, Hanna B; Crowe, Kathryn; Dammeyer, Jesper | A cross-sectional study of prevalence and correlates of self-harm and suicidal ideation in older adults with dual sensory loss. | 2022 | Disability and health journal |
| 90 | Huddle, Matthew G; Deal, Jennifer A; Swenor, Bonnielin; Genther, Dane J; Lin, Frank R | The Association of Dual Sensory Impairment with Hospitalization and Burden of Disease | 2016 | Journal of the American Geriatrics Society |
| 91 | Hwang, Phillip H; Longstreth, W T Jr; Brenowitz, Willa D; Thielke, Stephen M; Lopez, Oscar L; Francis, Courtney E; DeKosky, Steven T; Fitzpatrick, Annette L | Dual sensory impairment in older adults and risk of dementia from the GEM Study. | 2020 | Alzheimer's & dementia (Amsterdam, Netherlands) |
| 92 | Jaiswal, Atul; Aldersey, Heather M; Wittich, Walter; Mirza, Mansha; Finlayson, Marcia | Meaning and experiences of participation: a phenomenological study with persons with deafblindness in India. | 2019 | Disability and rehabilitation |
| 93 | Jaiswal, Atul; Aldersey, Heather; Wittich, Walter; Mirza, Mansha; Finlayson, Marcia | Participation experiences of people with deafblindness or dual sensory loss: A scoping review of global deafblind literature. | 2018 | PloS one |
| 94 | Jin, Elvine Y W; Daly, Blanaid | The self-reported oral health status and behaviors of adults who are deaf and blind. | 2010 | Special care in dentistry |
| 95 | Jung, Younhea; Han, Kyungdo; Lee, Ji Min; Park, Hye Yeon; Moon, Jung Il | Impact of vision and hearing impairments on risk of cardiovascular outcomes and mortality in patients with type 2 diabetes: A nationwide cohort study. | 2022 | Journal of diabetes investigation |
| 96 | Keller, B K; Morton, J L; Thomas, V S; Potter, J F | The effect of visual and hearing impairments on functional status. | 1999 | Journal of the American Geriatrics Society |
| 97 | Khandekar, Rajiv; Al Khabori, Mazin | Double disability: the hearing-impaired blind in the Sultanate of Oman. | 2004 | International journal of audiology |
| 98 | Khil, Laura; Wellmann, Jurgen; Berger, Klaus | Impact of combined sensory impairments on health-related quality of life. | 2015 | Quality of life research |
| 99 | Khurana, Maitri; Shoham, Natalie; Cooper, Claudia; Pitman, Alexandra Laura | Association between sensory impairment and suicidal ideation and attempt: a cross-sectional analysis of nationally representative English household data. | 2021 | BMJ open |
| 100 | Kiely, Kim M; Anstey, Kaarin J; Luszcz, Mary A | Dual sensory loss and depressive symptoms: the importance of hearing, daily functioning, and activity engagement. | 2013 | Frontiers in human neuroscience |
| 101 | Kiely, Kim M; Mitchell, Paul; Gopinath, Bamini; Luszcz, Mary A; Jagger, Carol; Anstey, Kaarin J | Estimating the Years Lived With and Without Age-Related Sensory Impairment. | 2016 | The journals of gerontology. Series A, Biological sciences and medical sciences |
| 102 | Kiely, Kim M; Mortby, Moyra E; Anstey, Kaarin J | Differential associations between sensory loss and neuropsychiatric symptoms in adults with and without a neurocognitive disorder. | 2018 | International psychogeriatrics |
| 103 | Killeen O.J.; Xiang X.; Powell D.; Reed N.S.; Deal J.A.; Swenor B.K.; Ehrlich J.R. | Longitudinal Associations of Self-Reported Visual, Hearing, and Dual Sensory Difficulties With Symptoms of Depression Among Older Adults in the United States | 2022 | Frontiers in Neuroscience |
| 104 | Kim, Yoonjung; Kwak, Yeunhee; Kim, Ji-Su | The association between suicide ideation and sensory impairment among elderly Koreans. | 2015 | Aging & mental health |
| 105 | Klein, R; Cruickshanks, K J; Klein, B E; Nondahl, D M; Wiley, T | Is age-related maculopathy related to hearing loss?. | 1998 | Archives of ophthalmology |
| 106 | Kulmala, Jenni; Viljanen, Anne; Sipila, Sarianna; Pajala, Satu; Parssinen, Olavi; Kauppinen, Markku; Koskenvuo, Markku; Kaprio, Jaakko; Rantanen, Taina | Poor vision accompanied with other sensory impairments as a predictor of falls in older women. | 2009 | Age and ageing |
| 107 | Kuo, Pei-Lun; Huang, Alison R; Ehrlich, Joshua R; Kasper, Judith; Lin, Frank R; McKee, Michael M; Reed, Nicholas S; Swenor, Bonnielin K; Deal, Jennifer A | Prevalence of Concurrent Functional Vision and Hearing Impairment and Association With Dementia in Community-Dwelling Medicare Beneficiaries. | 2021 | JAMA network open |
| 108 | Kwan, Rick Yiu Cho; Kwan, Chi Wai; Kor, Patrick Pui Kin; Chi, Iris | Cognitive decline, sensory impairment, and the use of audio-visual aids by long-term care facility residents. | 2022 | BMC geriatrics |
| 109 | Kwon, Hye-Jin; Kim, Ji-Su; Kim, Yoon-Jung; Kwon, Su-Jin; Yu, Jin-Na | Sensory Impairment and Health-Related Quality of Life. | 2015 | Iranian journal of public health |
| 110 | Lach, Helen W; Lozano, Alicia J; Hanlon, Alexandra L; Cacchione, Pamela Z | Fear of falling in sensory impaired nursing home residents. | 2020 | Aging & mental health |
| 111 | Lam, Byron L; Lee, David J; Gomez-Marin, Orlando; Zheng, D Diane; Caban, Alberto J | Concurrent visual and hearing impairment and risk of mortality: the National Health Interview Survey. | 2006 | Archives of ophthalmology |
| 112 | Lee, David J; Gomez-Marin, Orlando; Lam, Byron L; Zheng, D Diane; Arheart, Kristopher L; Christ, Sharon L; Caban, Alberto J | Severity of concurrent visual and hearing impairment and mortality: the 1986-1994 National Health Interview Survey. | 2007 | Journal of aging and health |
| 113 | Lee, David J; Lam, Byron L; Gomez-Marin, Orlando; Zheng, D Diane; Caban, Alberto J | Concurrent hearing and visual impairment and morbidity in community-residing adults: the National Health Interview Survey, 1986 to 1996. | 2005 | Journal of aging and health |
| 114 | Lehane C.M.; Dammeyer J.; Wittich W. | Intra- and interpersonal effects of coping on the psychological well-being of adults with sensory loss and their spouses | 2019 | Disability and rehabilitation |
| 115 | Lehane, Christine M; Dammeyer, Jesper; Elsass, Peter | Sensory loss and its consequences for couples' psychosocial and relational wellbeing: an integrative review. | 2017 | Aging & mental health |
| 116 | Liljas, Ann E M; Walters, Kate; de Oliveira, Cesar; Wannamethee, S Goya; Ramsay, Sheena E; Carvalho, Livia A | Self-Reported Sensory Impairments and Changes in Cognitive Performance: A Longitudinal 6-Year Follow-Up Study of English Community-Dwelling Adults Aged 50 Years. | 2018 | Journal of aging and health |
| 117 | Lin, Michael Y; Gutierrez, Peter R; Stone, Katie L; Yaffe, Kristine; Ensrud, Kristine E; Fink, Howard A; et al. | Vision impairment and combined vision and hearing impairment predict cognitive and functional decline in older women. | 2004 | Journal of the American Geriatrics Society |
| 118 | Linden-Bostrom, Margareta; Persson, Carina | Disparities in mental health among adolescents with and without impairments. | 2015 | Scandinavian journal of public health |
| 119 | Liu P.L.; Cohen H.J.; Fillenbaum G.G.; Burchett B.M.; Whitson H.E. | Association of Co-Existing Impairments in Cognition and Self-Rated Vision and Hearing With Health Outcomes in Older Adults | 2016 | Gerontology and Geriatric Medicine |
| 120 | Liu, Wenwen; Yang, Chao; Liu, Lili; Kong, Guilan; Zhang, Luxia | Bidirectional associations of vision loss, hearing loss, and dual sensory loss with depressive symptoms among the middle-aged and older adults in China. | 2022 | Journal of affective disorders |
| 121 | Loprinzi P.D.; Smit E.; Pariser G. | Association among depression, physical functioning, and hearing and vision impairment in adults with diabetes | 2013 | Diabetes Spectrum |
| 122 | Loprinzi, Paul D; Smit, Ellen; Lin, Frank R; Gilham, Ben; Ramulu, Pradeep Y | Accelerometer-assessed physical activity and objectively determined dual sensory impairment in US adults. | 2013 | Mayo Clinic proceedings |
| 123 | Lundin, Elin; Widen, Stephen E; Wahlqvist, Moa; Anderzen-Carlsson, Agneta; Granberg, Sarah | Prevalence, diagnoses and rehabilitation services related to severe dual sensory loss (DSL) in older persons: a cross-sectional study based on medical records. | 2020 | International journal of audiology |
| 124 | Luo, Yanan; He, Ping; Guo, Chao; Chen, Gong; Li, Ning; Zheng, Xiaoying | Association Between Sensory Impairment and Dementia in Older Adults: Evidence from China. | 2018 | Journal of the American Geriatrics Society |
| 125 | Lupsakko, Taina; Mantyjarvi, Maija; Kautiainen, Hannu; Sulkava, Raimo | Combined hearing and visual impairment and depression in a population aged 75 years and older. | 2002 | International journal of geriatric psychiatry |
| 126 | Lyu, Jiyoung; Kim, Hae-Young | Gender-Specific Associations of Sensory Impairments with Depression and Cognitive Impairment in Later Life. | 2018 | Psychiatry investigation |
| 127 | Ma X.; Wei J.; Congdon N.; Li Y.; Shi L.; Zhang D. | Longitudinal Association Between Self-Reported Sensory Impairments and Episodic Memory among Older Adults in China: A Prospective Cohort Study | 2021 | Journal of Geriatric Psychiatry and Neurology |
| 128 | Mactaggart, Islay; Polack, Sarah; Murthy, Gvs; Kuper, Hannah | A population-based survey of visual impairment and its correlates in Mahabubnagar district, Telangana State, India. | 2018 | Ophthalmic epidemiology |
| 129 | Mah, Ho Y; Ishak, Wan S; Abd Rahman, Mohd H | Prevalence and risk factors of dual sensory impairment among community-dwelling older adults in Selangor: A secondary data analysis. | 2020 | Geriatrics & gerontology international |
| 130 | Maharani, Asri; Dawes, Piers; Nazroo, James; Tampubolon, Gindo; Pendleton, Neil; Sense-Cog WP1 Group | Associations Between Self-Reported Sensory Impairment and Risk of Cognitive Decline and Impairment in the Health and Retirement Study Cohort. | 2020 | The journals of gerontology. Series B, Psychological sciences and social sciences |
| 131 | Maharani, Asri; Dawes, Piers; Nazroo, James; Tampubolon, Gindo; Pendleton, Neil; Sense-Cog WP1 group | Visual and hearing impairments are associated with cognitive decline in older people. | 2018 | Age and ageing |
| 132 | Marmamula, Srinivas; Kumbham, Thirupathi Reddy; Modepalli, Satya Brahmanandam; Barrenkala, Navya Rekha; Yellapragada, Ratnakar; Shidhaye, Rahul | Depression, combined visual and hearing impairment (dual sensory impairment): a hidden multi-morbidity among the elderly in Residential Care in India. | 2021 | Scientific reports |
| 133 | Maruta, Michio; Tabira, Takayuki; Sagari, Akira; Miyata, Hironori; Yoshimitsu, Koji; Han, Gwanghee; Yoshiura, Kazuhiro; Matsuo, Takashi; Kawagoe, Masahiro | Impact of sensory impairments on dementia incidence and symptoms among Japanese older adults. | 2020 | Psychogeriatrics |
| 134 | Maruta, Michio; Tabira, Takayuki; Sagari, Akira; Miyata, Hironori; Yoshimitsu, Koji; Han, Gwanghee; Yoshiura, Kazuhiro; Matsuo, Takashi; Kawagoe, Masahiro | Impact of sensory impairments on dementia incidence and symptoms among Japanese older adults. | 2019 | Psychogeriatrics |
| 135 | McDonnall, Michele Capella | Physical status as a moderator of depressive symptoms among older adults with dual sensory loss. | 2011 | Rehabilitation psychology |
| 136 | McDonnall, Michele Capella | The effects of developing a dual sensory loss on depression in older adults: a longitudinal study. | 2009 | Journal of aging and health |
| 137 | McDonnall, Michele Capella | The Effect of Productive Activities on Depressive Symptoms Among Older Adults With Dual Sensory Loss. | 2011 | Research on aging |
| 138 | Meuwese-Jongejeugd, Anneke; van Splunder, Jacques; Vink, Marianne; Stilma, Jan Sietse; van Zanten, Bert; Verschuure, Hans; Bernsen, Roos; Evenhuis, Heleen | Combined sensory impairment (deaf-blindness) in five percent of adults with intellectual disabilities. | 2008 | American journal of mental retardation |
| 139 | Michalowsky, Bernhard; Hoffmann, Wolfgang; Kostev, Karel | Association Between Hearing and Vision Impairment and Risk of Dementia: Results of a Case-Control Study Based on Secondary Data. | 2019 | Frontiers in aging neuroscience |
| 140 | Mick, Paul Thomas; Hamalainen, Anni; Kolisang, Lebo; Pichora-Fuller, M Kathleen; Phillips, Natalie; Guthrie, Dawn; Wittich, Walter | The Prevalence of Hearing, Vision, and Dual Sensory Loss in Older Canadians: An Analysis of Data from the Canadian Longitudinal Study on Aging. | 2021 | Canadian journal on aging |
| 141 | Mick, Paul; Parfyonov, Maksim; Wittich, Walter; Phillips, Natalie; Guthrie, Dawn; Kathleen Pichora-Fuller, M | Associations between sensory loss and social networks, participation, support, and loneliness: Analysis of the Canadian Longitudinal Study on Aging. | 2018 | Canadian family physician |
| 142 | Mitoku, Kazuko; Masaki, Naoko; Ogata, Yukiko; Okamoto, Kazushi | Vision and hearing impairments, cognitive impairment and mortality among long-term care recipients: a population-based cohort study. | 2016 | BMC geriatrics |
| 143 | Miyawaki, Atsushi; Kobayashi, Yasuki; Kawachi, Ichiro | Self-Reported Hearing/Visual Loss and Mortality in Middle-Aged and Older Adults: Findings From the Komo-Ise Cohort, Japan. | 2020 | Journal of epidemiology |
| 144 | Moller, Kerstin | Deafblindness: a challenge for assessment--is the ICF a useful tool?. | 2003 | International journal of audiology |
| 145 | Morandi, Alessandro; Inzitari, Marco; Udina, Cristina; Gual, Neus; Mota, Miriam; Tassistro, Elena; et al. | Visual and Hearing Impairment Are Associated With Delirium in Hospitalized Patients: Results of a Multisite Prevalence Study. | 2021 | Journal of the American Medical Directors Association |
| 146 | Mudie, Lucy I; Varadaraj, Varshini; Gajwani, Prateek; Munoz, Beatriz; Ramulu, Pradeep; Lin, Frank R; Swenor, Bonnielin K; Friedman, David S; Zebardast, Nazlee | Dual sensory impairment: The association between glaucomatous vision loss and hearing impairment and function. | 2018 | PloS one |
| 147 | Mueller-Schotte, Sigrid; Zuithoff, Nicolaas P A; van der Schouw, Yvonne T; Schuurmans, Marieke J; Bleijenberg, Nienke | Trajectories of Limitations in Instrumental Activities of Daily Living in Frail Older Adults With Vision, Hearing, or Dual Sensory Loss. | 2019 | The journals of gerontology. Series A, Biological sciences and medical sciences |
| 148 | Ogliari, Giulia; Ryg, Jesper; Qureshi, Nadeem; Andersen-Ranberg, Karen; Scheel-Hincke, Lasse Lybecker; Masud, Tahir | Subjective vision and hearing impairment and falls among community-dwelling adults: a prospective study in the Survey of Health, Ageing and Retirement in Europe (SHARE). | 2021 | European geriatric medicine |
| 149 | Olakunde, Babayemi O; Pharr, Jennifer R | HIV-related risk behaviors and HIV testing among people with sensory disabilities in the United States. | 2020 | International journal of STD & AIDS |
| 150 | Pabst, Alexander; Bar, Jonathan; Rohr, Susanne; Lobner, Margrit; Kleineidam, Luca; Heser, Kathrin; et al. | Do self-reported hearing and visual impairments predict longitudinal dementia in older adults?. | 2021 | Journal of the American Geriatrics Society |
| 151 | Parada, Humberto; Laughlin, Gail A; Yang, Mingan; Nedjat-Haiem, Frances R; McEvoy, Linda K | Dual impairments in visual and hearing acuity and age-related cognitive decline in older adults from the Rancho Bernardo Study of Healthy Aging. | 2021 | Age and ageing |
| 152 | Pardhan, Shahina; Lopez Sanchez, Guillermo F; Bourne, Rupert; Davis, Adrian; Leveziel, Nicolas; Koyanagi, Ai; Smith, Lee | Visual, hearing, and dual sensory impairment are associated with higher depression and anxiety in women. | 2021 | International journal of geriatric psychiatry |
| 153 | Pardhan, Shahina; Smith, Lee; Bourne, Rupert; Davis, Adrian; Leveziel, Nicolas; Jacob, Louis; Koyanagi, Ai; Lopez-Sanchez, Guillermo F | Combined Vision and Hearing Difficulties Results in Higher Levels of Depression and Chronic Anxiety: Data From a Large Sample of Spanish Adults. | 2020 | Frontiers in psychology |
| 154 | Petrovsky, Darina V; Sefcik, Justine S; Hanlon, Alexandra L; Lozano, Alicia J; Cacchione, Pamela Z | Social Engagement, Cognition, Depression, and Comorbidity in Nursing Home Residents With Sensory Impairment. | 2019 | Research in gerontological nursing |
| 155 | Phua, June; Visaria, Abhijit; Ostbye, Truls; Malhotra, Rahul | Association of vision and hearing impairments with quality of life among older adults: Mediation by psychosocial factors. | 2022 | Geriatrics & gerontology international |
| 156 | R, Deepthi; Kasthuri, Arvind | Visual and hearing impairment among rural elderly of south India: a community-based study. | 2012 | Geriatrics & gerontology international |
| 157 | Raina P.; Wong M.; Massfeller H. | The relationship between sensory impairment and functional independence among elderly | 2004 | BMC Geriatrics |
| 158 | Ramamurthy D, Kasthuri A, Sonavane R | Dual sensory impairment among community dwelling rural elderly: concern for rehabilitation | 2014 | J Geriatr |
| 159 | Reed, Nicholas S; Assi, Lama; Pedersen, Emily; Alshabasy, Yasmeen; Deemer, Ashley; Deal, Jennifer A; Willink, Amber; Swenor, Bonnielin K | Accompaniment to healthcare visits: the impact of sensory impairment. | 2020 | BMC health services research |
| 160 | Reuben, D B; Mui, S; Damesyn, M; Moore, A A; Greendale, G A | The prognostic value of sensory impairment in older persons. | 1999 | Journal of the American Geriatrics Society |
| 161 | Roets-Merken, Lieve; Zuidema, Sytse; Vernooij-Dassen, Myrra; Dees, Marianne; Hermsen, Pieter; Kempen, Gertrudis; Graff, Maud | Problems identified by dual sensory impaired older adults in long-term care when using a self-management program: A qualitative study. | 2017 | PloS one |
| 162 | Rong, Hongguo; Lai, Xiaozhen; Jing, Rize; Wang, Xiao; Fang, Hai; Mahmoudi, Elham | Association of Sensory Impairments With Cognitive Decline and Depression Among Older Adults in China. | 2020 | JAMA network open |
| 163 | Schneck, Marilyn E; Lott, Lori A; Haegerstrom-Portnoy, Gunilla; Brabyn, John A | Association between hearing and vision impairments in older adults. | 2012 | Ophthalmic & physiological optics |
| 164 | Schneider, Julie M; Gopinath, Bamini; McMahon, Catherine M; Leeder, Stephen R; Mitchell, Paul; Wang, Jie Jin | Dual sensory impairment in older age. | 2011 | Journal of aging and health |
| 165 | Schneider, Julie; Gopinath, Bamini; McMahon, Catherine; Teber, Erdahl; Leeder, Stephen R; Wang, Jie Jin; Mitchell, Paul | Prevalence and 5-year incidence of dual sensory impairment in an older Australian population. | 2012 | Annals of epidemiology |
| 166 | Shakarchi, Ahmed F; Assi, Lama; Ehrlich, Joshua R; Deal, Jennifer A; Reed, Nicholas S; Swenor, Bonnielin K | Dual Sensory Impairment and Perceived Everyday Discrimination in the United States. | 2020 | JAMA ophthalmology |
| 167 | Shakarchi, Ahmed F; Assi, Lama; Gami, Abhishek; Kohn, Christina; Ehrlich, Joshua R; Swenor, Bonnielin K; Reed, Nicholas S | The Association of Vision, Hearing, and Dual-Sensory Loss with Walking Speed and Incident Slow Walking: Longitudinal and Time to Event Analyses in the Health and Retirement Study. | 2021 | Seminars in hearing |
| 168 | Simcock, Peter | Ageing with a unique impairment: a systematically conducted review of older deafblind people's experiences | 2017 | Ageing & Society |
| 169 | Simcock, Peter | One of society's most vulnerable groups? A systematically conducted literature review exploring the vulnerability of deafblind people. | 2017 | Health & social care in the community |
| 170 | Simning, Adam; Fox, Meghan L; Barnett, Steven L; Sorensen, Silvia; Conwell, Yeates | Depressive and Anxiety Symptoms in Older Adults With Auditory, Vision, and Dual Sensory Impairment. | 2019 | Journal of aging and health |
| 171 | Smith, Sherri L; Bennett, Loren W; Wilson, Richard H | Prevalence and characteristics of dual sensory impairment (hearing and vision) in a veteran population. | 2008 | Journal of rehabilitation research and development |
| 172 | Soto-Perez-de-Celis, Enrique; Sun, Can-Lan; Tew, William P; Mohile, Supriya Gupta; Gajra, Ajeet; et al. | Association between patient-reported hearing and visual impairments and functional, psychological, and cognitive status among older adults with cancer. | 2018 | Cancer |
| 173 | Steinman, Bernard A; Tabler, Jennifer; Mittlieder, Casandra M; Whitlock, Bremen; Goodman, Carrie E | Self-Reported Sensory Impairments in Older Adults and their Association with Self-Rated Health and Mortality Outcomes. | 2021 | Journal of aging and health |
| 174 | Sun, Ji; Li, Lin; Sun, Jiangwei | Sensory impairment and all-cause mortality among the elderly adults in China: a population-based cohort study. | 2020 | Aging |
| 175 | Swenor, Bonnielin K; Ramulu, Pradeep Y; Willis, Jeffery R; Friedman, David; Lin, Frank R | The prevalence of concurrent hearing and vision impairment in the United States | 2013 | JAMA internal medicine |
| 176 | Tan, Benjamin Kye Jyn; Ng, Faye Yu Ci; Song, Harris Jun Jie Muhammad Danial; Tan, Nicole Kye Wen; Ng, Li Shia; Loh, Woei Shyang | Associations of Hearing Loss and Dual Sensory Loss With Mortality: A Systematic Review, Meta-analysis, and Meta-regression of 26 Observational Studies With 1213756 Participants. | 2022 | JAMA otolaryngology |
| 177 | Tareque, Md Ismail; Chan, Angelique; Saito, Yasuhiko; Ma, Stefan; Malhotra, Rahul | The Impact of Self-Reported Vision and Hearing Impairment on Health Expectancy. | 2019 | Journal of the American Geriatrics Society |
| 178 | Teh ChuAi [Teh, C. A. R.]; Lim WeeShiong; Basri, R.; Ismail, N. H. | Utility of a patient-response screening question for visual impairment. | 2006 | Journal of the American Geriatrics Society |
| 179 | Tinetti, M E; Inouye, S K; Gill, T M; Doucette, J T | Shared risk factors for falls, incontinence, and functional dependence. Unifying the approach to geriatric syndromes. | 1995 | JAMA |
| 180 | Tseng, Ya-Chuan; Liu, Sara Hsin-Yi; Lou, Meei-Fang; Huang, Guey-Shiun | Quality of life in older adults with sensory impairments: a systematic review. | 2018 | Quality of life research |
| 181 | Turunen-Taheri, Satu; Skagerstrand, Asa; Hellstrom, Sten; Carlsson, Per-Inge | Patients with severe-to-profound hearing impairment and simultaneous severe vision impairment: a quality-of-life study. | 2017 | Acta oto-laryngologica |
| 182 | Urqueta Alfaro, Andrea; Guthrie, Dawn M; McGraw, Cathy; Wittich, Walter | Older adults with dual sensory loss in rehabilitation show high functioning and may fare better than those with single sensory loss. | 2020 | PloS one |
| 183 | Viljanen, A.; Kulmala, J.; Rantakokko, M.; Koskenvuo, M.; Kaprio, J.; Rantanen, T. | Accumulation of sensory difficulties predicts fear of falling in older women. | 2013 | Journal of Aging and Health |
| 184 | Viljanen, Anne; Tormakangas, Timo; Vestergaard, Sonja; Andersen-Ranberg, Karen | Dual sensory loss and social participation in older Europeans. | 2014 | European journal of ageing |
| 185 | Vreeken, Hilde L; van Rens, Ger H M B; Knol, Dirk L; van Reijen, Nadja A; Kramer, Sophia E; Festen, Joost M; van Nispen, Ruth M A | Dual sensory loss: A major age-related increase of comorbid hearing loss and hearing aid ownership in visually impaired adults. | 2014 | Geriatrics & gerontology international |
| 186 | Wahl, Hans-Werner; Heyl, Vera; Drapaniotis, Philipp M; Hormann, Karl; Jonas, Jost B; Plinkert, Peter K; Rohrschneider, Klaus | Severe vision and hearing impairment and successful aging: a multidimensional view. | 2013 | The Gerontologist |
| 187 | Williams, Nicole; Phillips, Natalie A; Wittich, Walter; Campos, Jennifer L; Mick, Paul; Orange, Joseph B; et al. | Hearing and Cognitive Impairments Increase the Risk of Long-term Care Admissions. | 2020 | Innovation in aging |
| 188 | Wittich, Walter; Watanabe, Donald H; Gagne, Jean-Pierre | Sensory and demographic characteristics of deafblindness rehabilitation clients in Montreal, Canada. | 2012 | Ophthalmic & physiological optics |
| 189 | Xie, Tian; Liu, Danxia; Guo, Jing; Zhang, Bo | The longitudinal effect of sensory loss on depression among Chinese older adults. | 2021 | Journal of affective disorders |
| 190 | Yamada, Yukari; Denkinger, Michael D; Onder, Graziano; Finne-Soveri, Harriet; van der Roest, Henriette; Vlachova, Martina; Richter, Tomas; Gindin, Jacob; Bernabei, Roberto; Topinkova, Eva | Impact of dual sensory impairment on onset of behavioral symptoms in European nursing homes: results from the Services and Health for Elderly in Long-Term Care study. | 2015 | Journal of the American Medical Directors Association |
| 191 | Yamada, Yukari; Denkinger, Michael D; Onder, Graziano; Henrard, Jean-Claude; van der Roest, Henriette G; Finne-Soveri, Harriet; Richter, Tomas; Vlachova, Martina; Bernabei, Roberto; Topinkova, Eva | Dual Sensory Impairment and Cognitive Decline: The Results From the Shelter Study. | 2016 | The journals of gerontology. Series A, Biological sciences and medical sciences |
| 192 | Yamada, Yukari; Denkinger, Michael D; Onder, Graziano; van der Roest, Henriette G; Finne-Soveri, Harriet; Bernabei, Roberto; Topinkova, Eva | Joint Associations of Dual Sensory Impairment and No-Activity Involvement With 1-Year Mortality in Nursing Homes: Results From the SHELTER Study. | 2016 | The journals of gerontology. Series A, Biological sciences and medical sciences |
| 193 | Yamada, Yukari; Vlachova, Martina; Richter, Tomas; Finne-Soveri, Harriet; Gindin, Jacob; van der Roest, Henriette; et al. | Prevalence and correlates of hearing and visual impairments in European nursing homes: results from the SHELTER study. | 2014 | Journal of the American Medical Directors Association |
| 194 | Yorgason J.B.; Tanner C.T.; Richardson S.; Hill M.M.Y.S.; Stagg B.; Wettstein M.; Ehrlich J.R. | The Longitudinal Association of Late-Life Visual and Hearing Difficulty and Cognitive Function: The Role of Social Isolation | 2022 | Journal of aging and health |
| 195 | Zhang, Y; Ge, M; Zhao, W; Liu, Y; Xia, X; Hou, L; Dong, B | Sensory Impairment and All-Cause Mortality Among the Oldest-Old: Findings from the Chinese Longitudinal Healthy Longevity Survey (CLHLS). | 2020 | The journal of nutrition, health & aging |
| 196 | Zhao, Xiaohuan; Zhou, Yifan; Wei, Kunchen; Bai, Xinyue; Zhang, Jingfa; Zhou, Minwen; Sun, Xiaodong | Associations of sensory impairment and cognitive function in middle-aged and older Chinese population: The China Health and Retirement Longitudinal Study. | 2021 | Journal of global health |
| 197 | Zhou Y.; Hu Y.; Luo J.; Li Y.; Liu H.; Sun X.; Zhou M. | Association Between Sensory Loss and Falls Among Middle-Aged and Older Chinese Population: Cross-Sectional and Longitudinal Analyses | 2022 | Frontiers in Medicine |
