# Supplementary material for: Prevalence and impact of combined vision and hearing (dual sensory) impairment: A scoping review
Source: PLOS Glob Public Health. 2023 May 16;3(5):e0001905. doi: 10.1371/journal.pgph.0001905 (PMC10187940; doi:10.1371/journal.pgph.0001905)
Supplement: S1 Text — (DOCX) [file pgph.0001905.s002.docx]

**S1 Text: Search strategy**

**Search strategy (Medline)**

1     exp hearing loss/ (68101)

2     Persons With Hearing Impairments/ (2667)

3     Hearing Disorders/ (14506)

4     (hearing adj3 (loss or impair$)).tw. (54240)

5     (deaf or deafness).tw. (29117)

6     (hard adj2 hearing).tw. (1373)

7     or/1-6 (107053)

8     exp Blindness/ (24168)

9     Vision, Low/ (3326)

10     Visually Impaired Persons/ (2391)

11     ((low$ or impair$ or partial$ or loss$) adj2 (vision or visual$ or sight$)).tw. (48082)

12     blindness.tw. (27159)

13     blind.tw. (187642)

14     (double adj1 blind$).tw. (148626)

15     double-blind$.tw. (148540)

16     (triple adj1 blind$).tw. (938)

17     triple-blind$.tw. (874)

18     or/14-17 (149490)

19     13 not 18 (49156)

20     8 or 9 or 10 or 11 or 12 or 19 (125937)

21     7 and 20 (4724)

22     (dual sensory adj2 (impair$ or loss)).tw. (159)

23     (deaf adj1 blind).tw. (263)

24     deafblind.tw. (54)

25     (deafness adj3 blindness).tw. (484)

26     (low adj2 vision adj5 (deaf$ or hearing)).tw. (21)

27     (visual adj2 impair$ adj5 (deaf$ or hearing)).tw. (450)

28     (vision adj2 loss adj5 (deaf$ or hearing)).tw. (245)

29     (sight adj2 loss adj5 (deaf$ or hearing)).tw. (16)

30     (sight adj2 impair$ adj5 (deaf$ or hearing)).tw. (13)

31     (hearing adj2 loss adj5 (blind$ or vision)).tw. (439)

32     (hearing adj2 impair$ adj5 (blind$ or vision)).tw. (366)

33     or/22-32 (2155)

34     21 or 33 (5031)

35     Prevalence/ (283471)

36     Epidemiology/ (12307)

37     Epidemiological Monitoring/ (6990)

38     prevalence.tw. (602128)

39     impact$.tw. (1048808)

40     exp Mortality/ (373797)

41     Death Certificates/ (5237)

42     Life Expectancy/ (17194)

43     years lived.tw. (905)

44     mortality.tw. (728960)

45     death$.tw. (788627)

46     survival.tw. (911110)

47     (fatality or fatalities).tw. (26740)

48     exp Activities of Daily Living/ (99758)

49     ((everyday or function$) adj2 living).tw. (2813)

50     Social Support/ (69137)

51     Social isolation/ (13010)

52     (participat$ or autonomy or independence or independent).tw. (1387187)

53     Interpersonal Relations/ (70704)

54     Communication Disorders/ (2220)

55     (social$ adj3 (factor$ or interact$ or isolat$ or network$ or support$ or participat$ or relationship$)).tw. (116790)

56     (communicat$ or relationship$).tw. (1523571)

57     Vulnerable Populations/ (10037)

58     (vulnerab$ or maltreatment or harm or abuse or protect$ or safeguard$).tw. (1044893)

59     adult protection.tw. (38)

60     at risk.tw. (168471)

61     "Quality of Life"/ (188486)

62     Value of Life/ (5685)

63     Quality Adjusted Life Year/ (11816)

64     (quality adj2 life).tw. (269976)

65     (QUALY$ or DALY$ or HRQOL).tw. (18549)

66     (sf36 or sf 36 or short form 36 or shortform 36).tw. (24982)

67     life satisfaction.tw. (7329)

68     (well adj1 being).tw. (73751)

69     (self adj2 rate$ adj2 health$).tw. (7593)

70     Accidental Falls/ (23542)

71     Risk/ (120960)

72     (fall$ or accident$).tw. (305836)

73     Cognitive Dysfunction/ (15306)

74     Dementia/ (49736)

75     dementia.tw. (103349)

76     (cognitive adj2 (function$ or impair$)).tw. (114982)

77     (cognitive adj2 (declin$ or dysfunction$)).tw. (36560)

78     Mental Health/ (36585)

79     Anxiety Disorders/ (32993)

80     (anxiety or anxious).tw. (188314)

81     Depressive Disorder/ (71978)

82     (depression or depressed).tw. (378183)

83     mental health.tw. (135687)

84     or/35-83 (7581870)

85     34 and 84 (2230)

86     exp case reports/ (2079015)

87     (case adj2 report$).tw. (482133)

88     86 or 87 (2178800)

89     85 not 88 (2024)

90     limit 89 to (address or autobiography or bibliography or biography or clinical trial, veterinary or clinical trials, veterinary as topic or comment or editorial or "expression of concern" or festschrift or interactive tutorial or interview or letter or news or observational study, veterinary or personal narrative or portrait or randomized controlled trial, veterinary or video-audio media or webcast) (29)

91     89 not 90 (1995)
