# Supplementary material for: Prevalence and impact of combined vision and hearing (dual sensory) impairment: A scoping review
Source: PLOS Glob Public Health. 2023 May 16;3(5):e0001905. doi: 10.1371/journal.pgph.0001905 (PMC10187940; doi:10.1371/journal.pgph.0001905)
Supplement: S1 Table — (DOCX) [file pgph.0001905.s003.docx]

**S1 Table:** Studies reporting prevalence of dual sensory impairment (DSI) by age group

| First author; year of publication | Study design; setting | Sample size | Definition of DSI | Estimated prevalence of DSI, % (95% CI if reported) |
| --- | --- | --- | --- | --- |
| All ages | | | | |
| **Population-based** | | | | |
| Khandekar et al. (2004); Oman | CS | 11,400 | V: <6/120 better eye H: 3FA (500,1000,2000) ≥26dB better ear | 0.25 (0.15, 0.34); Partial double disability: 0.35 (0.24, 0.47) |
| Mactaggart et al. (2017); India& | CS | 3,574 | V: <6/12 better eye H: ≥41 dB adults; ≥35 children PTA better ear | 1.91 (1.52, 2.38) |
| **Rehabilitation centre or register-based** | | | | |
| Dammeyer et al. (2010); Denmark | CS; register | 190 | V: <6/60 eye not specified H: 3FA (500,1000,2000) ≥80dB; ear not specified | 0.003  1:15 000 children, 1:34 000 adults |
| Wittich et al. (2012); Canada | Case series report; rehab centre | 564 | V: <6/18 better eye H: ≥25dB PTA better ear | 0.01 (0.01, 0.16) |
| Children | | | | |
| Linden-Bostrom et al. (2015); Sweden | CS; school | 7,793 | V/H: self-report single binary question | 0.30 (0.18, 0.45) |
| Only adults (≥18 years) | | | | |
| **Population-based** | | | | |
| Armstrong et al. (2016); USA | CS | 2006: 2,591 2010: 3,956 | V/H: self-report single binary question | 2006: 2.70 (2.11,3.40);  2010: 2.96 (3.45, 3.53) |
| Armstrong et al. (2021); USA | CS | ARIC: 936  BSLA: 420 | V/H: self-report single binary question | ARIC: 44.3 (41.2,47.5)  BSLA: 17.4 (13.90, 21.40) |
| Caban et al. (2005); USA** | CS | 195,801 | V: self-report single categorical question H: self-report single binary question | 3.3 (3.20, 3.40) |
| Lam et al. (2006); USA | CS | 116,796 | V/H: self-report single binary question | 1.25 (1.18, 1.32) |
| Loprinzi et al. (2013)^^; USA | CS | 1,445 | V: ≤6/12 either eye H: >25dB PTA better ear | 2.0 (1.34, 2.87) |
| Loprinzi et al. (2013); USA^^$ | CS | 567 | V/H: self-report single question with Likert scale | 3.0 (1.80, 4.75) |
| Swenor et al. (2013); USA | CS | NS | V: <6/12 better eye  H: >25 PTA better ear | 40-49 years: 0.1; ≥80 years: 11.3 |
| Olakunde et al. (2020); USA | CS | 468,303 | V/H: self-report, single binary question | 0.30 (0.20, 0.31) |
| Pardhan et al. (2020); Spain | CS | 23,809 | V/H: self-report, single question categorical | 3.90 (3.70, 4.20) |
| Reed et al. (2020); USA | CS/RCS; | 10,748 | V/H: self-report, single question Likert | 1.13 (0.90, 1.30) |
| Khurana et al. (2021)’ England | CS | 7,546 | V: self-report, single question with Likert scale  H: self-report, single question binary | 3.72 (3.30, 4.18) |
| Dammeyer et al. (2013); Denmark | CS | 916 | not specified | 0.02 (0.00, 0.07) |
| Jung et al. (2022); Korea$ | PCS | 771,128 | V: <6/12 better eye  H: >40dB PTA at least one ear | 1.13 (1.11, 1.15) (aged ≥30 years) |
| **Clinic- or register-based** | | | | |
| Smith et al. (2008); USA | RCS; clinic | 400 | V: <6/12 better eye H: ≥40dB PTA better ear | 5.0 (3.1, 6.9) |
| Meuwese-Jongejeugd et al. (2008); The Netherlands$ | CS; other | 1,359 | V: <6/12 better eye  H: >25dB PTA better ear | 5.00 (3.90, 6.20) |
| Older adults | | | | |
| 40+ | | | | |
| **Population-based** | |  |  |  |
| Dawes et al. (2014); United Kingdom | CS | 116,682 | V: <6/7.5 in better eye H: poor performance on Digit Triple Test | 3.10 (3.30, 3.20) |
| Fischer et al. (2009); USA | PCS | 1,854 | V: <6/12 better eye H: >25dB PTA better ear | 7.2 (6.10, 8.50) |
| Miyawaki et al. (2019); Japan | RCS | 9,522 | V/H: self-report single categorical question | 0.97 (0.77, 1.19) |
| Ma et al. (2021); China### | PCS | 13,097 | V/H: self-report single question with Likert scale | 5.50 (5.10, 5.90) |
| Liu et al. (2022); China### | PCS | 13,690 | V/H: self-report multiple questions | 10.10 (9.60, 10.60) |
| Hajek et al (2022); Germany | PCS | 5,138 | V/H: self-report single question binary | 15.9 (14.9, 16.9) |
| Zhao et al. (2021); China### | CS;pop | 13,914 | V/H self-report single question categorical | 2.10 (1.90, 2.40) |
| **Clinic- or register-based** | |  |  |  |
| Ho et al. (2021); Singapore | CS; care home | 123 | V: <6/12 better eye  H: >40dB PTA better ear | 78.90 (71.60, 86.10) |
| ≥45 years | | | | |
| Fuller et al. (2018); USA | CS | 7,210,535 | V/H: self-report single binary question | 1.62 (1.61, 1.64) |
| Klein et al. (1998); USA | PCS | 3,397 | V: poor ARMD grade (using grading system) H: >25dB in better ear | 15.10 (13.91, 16.35) |
| Lee et al. (2005); USA | PCS | 60,997 | V/H: self-report single binary question | 2.35 (2.23, 2.47)  Higher prevalence in M |
| Mick et al. (2018); Canada* | CS | 21,241 | V/H: self-report single question with Likert scale | 1.90 (1.60, 2.10) |
| Mick et al. (2021); Canada* | CS | 29,007 | V: <6/9.5 better eye H: >25dB PTA better ear | 9.56 (9.22, 9.90) |
| Sun et al. (2020); China### | PCS | 37,076 | V/H: clinical measurement, but not VA or audiometry (clinician judgment) | 20.96 (20.60, 21.40) |
| Rong et al. (2020); China### | CS | 10,575 | V: self-report multiple questions  H: self-report single question with Likert scale | 58.60 (57.90, 59.30) |
| ≥50 years | | | | |
| **Population-based** | | | | |
| Kiely et al. (2016); Australia# | PCS | 4,160 | V: <6/12 eye not specified H: >25dB PTA better ear | 8.15 (7.33, 9.02) |
| Liljas et al. (2018); England | PCS | 4,621 | V/H: self-report single question with Likert scale | 3.87 (3.33, 4.47) |
| Maharani et al. (2020); USA | CS | 19,618 | V/H: self-report single question with Likert scale | 5.02 (4.71, 5.33) |
| Mitoku et al. (2016); Japan | PCS | 1,754 | V: VA chart definition not clear H: self-report single categorical question | 18.24 (16.46, 20.13) |
| Maharani et al. (2018); Multiple countries | CS | 45,805 | V/H: self-report single question with Likert scale | England: 5.89 (5.46, 6.33)  USA: 7.10 (6.67, 7.76) |
| Viljanen et al. (2014); Multiple European countries | CS | 27,536 | V/H: self-report single categorical question | Pooled: 5.9   - Belgium: 5.40 (4.70, 6.19) - Sweden: 1.70 (1.27, 2.23) - France: 6.90 (6.04, 7.87) - Italy: 10.30 (99.13, 11.54) - Austria: 4.00 (3.16, 5.00) - Denmark: 4.80 (3.84, 5.99) - Germany: 4.80 (4.05, 5.63) - Greece: 5.20 (4.38, 6.10) - Switzerland: 1.60 (0.90, 2.56) - Spain: 9.40 (8.24, 10.64) - The Netherlands: 4.00 (3.31, 4.78) |
| Graue-Hernández et al. (2019); Mexico | CS | 1,511 | V: <6/18 better eye H: self-report multiple question | 4.37 (3.39, 5.52) |
| Beall et al. (1986); Nepal | CS | 117 | V: VA ≤6/60 better eye H: ≥60dB; frequencies/average NS; ear NS | Overall prevalence not presented; 50-60 year-old men: 2.1;  70 year-old men: 29.0 |
| Bright et al. (2020); Multiple& | CS | Cameroon: 3,567  India: 3,573 | V: <6/18 better eye H: ≥41dB PTA in better ear | India: 4.40 (3.00, 6.40)  Cameroon: 4.80 (3.00, 7.70) |
| Shakarchi et al. (2020); USA | CS;pop | 13,092 | V/H: self-report single question with Likert scale | 8.10 (7.64, 8.58) |
| Ogliari et al. (2021); Multiple | PCS | 50,986 | V/H: self-report single question with Likert scale | 7.60 (7.30, 7.80) |
| **Clinic, care home or register based** | | | | |
| Mudie et al. (2018); USA$ | CS; clinic | 220 | V: mean deviation on visual field testing worse than -5dB better eye H: >25dB better ear | 19.53 (14.46, 25.47) |
| Vreeken et al. (2014); Multiple European countries | CS; vision rehab centre | 1,396 | V/H: self-report multiple question | Pooled: 44.8 (42.2,47.5)   - Belgium: 32.71 (27.14, 38.67) - The Netherlands: 47.83 (44.82, 50.85) |
| Yamada et al. (2014); Multiple European countries | CS; care home | 4,007 | V/H: self-report single categorical question | Pooled: 31.8   - Czech Republic: 39.76 (35.41, 44.23) - England: 14.20 (11.30, 17.57) - Finland: 21.27 (17.54, 25.38) - France: 34.92 (30.67, 39.35) - Germany:33.20 (29.06, 37.54) - Israel: 32.83 (28.83, 37.02) - Italy: 49.90 (45.52, 54.29) - The Netherlands: 27.19 (23.50, 31.12) |
| Lach et al. (2019); NS | CS; care home | 225 | V: <6/15; eye not specified H: ≥40dB PTA better ear | 29.78 (23.88, 36.21) |
| ≥55 years | | | | |
| **Population-based** | | | | |
| Chia et al. (2006); Australia#  Schneider et al.# (2012); Australia | PCS | 2,015 | V: <6/12 better eye H: >25dB PTA better ear | Presenting VA: 6.04 (5.04, 7.23)  Corrected VA; 2.1 corrected VA  Prevalence higher in women |
| Gopinath et al. (2014); Australia# | PCS | 2,443 | V: <6/12 better eye H: >25dB PTA better ear | 3.89 (3.16, 4.73) |
| Gopinath et al. (2021); Australia# | PCS | 1,085 | V: <6/12 better eye H: >25dB PTA better ear | 13.50 (11.57, 15.73) |
| Capella-McDonnall et al. (2005); USA** | CS | 9,832 | V: self-report single binary question H: self-report single categorical question | 7.34 (6.69, 8.02) |
| Reuben et al. (1999); USA | PCS | 5,677 | V: <6/12 better eye H: >40dB at 1 or/and 2kHz in better ear  (also measured V/H: self-report single question binary) | Clinical measurement: 4.60 (3.24, 6.31) Self-report: 4.09 (3.17, 5.20) |
| Raina et al. (2004); Canada | CS | 16,613 | V/H: self-report single binary question | 3.00 (2.74, 3.27) |
| **Clinic-based** | | | | |
| Keller et al. (1999); USA | CS; clinic | 576 | Vision: ≤20/70 in better eye (near vision) Hearing: fail whisper voice test | 13.02 (10.38, 16.05) |
| ≥60 years | | | | |
| **Population-based** | | | | |
| Kulmala et al. (2009); Finland$ | PCS | 428 | V: <6/60 eye not specified H: ≥21 dB PTA better ear | 17.29 (13.83, 21.21) |
| Mueller-Schotte et al. (2018); Netherlands | PCS | 9,319 | V/H: self-report single binary question | 18.17 (17.39, 18.97) |
| Chou et al. (2004); Hong Kong | CS | 2,003 | V/H: self-report single categorical question | 6.50 (5.45, 7.66) |
| Kwon et al. (2015); South Korea^^^ | CS | 5,260 | V: <6/18 better eye H: >40dB PTA better ear | 5.10 (4.52, 5.72) |
| Tareque et al. (2019); Singapore | PCS | 3,452 | V/H: self-report single question with Likert scale | 11.62 (10.57, 12.73) |
| Heine et al. (2019); China### | CS | 8,268 | V/H: self-report single question with Likert scale | 57.20 (56.12, 58.27) |
| Deepthi et al. (2012); India | CS | 257 | V: <6/18 better eye H: >25dB PTA better ear | 37.14 (29.97, 44.76) |
| Harithasan et al. (2020); Malaysia | CS | 229 | V: <6/12 better eye H: ≥26 PTA better ear | 8.30 (5.07, 12.65) |
| Byeon et al. (2021); Korea | PCS | 6,520 | V/H: self-report single question with Likert scale | 40.40 (39.20, 41.60) |
| Gadkaree et al. (2016); USA | CS | 276,233 | V/H self-report single question with Likert scale | 9.87 (6.36, 14.44) |
| Ramamurthy et al. (2014); India | CS | 175 | V: <6/18 better eye  H: 3FA >40dB better ear | 17.71 (12.80, 24.00) |
| Phua et al. (2022); Singapore | CS | 4,077 | V/H: self-report single question with Likert scale | 12.80 (11.80, 13.90) |
| **Clinic or other based** | | | | |
| Hickson et al. (1999); Australia | PCS; community centres, senior citizens organisations, local newspapers, TV | 240 | V: <6/9 eye not specified H: >25dB PTA better ear | 18.75 (14.02, 24.27) |
| Cosh et al. (2018); Norway | PCS; clinic | 2,890 | V: <6/9 eye not specified H: self-report single question binary | 6.77 (5.57, 7.92) |
| Marmamula et al. (2021); India | CS; CH | 867 | V: <6/18 better eye  H: self-report multiple questions | 5.80 (4.30, 7.50) |
| Kwan et al. (2022); China | PCS; CH | 2,233 | V/H: not specified/unclear | 12.20 (10.90, 13.70) |
| ≥65 years | | | | |
| **Population-based** | | | | |
| Heine et al. (2019); Australia | RCS | 1,000 | V/H: self-report single categorical question | 11.00 (9.13, 13.11) |
| Kiely et al. (2013); Australia*** | PCS | 1,611 | V: <6/12 better eye H: >25dB PTA better ear | 21.11 (18.87, 23.48) |
| Liu et al. (2015); USA | PCS | 3,871 | V: self-report single binary question H: self-report single categorical question | 4.73 (4.08, 5.44) |
| Simning et al. (2018); USA | PCS | 7,507 | V/H: self-report single binary question | 1.30 (1.04, 1.56) |
| Fisher et al. (2014); Canada | PCS | 4,926 | V: <6/15 better eye H: ≥35dB PTA better ear | 6.96 (6.27, 7.71) |
| Schneck et al. (2012); USA | CS | 446 | V: <6/18 either eye H: >40dB PTA better ear | 3.10 (1.73, 5.21) |
| Campbell et al. (1999); USA** | CS | 8,767 | V/H: self-report multiple question | 8.6 (7.9, 9.3) |
| Deardorff et al. (2019); USA | PCS | 24,009 | V/H: self-report single categorical question | 18.22 (17.74, 18.72) |
| Forbes et al. (1991); Canada | CS | 132,337 | V/H: self-report question type not clear | 4.80 (4.77, 4.83) |
| Grue et al. (2008); Norway | CS | 332 | V: <6/7.5 better eye H: ≥30dB PTA better ear | 30.1 |
| Kim et al. (2015); South Korea^^^ | CS | 3,636 | V: <6/18 either eye H: >40dB PTA better ear | 6.16 (5.40, 6.99) |
| Chou et al. (2008); England** | PCS | 3,782 | V/H: self-report single question with Likert scale | 7.10 (6.31, 7.96) |
| Lyu et al. (2018); South Korea | CS | 3,831 | V/H: self-report single question with Likert scale | 5.85 (5.12, 6.63) |
| Kuo et al (2021); USA | PCS | 7,562 | V/H: self-report multiple questions | 3.10 (2.70, 3.50) |
| Assi et al. (2021); USA | CS | 7,124 | V/H: self-report single question binary | 4.10 (3.70, 4.60) |
| Xie et al. (2021); China### | PCS | 6,563 | V/H: self-report single question with Likert scale | 55.67 (54.40, 56.90) |
| Harada et al. (2008); Japan | CS | 843 | V:<6/12 better eye  H: >30dB 1kHz (ear not specified) | 9.73 (7.81, 11.93) |
| Yorgasson et al. (2022); USA | PCS | 6,338 | V/H: self-report multiple questions | 2.20 (1.90, 2.60) |
| Assi et al. (2020); USA | CS | 10,783 | V/H: self-report single question with Likert scale | 19.98 (19.20, 20.70) |
| Kileen et al. (2022)##; USA | PCS | 7,593 | V/H: self-report multiple questions | 3.30 (2.90, 3.80) |
| Mah et al. (2020); Malaysia | CS | 210 | V: <6/12 better eye  H: >25dB PTA better ear | 10.50 (6.70, 15.40) |
| Steinman et al. (2021)##; USA | PCS | 4,636 | V/H: self-report multiple questions | 2.40 (2.00, 2.90) |
| Maruta et al. (2020); Japan | RCS | 2,190 | V: visual acuity chart (not clear type)  H: self-report single question categorical | 13.50 (12.10, 15.00) |
| Haanes et al. (2021); Denmark | CS | 74 | V: <6/12 better eye, and self report  H: >25dB better ear, and self-report | 22.00 (12.90, 32.70) |
| **Clinic, care home or other based** | | | | |
| Davidson et al. (2019); Canada^ | CS; care home | 352,656 | V/H: self-report single categorical question | 20.47 (20.34, 20.60) |
| Guthrie et al. (2016); Canada^ | CS; home care | 218,850 | V/H: self-report single categorical question | 16.71 (16.56, 16.87) |
| Soto-Perez-de-Celis et al. (2018); USA | CS; clinic | 750 | V/H: self-report single categorical question | 7.33 (5.57, 9.43) |
| Cacchione et al. (2003); USA | CS; clinic | 114 | V: ≤20/70 in better eye (near vision) H: fail whisper voice test | 24.56 (16.98, 33.51) |
| Guthrie et al. (2018); Canada | CS; care home; long term care | 402,402 | V/H: self-report single categorical question | Home care clients: 3.65 (3.58, 3.72)  Long-term care residents: 0.97 (0.91, 1.03) |
| Guthrie et al. (2016); Multiple | CS; home care; long term care | Home care clients: 550,360 Long-term Care residents: 261,296 | V: self-report single binary question H: self-report single categorical question | Home care clients:   - Canada = 14.48 (14.39, 14.58), - US = 15.48 (17.7, 16.26), - Belgium = 8.81 (8.36, 9.28) - Finland 13.39 (12.86, 13.93)   Long term care residents:   - Canada = 25.80 (25.60, 26.00), - US=9.73 (9.51, 9.96), - Belgium = 33.86 (30.49, 37.36) - Finland = 22.17 (24.14, 23.24) |
| Lyu et al. (2018); South Korea | CS | 3,381 | V/H: self-report single question with Likert scale | 5.85 (5.12, 6.63) |
| Shakarchi et al. (2021), USA | PCS | 7,648 | V/H: self-report single question with Likert scale | 7.33 (5.57, 9.43) |
| Morandi et al. (2021); Italy$ | CS; O | 3,038 | V/H: clinician judgement | 9.74 (8.71, 10.85) |
| ≥70 years | | | | |
| **Population-based** | |  |  |  |
| Clark et al. (1999); Australia*** | CS | 1,052 | V: <6/12 eye not specified H: >40dB PTA better ear | 9.70 (7.97, 11.65) |
| Kiely et al. (2018); Australia | CS | 1,393 | V: <6/12 eye not specified H: self-report single question with Likert scale | 4.88 (3.81, 6.15) |
| Bergman et al. (2001); Sweden | PCS | 954 | V: <6/7.5 in better eye H: >=40dB PTA in better ear; | 2.29 (1.40, 3.40) |
| Brennan et al. (2005); USA**  Brennan et al. (2006); USA** | PCS | 5,151 | V/H: self-report single categorical question | 21.00 (19.90, 22.14) |
| Crews et al. (2004); USA** | PCS | 9,447 | V/H: self-report single binary question | 8.20 (7.66, 8.78) |
| Green et al. (2013); USA | RCS | 2,000 | V: <6/12 eye not specified H: self-report single question binary | 3.05 (2.34, 3.90) |
| Tinetti et al. (1995); USA | PCS | 1,103 | V: >50% impaired on chart (unclear) H: fail whisper voice test (> 5 words missed) | 11.00 (9.06, 13.20) |
| ≥75 years | | | | |
| **Population-based** | | | | |
| Lupsakko et al. (2002); Finland | CS | 470 | V: <6/15 eye not specified H: self-report single question binary | 7.02 (4.88, 9.72) |
| Bouscaren et al. (2019); France | PCS | 4,010 | V/H: self-report single binary question | 1.7 |
| Pabst et al. (2021); Germany | PCS | 2,051 | V/H: self-report multiple questions | 6.50 (5.80, 7.40) |
| Hwang et al. (2020); USA | PCS | 3,497 | V/H: self-report single question with Likert scale | 5.10 (4.20, 6.10) |
| **Clinic-based** | | | | |
| Grue et al. (2009); Multiple | CS; clinic | 770 | V/H: self-report single categorical question | Pooled: 20.1 (pooled)   - Denmark: 45.00 (37.14, 53.05) - Finland: 9.80 (5.31, 16.13) - Iceland: 17.80 (12.19, 24.73) - Norway: 10.60 (6.31, 16.47) - Sweden: 15.60 (10.37, 22.20) |
| ≥80 years | | | | |
| **Population-based** | |  |  |  |
| Zhang et al. (2020); China | PCS; clinic | 8,788 | V/H: self-report single categorical question | 9.79 (9.17, 10.43) |
| **Non population-based** | |  |  |  |
| Haanes et al. (2014); Norway | CS; care home | 100 | V: <6/15 better eye H: >40dB PTA better ear | 28.00 (19.14, 38.22) |
| ≥95 years | | | | |
| Cimarolli et al. (2014); USA | CS; clinic | 119 | V/H: self-report single question with Likert scale | 37.61 (28.82, 47.03) |
| Other | | | | |
| Dupuis et al. (2014); Canada | CS | 301 | V: <6/12 better eye H: ≥26 PTA better ear | Distance vision only: 7  Distance and near vision: 7.5 |
| Parada et al. (2021) | PCS | 1,383 | V: <6/12 better eye H: > 25dB PTA better ear | 18.1 (16.2, 20.3) |

*CS=cross sectional; PCS=prospective cohort study; RCS=retrospective cohort study; pop=population-based; V=vision; H=hearing; PTA=pure tone audiometry ARMD=age related macular degeneration;*

^Residential Assessment for Home Care (RAI-HC) study - different years (reported separately)

^^National Health And Nutrition Examination Survey (NHANES) – different sample size, different definition (reported separately)

^^^Korean Health And Nutrition Examination Survey (KHANES) – different age groups (reported separately)

* Canadian Longitudinal Study on Ageing (CLSA) – same wave, different measurement of DSI (reported separately)

** National Health Interview Survey; Longitudinal Supplement on Ageing (NHIS/LSOA) – different years, or different age groups (reported separately)

***Australian Longitudinal Study of Ageing (ALSA) – different age groups (reported separately)

# Blue Mountains Eye Study (BMES) – different age groups and samples (reported separately)

## National Health and Ageing Trends (NHATS) – different waves (reported separately)

### China Health and Retirement Longitudinal Study (CHARLS) – different waves, and age groups (reported separately)

& Same population-based survey (India) – different age groups (reported separately)

$ prevalence reported in a subpopulation
