# Supplementary material for: Prevalence and impact of combined vision and hearing (dual sensory) impairment: A scoping review
Source: PLOS Glob Public Health. 2023 May 16;3(5):e0001905. doi: 10.1371/journal.pgph.0001905 (PMC10187940; doi:10.1371/journal.pgph.0001905)
Supplement: S2 Table — (DOCX) [file pgph.0001905.s004.docx]

**S2 Table:** Reports Measuring Psychosocial Health Outcomes for people with dual sensory impairment (DSI)

| Study author, year | Country, Region | Country income group (at time of publication) | Study design | Study setting | Age group (years) | Sample size total (n DSI) | Comparator group  **No DSI:** people with HI only, VI only, or neither HI nor VI  **Single impairment:** people with HI only or VI only  **HI only:** people with HI only  **VI only:** people with VI only  **No SI**: people with no HI or VI | Definition of DSI | Outcome category | Result summary (outcome in DSI group relative to other group) |
| --- | --- | --- | --- | --- | --- | --- | --- | --- | --- | --- |
| Amini, 2010 | Iran, North Africa and Middle East | Upper middle | Cross sectional | Other | Adults (18+) | 248 (not stated) | No comparator group | V/H: Not specified | Quality of life | No comparator group |
| Armstrong, 2016 | USA, North America | High | Cross sectional | Population | Adults (18+) | 2006: 2,481 (70);  2010: 3,853 (117) | No SI; HI only; VI only | V/H: self-report single binary question | Depression; Quality of life | Varied |
| Assi, 2021 | USA, North America | High | Cross sectional | Population | Older adults (65+) | 7,124 | No SI; Single impairment | V/H: self-reported single question binary | Depression; Cognitive disorders | Worse |
| Bodsworth, 2011 | UK, Western Europe | High | Cross sectional | Register | Adults (18+)) | 539 (539) | Compared with data from other studies on people without VI/HI, and people with HI or people with VI | V/H: Not specified | Psychological wellbeing | Worse |
| Byeon, 2021 | South Korea | High | Prospective cohort | Population | Older adults (60+) | 6,520 (2631) | No SI; single impairment | V/H: self-reported single question Likert | Cognitive disorders | Worse |
| Cacchione, 2003 | USA, North America | High | Cross sectional | Clinic | Older adults (65+) | 114 (28) | No DSI | V: <=20/70 better eye (near vision) H: Fail whisper voice test | Acute confusion | Worse |
| Capella-McDonnall, 2005 | USA, North America | High | Cross sectional | Population | Older adults (55+) | 9,832 (447) | No SI; HI only; VI only | V: self-report single binary question H: self-report single categorical question | Depression | Worse |
| Chia, 2006 | Australia, Australasia | High | Prospective cohort | Population | Older adults (55+) | 2,015 (116) | No SI; single impairment | V: <6/12 better eye H: >25dB PTA better ear | Quality of life | Worse |
| Chou, 2004 | Hong Kong, Southeast Asia, East Asia, and Oceania | High | Cross sectional | Population | Older adults (60+) | 2,003 (131) | No DSI | V/H: self-report single categorical question | Depression | Worse |
| Chou, 2008 | England, UK, Western Europe | High | Prospective cohort | Population | Older adults (65+) | 3,782 (not stated) | No DSI | V/H: self-report single question with Likert scale | Depression | No difference |
| Cimarolli, 2018 | USA, North America | High | Cross sectional | Population | Older adults (95+) | 119 (not stated) | No DSI | V/H: self-report single question with Likert scale | Depression | Worse |
| Cosh, 2018 | Norway, Western Europe | High | Prospective cohort | Clinic | Older adults (60+) | 2,890 (146) | No DSI | V: <6/9 eye not specified H: self-report single question binary | Depression Neurotic and stress related disorders | Varied |
| Dalby, 2009 | Canada, North America | High | Cross sectional | Register | All ages | 182 (182) | No comparator group | V/H: self-report multiple question | Cognitive disorders;  Psychological wellbeing | No comparator group |
| Dammeyer , 2010 | Denmark, Western Europe | High | Cross sectional | Other | Adults (18+)) | 95 (95) | No comparator group | V/H: Not specified | Depression; Neurotic and stress related disorders; Schizophrenia, schizotypical and delusional;  Developmental disorder;  Behavioural disorder/symptoms | No comparator group |
| Dammeyer, 2010 | Denmark, Western Europe | High | Cross sectional | Population | Adults (18+)) | 117 (117) | No comparator group | V: <=6/60 in better eye H: 3FA (500,1000,2000) >=80dB; ear not specified | Self-evaluation of life/satisfaction with life | No comparator group |
| Dammeyer, 2014 | Denmark, Western Europe | High | Cross sectional | Deafblindness centre | Children (<18 years) | 71 (71) | Children with another developmental disorder (from other studies) | V: <6/60 eye not specified H: Deaf >80dB*; Residual hearing <=80dB; ear not specified; frequencies/average not specified | Developmental disorder | No difference |
| Davidson, 2019 | Canada, North America | High | Cross sectional | Care home | Older adults (65+) | 352,656 (72,188) | No DSI | V/H: self-report single categorical question | Cognitive disorders;  Depression; Loneliness | Worse |
| de la Fuente, 2019 | England, UK, Western Europe | High | Prospective cohort | Population | Older adults (60+) | 3,508 (239) | No SI; | V/H: self-report single question with Likert scale | Cognitive disorders | Worse |
| Deepthi, 2012 | India, South Asia | Low to middle | Cross sectional | Population | Older adults (60+) | 257 (257) | No comparator group | V: <6/18 better eye H: >25dB PTA better ear | Cognitive disorders | Study too small to judge |
| Ehn, 2018 | Sweden, Western Europe | High | Case control study | Register | Adults (18+)) | 47 (47) | No SI (reference group from the population) | V: VA chart used but definition not clear H: Based on PTA but definition not clear | Psychological wellbeing | Worse |
| Fuller, 2018 | USA, North America | High | Cross sectional | Population | Older adults (45+) | 7,210,535 (not stated) | No SI; HI only; VI only | V/H: self-report single binary question | Cognitive disorders | Worse |
| Ge, 2021 | USA, North America | High | Prospective cohort | Population | Older adults (65+) | 295 (52) | No DSI | V: <6/12 better eye  H: >25dB better ear, any frequency | Cognitive disorders | Worse |
| Gopinath, 2013 | Australia, Australasia | High | Prospective cohort | Population | Older adults (55+) | 2,812 (947) | No DSI | V: <6/12 better eye H: >25dB PTA better ear | Cognitive disorders | Worse |
| Guthrie, 2016 | Multiple, Multiple | High | Cross sectional | Other | Older adults (65+) | Home care clients: 550,360 (80,634) Long-term care residents: 261,296 (56,401) | No DSI | V: self-report single binary question H: self-report single categorical question | Depression | Varied |
| Guthrie, 2016 | Canada, North America | High | Cross sectional | Care home | Older adults (65+) | 218,850 (37,047) | No DSI | V/H: self-report multiple question | Depression | Worse |
| Hajek, 2020 | Germany, Western Europe | High | Prospective cohort | Population | Older adults (40+) | 5,138 (815) | No DSI | V/H: self-reported single question binary | Depression; Self-evaluation of life/satisfaction with life; Loneliness; Other: social isolation, self-esteem and autonomy | Worse |
| Han, 2019 | South Korea, Asia Pacific | High | Cross sectional | Population | Older adults (45+) | 5,832 (not stated) | No SI | V/H: self-report single question with Likert scale | Depression | Worse |
| Harada, 2008 | Japan, Asia Pacific | High | Cross sectional | Population | Older adults (65+) | 843 (82) | No SI | V: <6/12 better eye H: >30dB at 1k better ear | Depression | Worse |
| Harithasan, 2020 | Malaysia, Southeast Asia, East Asia, and Oceania | Upper middle | Cross sectional | Population | Older adults (60+) | 229 (19) | No DSI | V: <6/12 better eye H: >=26 PTA better ear | Depression;  Cognitive disorders;  Loneliness;  Quality of life | Varied |
| Harsthorne, 2007 | USA, North America | High | Cross sectional | Register | Children (<18 years) | 98 (55) | Children with CHARGE who were not deafblind | V/H: Parent-report that child was deaf and blind | Cognitive disorders | Varied |
| Hartshorne, 2009 | England, UK, Western Europe | High | Cross sectional | CHARGE syndrome foundation mailing list | Children (<18 years) | 87 (18) | Children with CHARGE who were not deafblind | V/H: Self-reported ""no better than both moderate hearing impairment in the best ear and moderate vision impairment in the best eye" | Sleep disturbance | Worse |
| Heine, 2019 | China, Southeast Asia, East Asia, and Oceania | Upper middle | Cross sectional | Population | Older adults (60+) | 8,268 (not stated) | No DSI | V/H: self-report single question with Likert scale | Depression;  Self-evaluation of life/satisfaction with life | Worse |
| Heine, 2019 | Australia, Australasia | High | Retrospective cohort | Population | Older adults (65+) | 1,000 (110 in 1994; 50 in 2004) | No SI | V/H: self-report single question categorical | Depression | Worse |
| Hersh, 2013 | Multiple, Western Europe | High | Qualitative | Register | Unknown | 27 (27) | No comparator group | V/H: Not specified | Quality of life | No comparator group |
| Ho, 2021 | Singapore | High | Cross sectional | Care home | Older adults (40+) | 123 (97) | No DSI | V: <6/12 better eye  H: PTA >40dB better ear | Quality of life | Worse |
| Hong, 2016 | Australia, Australasia | High | Prospective cohort | Population | Older adults (49+) | 3,654 (93) | No SI | V: <6/12 worse eye H: >40dB in better ear | Cognitive disorders | No difference |
| Hovaldt, 2022 | Denmark, Western Europe | High | Cross sectional | Register | Older adults (50+0 | 290 (290) | No comparator group | V/H: not specified (register) | Thoughts of self-harm or suicide | No comparator group |
| Huddle, 2016 | USA, North America | High | Cross sectional | Population | Older adults (70+) | 1,669 (291) | No SI | V: self-report single question binary H: >=25dB PTA in better ear | Psychological wellbeing | Worse |
| Hwang, 2020 | USA, North America | High | Prospective cohort | Population | Older adults (75+) | 2,051 (104) | No DSI | V/H: self-reported multiple questions | Cognitive disorders | Varied |
| Khil, 2015 | Germany, Western Europe | High | Cross sectional | Population | 25-74 | 1,102 (187) | No SI (no visual/auditory/olfactory/gustatory impairment) | V: <6/12 worse eye H: >30dB 3FA in worse ear | Quality of life | Varied |
| Khurana, 2021 | England, Western Europe | High | Cross sectional | Population | 16+ | 7,546 (281) | No DSI | V: self-report single question Likert  H: self-report single question binary | Suicide ideation and suicide attempt | Worse |
| Kiely, 2013 | Australia, Australasia | High | Prospective cohort | Population | Older adults (65+) | 1,611 (not stated) | No SI | V: <6/12 better eye H: >25dB PTA better ear | Depression | Worse |
| Kiely, 2018 | Australia, Australasia | High | Cross sectional | Population | 72-79 | 1,393 (68) | No SI | V: <6/12 eye not specified H: >25dB PTA better ear | Psychological wellbeing | Varied |
| Killeen, 2022 | USA, North America | High | Prospective cohort | Population | Older adults (65+) | 7,593 (331) | No DSI | V/H: self-report multiple questions | Depression | Worse |
| Kim, 2015 | South Korea, Asia Pacific | High | Cross sectional | Population | Older adults (65+) | 3,636 (224) | No SI | V: <6/18 worse eye H: >40dB in better ear | Psychological wellbeing | Worse |
| Kuo, 2021 | USA, North America | High | Cross sectional | Population | Older adults (65+) | 7,562 (303) | No DSI | V/H: self-report multiple questions | Cognitive disorders | Worse |
| Kwan, 2022 | Hong Kong, Asia Pacific | High | Prospective cohort | Care home | Older adults (60+) | 2,233 (273) | No DSI | V/H: self-report single question | Cognitive disorders | Worse |
| Kwon, 2015 | South Korea, Asia Pacific | High | Cross sectional | Population | Older adults (60+) | 5,260 (268) | No SI | V: <6/18 better eye H: >40dB PTA better ear | Depression;  Neurotic and stress related disorders | The same |
| Lach, 2019 | Not specified, Not specified | Unknown | Cross sectional | Care home | Older adults (50+) | 225 (67) | No DSI | V: <6/15; eye not specified H: >=40dB PTA better ear | Depression; Neurotic and stress related disorders; Cognitive disorders | Varied |
| Lehane, 2018 | Denmark, Western Europe | High | Cross sectional | Population | Adults (18+) | 316 (183) | HI only; VI only | V: <6/9 eye not specified H: >=26 db PTA in better ear | Psychological wellbeing | No difference |
| Liljas, 2018 | England, UK, Western Europe | High | Prospective cohort | Population | Older adults (50+) | 4,621 (179) | No SI | V/H: self-report single question with Likert scale | Depression | Worse |
| Lin, 2004 | USA, North America | High | Cross sectional | Population | Older adults (65+) | 6,112 (not stated) | No DSI | V: <6/12 in better eye H: >=40dB at 2kHz in better ear | Cognitive disorders | Worse |
| Liu, 2022 | China, Asia Pacific | Upper middle | Prospective cohort | Population | Older adults (40+) | 13,690 (1,376) | No DSI | V/H: self-report multiple questions | Depression | Worse |
| Loprinzi, 2013 | USA, North America | High | Cross sectional | Population | Adults (18+)) | 567 (17) | No SI | V/H: self-report single question with Likert scale | Depression | Worse |
| Luo, 2018 | China, Southeast Asia, East Asia, and Oceania | Upper middle | Cross sectional | Population | Older adults (50+) | 250,752 (5,277) | No DSI | V: <6/120 better eye H: >40dB in better ear | Cognitive disorders | Worse |
| Lupsakko, 2002 | Finland, Western Europe | High | Cross sectional | Population | Older adults (75+) | 470 (33) | No SI | V: <6/15 better eye H: self-report single question binary | Depression | Varied |
| Lyu, 2018 | South Korea, Asia Pacific | High | Cross sectional | Population | Older adults (50+) | 3,831 (not stated) | No SI | V/H: self-report single question with Likert scale | Depression;  Cognitive disorders | Varied |
| Ma, 2021 | China, Asia Pacific | Upper middle | Prospective cohort | Population | Older adults (45+) | 13,097 (723) | No DSI | V/H: self-report, single question Likert | Episodic memory | Worse |
| Mah, 2020 | Malaysia | Upper middle | Cross sectional | Population | Older adults (65+) | 210 (22) | No DSI | V: <6/12 better eye  H: >25dB PTA better ear | Cognitive disorders | Worse |
| Maharani, 2018 | Multiple, Multiple | High | Cross sectional | Population | Older adults (50+) | 45,805 (not stated) | No SI | V/H: self-report single question with Likert scale | Cognitive disorders | Worse |
| Maharani, 2020 | USA, North America | High | Cross sectional | Population | Older adults (50+) | 19,618 (984) | No SI | V/H: self-report single question with Likert scale | Cognitive disorders | Worse |
| Marmamula, 2021 | India, South Asia | Lower middle income | Cross sectional | Care home | Older adults (60+) | 897 (50) | No DSI | V:<6/18 better eye  H: self-report multiple questions | Depression | Worse |
| Maruta, 2019 | Japan, Asia Pacific | High | Retrospective cohort | Register | Older adults (50+) | 2,190 (295) | No SI | V: VA chart definition not clear H: self-report single categorical question | Cognitive disorders | Worse |
| Maruta, 2020 | Japan, Asia Pacific | High | Retrospective cohort | Population | Older adults (65+) | 2,190 | No DSI | V: VA chart definition not clear H: self-report single categorical question | Cognitive disorders | Worse |
| McDonnall, 2009 | USA, North America | High | Retrospective cohort | Population | Older adults (50+) | 2,689 (not stated) | No SI | V/H: self-report single question with Likert scale | Depression | Worse |
| McDonnall, 2011 | USA, North America | High | Prospective cohort | Population | Older adults (50+) | 2,688 (not stated) | No SI | V/H: self-report single question with Likert scale | Depression | Worse |
| McDonnall, 2011 | USA, North America | High | Prospective cohort | Population | Older adults (50+) | 2,688 (not stated) | No SI | V/H: self-report single question with Likert scale | Depression | Varied |
| Meuwese- Jongejeugd, 2008 | The Netherlands, Western Europe | High | Cross sectional | Clinic | Adults (18+) | 1,359 (77) | Compared severity of ID, and whether or not had Down's Syndrome | V: <6/12 better eye  H: >25dB PTA better ear | Intellectual disability | Worse |
| Michalowsky, 2019 | Germany, Western Europe | High | Case control | Clinic | Older adults (65+) | 122,708 (not stated) | People without dementia | V/H: Not specified | Cognitive disorders | No difference |
| Mick, 2018 | Canada, North America | High | Cross sectional | Population | 45-85 | 21,241 (not stated) | No DSI | V/H: self-report single question with Likert scale | Loneliness | Worse |
| Mitoku, 2016 | Japan, Asia Pacific | High | Prospective cohort | Population | Older adults (50+) | 1,754 (320) | No SI | V: VA chart definition not clear H: self-report single categorical question | Cognitive disorders | Worse |
| Morandi, 2021 | Italy, Western Europe | High | Cross sectional | Hospital | Older adults (65+) | 3,038 (296) | No DSI | V/H: clinician judgement | Delirium | Worse |
| Mudie, 2018 | USA, North America | High | Cross sectional | Clinic | Older adults (50+) | 220 (42) | No SI; HI only; VI only | V: Mean deviation on visual field testing worse than -5dB better eye H: >25dB better ear | Cognitive disorders | No difference |
| Pabst, 2021 | Germany, Western Europe | High | Prospective cohort | Population | Older adults (75+) | 3,497 (229) | No DSI | V/H: self-report single question Likert | Cognitive disorders | No difference |
| Parada, 2021 | USA, North America | High | Prospective cohort | Population | Adults (not specified) | 1,383 (251) | No DSI | V: <6/12 better eye  H: >25dB PTA better ear | Cognitive disorders | Worse |
| Pardhan, 2021 | Spain, Western Europe | High | Prospective cohort | Population | 15+ | 23,089 (908) | No DSI | V/H: self-report single question categorical | Depression; anxiety | Worse |
| Pardhan, 2020 | Spain, Western Europe | High | Prospective cohort | Population | 15+ | 23,089 (908) | No DSI | V/H: self-report single question categorical | Depression; anxiety | Worse |
| Phua, 2022 | Singapore | High | Cross sectional | Population | Older adults (60+) | 4,077 (523) | No DSI | V/H: self-report single question Likert | Depression; Loneliness; Psychological wellbeing; Quality of life | Worse |
| Rong, 2020 | China, Asia Pacific | Upper middle | Cross sectional | Population | Older adults (45+) | 18,038 (10,575) | No DSI | V: self-report multiple questions  H: self-report single question Likert | Depression; Cognitive disorders | Worse |
| Simning, 2018 | USA, North America | High | Prospective cohort | Population | Older adults (65+) | 7,507 (125) | No S | V/H: self-report single binary question | Depression;  Neurotic and stress related disorders | Worse |
| Soto-Perez-de-Celis, 2018 | USA, North America | High | Cross sectional | Clinic | Older adults (65+) | 750 (55) | No SI | V/H: self-report single categorical question | Depression;  Neurotic and stress related disorders | Worse |
| Teh, 2006 | Singapore, Asia Pacific | High | Retrospective cohort | Clinic | Older adults (50+) | 112 (36) | No DSI | V: self-report single question binary H: Fail whisper voice test | Depression;  Cognitive disorders | Varied |
| Turunen-Tahari, 2017 | Sweden, Western Europe | High | Retrospective cohort | Register | Adults (18+)) | 2,319 (543) | HI only | V: self-report single question binary H: >70dB PTA in better ear | Depression;  Quality of life | Worse |
| UrquetaAlfaro, 2020 | Canada, North America | High | Cross sectional | Clinic | Older adults (65+) | 200 (73) | HI only; VI only | V/H: self-report single question categorical | Depression; Loneliness; Psychological wellbeing | No difference |
| Viljanen, 2013 | Finland, Western Europe | High | Prospective cohort | Finnish Twin Cohort (same-sex twins) | 63 to 76-year-old women twins | 434 (not stated) | No SI (no vision/hearing/balance difficulties) | V/H: self-report single question categorical | Neurotic and stress related disorders;  Self-evaluation of life/satisfaction with life | Worse |
| Wahl, 2013 | Germany, Western Europe | High | Cross sectional | Clinic | 75-94 | 430 (43) | No SI | V: <6/18 worse eye H: >=35dB PTA in better ear | Cognitive disorders;  Loneliness;  Psychological wellbeing | Varied |
| Xie, 2021 | China, Asia Pacific | Upper middle | Prospective cohort | Population | Older adults (65+) | 6,353 (3537) (baseline) 2,703 (followup) | No DSI | V/H: self-report single question Likert | Depression | Worse |
| Yamada, 2014 | Multiple, Western Europe | High | Cross sectional | Care home | Older adults (50+) | 4,007 (1,275) | No SI | V/H: self-report single categorical question | Depression;  Cognitive disorders | Worse |
| Yamada, 2015 | Multiple, Western Europe | High | Prospective cohort | Care home | Older adults (50+) | 4,156 (400) | No SI | V/H: self-report single categorical question | Behavioural disorder/symptoms | Worse |
| Yamada, 2016 | Multiple, Western Europe | High | Prospective cohort | Care home | Older adults (50+) | 1,989 (122) | No SI; Single impairment | V/H: self-report single categorical question | Cognitive disorders | Worse |
| Yorgason, 2022 | USA, North America | High | Cross sectional | Population | Older adults (65+) | 3,338 (140) | No DSI | V/H: self-report multiple questions | Cognitive disorders | Worse |
| Zhao, 2021 | China, Asia Pacific | High | Cross sectional | Population | Older adults (40+) | 13,914 (294) | No DSI | V/H: self-report single question categorical | Cognitive disorders | Worse |
