# Supplementary material for: Prevalence and impact of combined vision and hearing (dual sensory) impairment: A scoping review
Source: PLOS Glob Public Health. 2023 May 16;3(5):e0001905. doi: 10.1371/journal.pgph.0001905 (PMC10187940; doi:10.1371/journal.pgph.0001905)
Supplement: S3 Table — (DOCX) [file pgph.0001905.s005.docx]

**S3 Table**: Reports measuring participation outcomes for people with dual sensory impairment (DSI)

| Study author, year | Country, Region | Country income group (at time of publication) | Study design | Study setting/recruitment | Age group (years) | Sample size (n DSI) | Comparator group  **No DSI:** people with HI only, VI only, or neither HI nor VI  **Single impairment:** people with HI only or VI only  **HI only:** people with HI only  **VI only:** people with VI only  **No SI**: people with no HI or VI | Definition of DSI | Outcome measured | Result summary (outcome in DSI group compared to other group) |
| --- | --- | --- | --- | --- | --- | --- | --- | --- | --- | --- |
| Assi, 2020 | USA, North America | High | Cross sectional | Population | All ages | 10,783 (2,154) | No DSI | V/H: self-report single question Likert | Activities of daily living | Worse |
| Assi, 2021 | USA, North America | High | Cross sectional | Population | Older adults (65+) | 7,124 (293) | No SI; Single impairment | V/H: self-reported single question binary | Self care activities; household activities | Worse |
| Beall, 1986 | Nepal, South Asia | Low to middle | Cross sectional | Population | Older men (50+) | 117 (not stated) | HI only; VI only | V: VA <=6/60 better eye H: >=60dB; frequencies/average NS; ear NS | Activities of daily living | Worse |
| Bouscaren, 2019 | France, Western Europe | High | Cross sectional | Population | Older adults (75+) | 4,010 (not stated; 1.7%) | No SI | V/H: self-report single binary question | Activities of daily living | Worse |
| Brennan, 2005  Brennan 2006 | USA, North America | High | Prospective cohort | Population | Older adults (70+) | 5,151 (390) | No SI; HI only; VI only | V/H: self-report single categorical question | Activities of daily living | Worse |
| Campbell, 1999 | USA, North America | High | Cross sectional | Population | Older adults (65+) | 8,767 (675) | No SI | V/H: self-report multiple question | Activities of daily living; Participation | Worse |
| Chou, 2004 | Hong Kong, Southeast Asia, East Asia, and Oceania | High | Cross sectional | Population | Older adults (60+) | 2,003 (131) | No DSI | V/H: self-report single categorical question | Activities of daily living | Worse |
| Cimarolli, 2014 | USA, North America | High | Cross sectional | Population | Older adults (95+) | 119 (44) | No DSI | V/H: self-report single question with Likert scale | Activities of daily living | Worse |
| Cimarolli, 2018 | USA, North America | High | Cross sectional | Population | Older adults (95+) | 119 (not stated) | No DSI | V/H: self-report single question with Likert scale | Activities of daily living | Worse |
| Clark, 1999 | Australia, Australasia | High | Cross sectional | Population | Older adults (70+) | 1,052 (102) | No SI; HI only; VI only | V: <6/12 eye not specified H: >40dB PTA better ear | Activities of daily living;  Social participation | No difference |
| Crews, 2004 | USA, North America | High | Prospective cohort | Population | Older adults (70+) | 9,447 (779) | No SI | V/H: self-report single binary question | Activities of daily living | Worse |
| Crowe, 2018 | Denmark, Western Europe | High | Cross sectional | Register | Older adults (50+) | 513 (513) | Compared severity of loss (vision and hearing separately) | V/H: not specified | Communication participation | Varied |
| Dalby, 2009 | Canada, North America | High | Cross sectional | Register | All ages | 182 (182) | No comparator group | V/H: self-report multiple question | Communication | No comparator group |
| Deepthi, 2012 | India, South Asia | Low to middle | Cross sectional | Population | Older adults (60+) | 257 (257) | No comparator group | V: <6/18 better eye H: >25dB PTA better ear | Activities of daily living | No comparator group |
| Ehn, 2018 | Sweden, Western Europe | High | Case control | Register | Adults (18+)) | 47 (47) | No SI (reference group from the population) | V: VA chart used but definition not clear H: Based on PTA but definition not clear | Activities of daily living; social participation | Worse |
| Figueiredo, 2013 | Brazil, Latin America | Upper middle | Case series | Clinic | 20-57 years | 11 (11) | No comparator group | V/H: Clinical diagnosis of Ushers syndrome | Communication; work and leisure activities | No comparator group |
| Fletcher, 2013 | Canada, North America | High | Qualitative | Population | 44-88 years | 7 (7) | No comparator group | V/H: not specified | Activities of daily living; communication; independence; self-regulation (goal pursuit) | No comparator group |
| Fuller, 2018 | USA, North America | High | Cross sectional | Population | Older adults (45+) | 7,210,535 (not stated) | No SI; HI only; VI only | V/H: self-report single binary question | Activities of daily living; Independence | Worse |
| Gopinath, 2017 | Australia, Australasia | High | Prospective cohort | Population | Older adults (55+) | 2,956 (544) | No SI | V: <6/12 better eye H: >25dB in better ear | Retirement | No difference |
| Grue, 2009 | Multiple, Western Europe | High | Cross sectional | Clinic | Older adults (75+) | 770 (155) | No DSI | V/H: self-report single categorical question | Activities of daily living | No difference |
| Guthrie, 2018 | Canada, North America | High | Cross sectional | Other | Older adults (65+) | 402,402 (11,940) | No SI; HI only; VI only | V/H: self-report single categorical question | Activities of daily living | Worse |
| Haanes, 2014 | Norway, Western Europe | High | Cross sectional | Care home | Older adults (80+) | 93 (26) | No comparator group | V: <=6/15 better eye H: >40dB PTA better ear | Activities of daily living; Independence | No comparator group |
| Hajek, 2020 | Germany, Western Europe | High | Prospective cohort | Population | Older adults (40+) | 5,138 (815) | No DSI | V/H: self-reported single question binary | Social isolation | Worse |
| Harada, 2008 | Japan, Asia Pacific | High | Cross sectional | Population | Older adults (65+) | 843 (82) | No SI | V: <6/12 better eye H: >30dB at 1k better ear | Activities of daily living | Worse |
| Heine, 2019 | Australia, Australasia | High | Retrospective cohort | Population | Older adults (65+) | 1,000 (110 in 1994; 50 in 2004) | No SI | V/H: self-report single question categorical | Activities of daily living; Social participation; Independence | Worse |
| Heine, 2019 | China, Southeast Asia, East Asia, and Oceania | Upper middle | Cross sectional | Clinic | (Adults (18+)) | 8,268 (4,298) | No DSI | V/H: self-report single question with Likert scale | Activities of daily living | Worse |
| Heyl, 2012 | Germany, Western Europe | High | Cross sectional | Population | Older adults (75+) | 430 (43) | No SI; HI only; VIonly | V: <6/12 better eye H: >=35dB PTA in better ear | Activities of daily living | Worse |
| Jaiswal, 2019 | Iran, North Africa and Middle East | Low to middle | Qualitative | Population | (Adults (18+)) | 16 (16) | No comparator group | V/H: Formal diagnosis of deafblindness (method not specified) | Social participation;  Independence; Self-regulation (goal pursuit) | No comparator group |
| Keller, 1999 | USA, North America | High | Cross sectional | Clinic | Older adults (55+) | 576 (75) | No SI | Vision: <=20/70 in better eye (near vision) Hearing: Fail whisper voice test | Activities of daily living | Worse |
| Kwon, 2015 | South Korea, Asia Pacific | High | Cross sectional | Population | Older adults (60+) | 5,260 (268) | No SI | V: <6/18 better eye H: >40dB PTA better ear | Activities of daily living | Worse |
| Lach, 2019 | Not specified, Not specified | Unknown | Cross sectional | Care home | Older adults (50+) | 225 (67) | No DSI | V: <6/15; eye not specified H: >=40dB PTA better ear | Social participation | Worse |
| Lee, 2005 | USA, North America | High | Prospective cohort | Population | Older adults (45+) | 60,997 (1,433) | No SI | V/H: self-report single binary question | Activities of daily living | Worse |
| Lehane, 2018 | Denmark, Western Europe | High | Cross sectional | Population | (Adults (18+)) | 316 (183) | HI only; VI only | V: <6/9 eye not specified H: >=26 db PTA in better ear | Communication | Only reported in the DSI group |
| Liljas, 2018 | England, UK, Western Europe | High | Prospective cohort | Population | Older adults (50+) | 4,621 (179) | No SI | V/H: self-report single question with Likert scale | Education;  Wealth | Worse |
| Liu, 2015 | USA, North America | High | Prospective cohort | Population | Older adults (65+) | 3,871 (183) | No SI | V: self-report single binary question H: self-report single categorical question | Activities of daily living | Worse |
| Lupsakko, 2002 | Finland, Western Europe | High | Cross sectional | Population | Older adults (75+) | 470 (33) | No SI | V: <6/15 better eye H: self-report single question binary | Activities of daily living | Worse |
| Mick, 2018 | Canada, North America | High | Cross sectional | Population | 45-85 years | 21,241 (not stated) | No DSI | V/H: self-report single question with Likert scale | Social participation | Worse |
| Moller, 2003 | Sweden, Western Europe | High | Cross sectional | Secondary analysis | Only adults (Adults (18+)) | 27 (27) | No comparator group | V/H: Diagnosis of deafblindness (method not specified) | Participation | No comparator group |
| Mudie, 2018 | USA, North America | High | Cross sectional | Clinic | Older adults (50+) | 220 (42) | No SI; HI only; VI only | V: Mean deviation on visual field testing worse than -5dB better eye H: >25dB better ear | Activities of daily living; social participation | Worse |
| Mueller-Schotte, 2018 | Netherlands, Western Europe | High | Prospective cohort | Population | Older adults (60+) | 9,319 (1,693) | No SI | V/H: self-report single binary question | Activities of daily living | Worse |
| Petrovsky, 2019 | USA, North America | High | Secondary analysis | Care home | Older adults (65+) | 213 (81) | HI only; VI only | V: <=6/15 in better eye H: >40dB at 1kHz in better ear | Social participation | No difference |
| Phua, 2022 | Singapore | High | Cross sectional | Population | Older adults (60+) | 4,077 (523) | No DSI | V/H: self-report single question Likert | Social participation | Worse |
| Raina, 2004 | Canada, North America | High | Cross sectional | Population | Older adults (55+) | 16,613 (not stated) | HI only; VI only | V/H: self-report single binary question | Activities of daily living | Varied |
| Reuben, 1999 | USA, North America | High | Prospective cohort | Population | Older adults (55+) | 5,646 (36) | No SI | V: 6/12 better eye H: >40dB at 1 or/and 2kHz in better ear | Activities of daily living | Worse |
| Roets-Merken, 2017 | Netherlands, Western Europe | High | Qualitative | Care home | 82-98 years | 47 (47) | No comparator group | V:best-corrected visual acuity of <=0.3 diopter or with a visual field of <30 H: >40dB PTA better ear | Participation; Independence | No comparator group |
| Shakarchi, 2020 | USA. North America | High | Cross sectional | Population | Older adults (50+) | 13,092 (1,061) | No DSI; no SI; VI only; HI only | V/H: self-report single question Likert | Every day discrimination | Worse |
| Simning, 2018 | USA, North America | High | Prospective cohort | Population | Older adults (65+) | 7,507 (125) | No SI | V/H: self-report single binary question | Activities of daily living | Worse |
| Soto-Perez-de-Celis, 2018 | USA, North America | High | Cross sectional | Clinic | Older adults (65+) | 750 (55) | No SI | V/H: self-report single categorical question | Activities of daily living | Worse |
| Tareque, 2019 | Singapore, Asia Pacific | High | Prospective cohort | Population | Older adults (60+) | 3,452 (401) | No SI | V/H: self-report single question with Likert scale | Activities of daily living | Worse |
| Teh, 2006 | Singapore, Asia Pacific | High | Retrospective cohort | Clinic | Older adults (50+) | 112 (36) | No DSI | V: self-report single question binary H: Fail whisper voice test | Activities of daily living | Worse |
| Tinetti, 1995 | USA, North America | High | Prospective cohort | Population | Older adults (70+) | 927 (11) | No SI | V: >50% impaired on VA chart (unclear) H: Fail whisper voice test (> 5 words missed) | Activities of daily living | No difference |
| Viljanen, 2014 | Multiple, Western Europe | High | Cross sectional | Population | Older adults (50+) | 27,536 (not stated; 4.0%) | No SI | V/H: self-report single categorical question | Social participation | Worse |
| Wahl, 2013 | Germany, Western Europe | High | Cross sectional | Clinic | 75-94 | 430 (43) | No SI | V: <6/18 worse eye H: >=35dB PTA in better ear | Activities of daily living; social participation; self-regulation (goal pursuit) | Varied |
