# Supplementary material for: Prevalence and impact of combined vision and hearing (dual sensory) impairment: A scoping review
Source: PLOS Glob Public Health. 2023 May 16;3(5):e0001905. doi: 10.1371/journal.pgph.0001905 (PMC10187940; doi:10.1371/journal.pgph.0001905)
Supplement: S4 Table — (DOCX) [file pgph.0001905.s006.docx]

**S4 Table:** Reports measuring physical health outcomes for people with dual sensory impairment (DSI)

| Study author, year | Country, Region | Country income group (at time of publication) | Study design | Study setting/recruitment | Age group (years) | Sample size (n DSI) | Comparator group  **No DSI:** people with HI only, VI only, or neither HI nor VI  **Single impairment:** people with HI only or VI only  **HI only:** people with HI only  **VI only:** people with VI only  **No SI**: people with no HI or VI | Definition of DSI | Measured outcome | Outcome in DSI group compared to other group |
| --- | --- | --- | --- | --- | --- | --- | --- | --- | --- | --- |
| Appollonio, 1995 | Italy, Western Europe | High | Prospective cohort | Population | Older adults (70-75 years) | 1,303 (8) | No SI; HI only; VI only | V: <=6/15 in better eye H: Fail whisper voice test | Mortality | Worse |
| Crews, 2004 | USA, North America | High | Prospective cohort | Population | Older adults (70+) | 9,447 (779) | No SI | V/H: self-report binary question | General health | Worse |
| Crews, 2017 | USA, North America | High | Cross sectional | Population | Older adults (65+) | 36,110 (not stated) | No VI | V: self-report binary question H: self-report single question | General health | Worse |
| Dalby, 2009 | Canada, North America | High | Cross sectional | Register | All ages | 182 (182) | No comparator group | V/H: self-report multiple question | General health | No comparator |
| Dammeyer, 2010 | Denmark, Western Europe | High | Cross sectional | Population | (Adults (18+)) | 123 (123) | No comparator group | V: <=6/60 better eye H: 3FA (500,1000,2000) >=80dB; ear not specified | General health | No comparator |
| Davidson, 2019 | Canada, North America | High | Cross sectional | Care home | Older adults (65+) | 352,656 (72,188) | No DSI | V/H: self-report single categorical question | Physical functioning | Worse |
| Deardorff, 2019 | USA, North America | High | Prospective cohort | Population | Older adults (65+) | 24,009 (4,375) | No SI | V/H: self-report single categorical question | Health care costs | Worse |
| Deardorff, 2020 | USA, North America | High | Retrospective cohort | Population | Older adults (65+) | 15,999 (NS) | HI only; VI only | V: self-report single question Likert  H: self-report multiple questions | Hospitalisation | Reported only in DSI group |
| Ehn, 2018 | Sweden, Western Europe | High | Case control | Register | Only adults (Adults (18+)) | 47 (47) | No SI (reference group from the population) | V: VA chart used but definition not clear H: Based on PTA but definition not clear | General health | Worse |
| Fisher, 2014 | Iceland, Western Europe | High | Prospective cohort | Population | 67-98 years | 4,926 (343) | No SI | V: <6/15 eye not specified H: >40dB at 1 or/and 2kHz in better ear | Mortality | Varied |
| Fisher, 2016 | USA, North America | High | Cross sectional | Population | Older adults (50+) | 6,654 (316) | No comparator group | V/H: self-report single categorical question | Physical functioning | No comparator |
| Gopinath, 2013 | Australia, Australasia | High | Prospective cohort | Population | Older adults (55+) | 2,812 (947) | No DSI | V: <6/12 better eye H: >25dB PTA better ear | General health;  Mortality | Worse |
| Gopinath, 2014 | Australia, Australasia | High | Prospective cohort | Population | Older adults (55+) | 2,443 (95) | No SI | V: <6/12 better eye H: >25dB PTA better ear | Health behaviours | Worse |
| Gopinath, 2016 | Australia, Australasia | High | Prospective cohort | Population | Older adults (55+) | 1,478 (334) | No SI | V: <6/12 better eye H: >25dB PTA better ear | Physical functioning | Varied |
| Gopinath, 2021 | Australia, Australasia | High | Prospective cohort | Population | Older adults (55+) | 1,085 | No SI | V: <6/12 better eye H: >25dB PTA better ear | Mortality;, successful ageing | Same |
| Graue-Hernández, 2019 | Mexico, Latin America and the Caribbean | Upper middle | Cross sectional | Population | Older adults (50+) | 1,511 (66) | No DSI | V/H: self-report multiple question | General health | Worse |
| Green, 2013 | USA, North America | High | Retrospective cohort | Population | Older adults (70+) | 2,000 (61) | No SI | V: <6/12 eye not specified H: self-report binary | Health behaviours | Varied |
| Grue, 2008 | Norway, Western Europe | High | Cross sectional | Clinic | Older adults (65+) | 332 (not stated; 30.1%) | No comparator group | V: <6/7.5 better eye H: >=30dB PTA in better ear | Physical functioning | No comparator |
| Grue, 2009 | Multiple, Western Europe | High | Cross sectional | Clinic | Older adults (75+) | 770 (155) | No DSI | V/H: self-report single categorical question | Physical functioning | No difference |
| Heine, 2019 | Australia, Australasia | High | Retrospective cohort | Population | Older adults (65+) | 1,000 (110 in 1994; 50 in 2004) | No SI | V/H: self-report single question categorical | General health | Worse |
| Heyl, 2012 | Germany, Western Europe | High | Cross sectional | Population | Older adults (75+) | 430 (43) | No SI; HI only; VI only | V: <6/12 better eye H: >=35dB PTA in better ear | General health | Varied |
| Huddle, 2016 | USA, North America | High | Cross sectional | Population | Older adults (70+) | 1,669 (291) | No SI | V: self-report single question binary H: >=25dB PTA in better ear | General health; health behaviours; hospitalisation | Varied |
| Jin, 2010 | Canada, North America | High | Qualitative | Deaf Blind Association of Ontario | 37-76 years | 7 (7) | No comparator group | Not specified; participants were deafblind | Oral health | No comparator |
| Jung, 2022 | South Korea, Asia Pacific | High | Prospective cohort | Register | 30+ | 77,1128 (8,720) | No DSI | V: <6/12 better eye  H: >40dB PTA at least one ear | Stroke; mortality | Worse |
| Kiely, 2016 | Australia, Australasia | High | Prospective cohort | Population | Older adults (50+) | 4,160 (not stated) | HI only ; VI only | V: <6/12 eye not specified H: >25dB PTA better ear | Mortality | Worse |
| Kulmala, 2009 | Finland, Western Europe | High | Prospective cohort | Population | 63-76 years | 428 (74) | People with good vision | V: <6/60 eye not specified H: >=21 dB PTA in better ear | Physical functioning | Worse |
| Lach, 2019 | Not specified, Not specified | Unknown | Cross sectional | Care home | Older adults (50+) | 225 (67) | No DSI | V: <6/15; eye not specified H: >=40dB PTA better ear | General health; Physical functioning | No difference |
| Lam, 2006 | USA, North America | High | Retrospective cohort | Clinic | Older adults (Adults (18+)) | 116,796 (1,461) | No SI | V/H: self-report binary question | Mortality | Varied |
| Lee, 2007 | USA, North America | High | Retrospective cohort | Population | Adults (18+) | 116,796 (1,461) | No SI | V/H: self-report binary question | Mortality | Varied |
| Lehane, 2017 | Denmark, Western Europe | High | Cross sectional | Register | Older adults (50+) | 45 (45) | Population mean | V/H: Registered as deafblind according to Nordic definition | Sexual health | Worse |
| Liljas, 2018 | England, UK, Western Europe | High | Prospective cohort | Population | Older adults (50+) | 4,621 (179) | No SI | V/H: self-report single question with Likert scale | General health; Physical functioning | Worse |
| Lin, 2004 | USA, North America | High | Cross sectional | Population | Older adults (65+) | 6,112 (not stated) | No DSI | V: <6/12 in better eye H: >=40dB at 2kHz in better ear | Physical functioning | Worse |
| Liu, 2015 | USA, North America | High | Prospective cohort | Population | Older adults (65+) | 3,871 (183) | No SI | V: self-report single binary question H: self-report single categorical question | General health; mortality | Worse |
| Loprinzi, 2013 | USA, North America | High | Cross sectional | Population | 20-84 years | 1,445 (29) | No SI | V: <6/12 better eye H: >25dB PTA better ear | Health behaviours | Worse |
| McDonnall , 2011 | USA, North America | High | Prospective cohort | Population | Older adults (50+) | 2,688 (not stated) | No SI | V/H: self-report single question with Likert scale | Health behaviours | Worse |
| Mitoku, 2016 | Japan, Asia Pacific | High | Prospective cohort | Population | Older adults (50+) | 1,754 (320) | No SI | V: VA chart definition not clear H: self-report single categorical question | Mortality | Worse |
| Miyawaki, 2019 | Japan, Asia Pacific | High | Retrospective cohort | Population | 40-69 years | 9,522 (86) | No comparator group | V/H: self-report single question categorical | Mortality | No comparator |
| Mudie, 2018 | USA, North America | High | Cross sectional | Clinic | Older adults (50+) | 220 (42) | No SI; HI only; VI only | V: Mean deviation on visual field testing worse than -5dB better eye H: >25dB better ear | Physical functioning | No difference |
| Ogliari, 2021 | Multiple | High | Prospective cohort | Population | Older adults (50+) | 50,986 (3853) | No DSI | V/H: self-report single question Likert | Physical functioning | Worse |
| Olakunde, 2020 | USA, North America | High | Cross sectional | Population | Adults (18+) | 468,303 (1349) | No DSI | V/H: self report, single binary question | Sexual health | Same |
| Reed, 2020 | USA, North America | High | Cross sectional/ Retrospective cohort | Population | Adults (18+) | 10,748 (121) | No DSI | V/H: self-report, single question Likert | Health behaviours | Worse |
| Reuben, 1999 | USA, North America | High | Prospective cohort | Population | Older adults (55+) | 5,646 (36) | No SI | V: 6/12 better eye H: >40dB at 1 or/and 2kHz in better ear | Mortality | No difference |
| Shakarchi, 2021 | USA, North America | High | Prospective cohort | Population | Older adults (65+) | 7,648 (697) | No SI | V/H: self-report single question Likert scale | Walking speed | Worse |
| Soto-Perez-de-Celis, 2018 | USA, North America | High | Cross sectional | Clinic | Older adults (65+) | 750 (55) | No SI | V/H: self-report single categorical question | Physical functioning | No difference |
| Steinman, 2021 | USA, North America | High | Prospective cohort | Population | Older adults (65+) | 4,636 (111) | No DSI | V/H: self-report multiple questions | Mortality; self-rated health | Same |
| Sun, 2020 | China | Upper middle | Prospective cohort | Population | - | 37,076 (7,774) | No DSI | V/H: clinician judgement | Mortality | Worse |
| Tareque, 2019 | Singapore, Asia Pacific | High | Prospective cohort | Population | Older adults (60+) | 3,452 (401) | No SI | V/H: self-report single question with Likert scale | Mortality; Physical functioning | Worse |
| Teh, 2006 | Singapore, Asia Pacific | High | Retrospective cohort | Clinic | Older adults (50+) | 112 (36) | DSI | V: self-report single question binary H: Fail whisper voice test | Physical functioning | Varied |
| Tinetti, 1995 | USA, North America | High | Prospective cohort | Population | Older adults (70+) | 927 (11) | No SI | V: >50% impaired on VA chart (unclear) H: Fail whisper voice test (> 5 words missed) | Physical functioning | Worse |
| Wahl, 2013 | Germany, Western Europe | High | Cross sectional | Clinic | 75-94 | 430 (43) | No SI | V: <6/18 worse eye H: >=35dB PTA in better ear | General health | Worse |
| Williams, 2020 | Canada, North America | High | Retrospective cohort | Care home | Older adults (55+) | 371,696 (not stated) | No SI | V/H: self-report single categorical question | Physical functioning; long term care admissions | No difference |
| Yamada, 2014 | Multiple, Western Europe | High | Cross sectional | Care home | Older adults (50+) | 4,007 (1,275) | No SI | V/H: self-report single categorical question | General health | Worse |
| Yamada, 2016 | Multiple, Western Europe | High | Cross sectional | Care home | Older adults (50+) | 2,851 (88) | No SI | V/H: self-report single categorical question | Mortality | Worse |
| Zhang, 2020 | China, Asia Pacific | Upper middle | Prospective cohort | Population | Older adults (80+) | 8,788 (860) | No SI | V/H: self-report single categorical question | Mortality | Worse |
| Zhou, 2022 | China, Asia Pacific | Upper middle | Prospective cohort | Population | Older adults (45+) | 7,623 (3,163) | No DSI | V: self-report multiple questions  H: self-report single question Likert | Falls | Worse |
