# Supplementary material for: Prevalence and impact of combined vision and hearing (dual sensory) impairment: A scoping review
Source: PLOS Glob Public Health. 2023 May 16;3(5):e0001905. doi: 10.1371/journal.pgph.0001905 (PMC10187940; doi:10.1371/journal.pgph.0001905)
Supplement: S5 Table — (DOCX) [file pgph.0001905.s007.docx]

**S5 Table** : Included systematic reviews

| First author, year | Study design | Number of included studies | Outcome categories examined |
| --- | --- | --- | --- |
| Arcous, 2019 | Scoping review | 21 | Participation; psychosocial; physical |
| Ask Larsen, 2014 | Systematic review | 30 | Definitions |
| Besser, 2018 | Non-systematic review | 7 | Prevalence |
| Carvill, 2001 | Non-systematic review | Not reported | Psychosocial |
| Dammeyer, 2014 | Non-systematic review | Not reported | Psychosocial |
| Dewan, 2012 | Systematic review | 55 | Prevalence |
| Heine, 2015 | Systematic review | 42 | Participation; psychosocial |
| Heine, 2014 | Systematic review | 8 | Psychosocial |
| Jaiswal, 2018 | Scoping review | 54 | Participation |
| Lehane, 2017 | Integrative review | 24 | Psychosocial |
| Schneider, 2011 | Non-systematic review | 40 | Psychosocial; participation; physical |
| Simcock, 2017 | Systematic review | 28 | Psychosocial |
| Simcock, 2017 | Systematic review | 24 | Participation |
| Tseng, 2018 | Systematic review | 23 | Psychosocial |
