# Supplementary material for: Prevalence and impact of combined vision and hearing (dual sensory) impairment: A scoping review
Source: PLOS Glob Public Health. 2023 May 16;3(5):e0001905. doi: 10.1371/journal.pgph.0001905 (PMC10187940; doi:10.1371/journal.pgph.0001905)
Supplement: S6 Table — (DOCX) [file pgph.0001905.s008.docx]

**S6 Table: Included studies (n=197)**

|  | **Authors** | **Title** | **Year** | **Journal** |
| --- | --- | --- | --- | --- |
| 1 | Amini, Reza; Haghani, Hamid; Masoumi, Mehdi | Quality of life in the Iranian Blind War Survivors in 2007: a cross-sectional study. | 2010 | BMC international health and human rights |
| 2 | Appollonio, I; Carabellese, C; Magni, E; Frattola, L; Trabucchi, M | Sensory impairments and mortality in an elderly community population: a six-year follow-up study. | 1995 | Age and ageing |
| 3 | Arcous, Marine; Putois, Olivier; Dalle-Nazebi, Sophie; Kerbourch, Sylvain; Cariou, Anaelle; Ben Aissa, Ines; Marlin, Sandrine; Potier, Remy | Psychosocial determinants associated with quality of life in people with usher syndrome. A scoping review. | 2019 | Disability and rehabilitation |
| 4 | Armstrong T.W.; Surya S.; Elliott T.R.; Brossart D.F.; Burdine J.N. | Depression and health-related quality of life among persons with sensory disabilities in a health professional shortage area | 2016 | Rehabilitation Psychology |
| 5 | Armstrong, Nicole M; Wang, Hang; E, Jian-Yu; Lin, Frank R; Abraham, Alison G; Ramulu, Pradeep; Resnick, Susan M; Tian, Qu; Simonsick, Eleanor; Gross, Alden L; Schrack, Jennifer A; Ferrucci, Luigi; Agrawal, Yuri | Patterns of Prevalence of Multiple Sensory Impairments among Community-Dwelling Older Adults. | 2021 | The journals of gerontology. Series A, Biological sciences and medical sciences |
| 6 | Ask Larsen, Flemming; Damen, Saskia | Definitions of deafblindness and congenital deafblindness. | 2014 | Research in developmental disabilities |
| 7 | Assi, Lama; Ehrlich, Joshua R; Zhou, Yunshu; Huang, Alison; Kasper, Judith; Lin, Frank R; McKee, Michael M; Reed, Nicholas S; Swenor, Bonnielin K; Deal, Jennifer A | Self-reported dual sensory impairment, dementia, and functional limitations in Medicare beneficiaries. | 2021 | Journal of the American Geriatrics Society |
| 8 | Assi, Lama; Shakarchi, Ahmed F; Sheehan, Orla C; Deal, Jennifer A; Swenor, Bonnielin K; Reed, Nicholas S | Assessment of Sensory Impairment and Health Care Satisfaction Among Medicare Beneficiaries. | 2020 | JAMA network open |
| 9 | Beall, C M; Goldstein, M C | Age differences in sensory and cognitive function in elderly Nepalese. | 1986 | Journal of gerontology |
| 10 | Bergman, Birgitta; Rosenhall, Ulf | Vision and hearing in old age | 2001 | Scandinavian Audiology |
| 11 | Besser, Jana; Stropahl, Maren; Urry, Emily; Launer, Stefan | Comorbidities of hearing loss and the implications of multimorbidity for audiological care. | 2018 | Hearing research |
| 12 | Bodsworth, Sarah M; Clare, Isabel CH; Simblett, Sara K; Deafblind UK | Deafblindness and mental health: Psychological distress and unmet need among adults with dual sensory impairment | 2011 | British Journal of Visual Impairment |
| 13 | Bouscaren, N; Yildiz, H; Dartois, L; Vercambre, M N; Boutron-Ruault, M C | Decline in Instrumental Activities of Daily Living over 4-Year: The Association with Hearing, Visual and Dual Sensory Impairments among Non-Institutionalized Women. | 2019 | The journal of nutrition, health & aging |
| 14 | Brennan, Mark; Horowitz, Amy; Su, Ya-Ping | Dual sensory loss and its impact on everyday competence. | 2005 | The Gerontologist |
| 15 | Brennan, Mark; Su, Ya-ping; Horowitz, Amy | Longitudinal associations between dual sensory impairment and everyday competence among older adults. | 2006 | Journal of rehabilitation research and development |
| 16 | Bright, Tess; McCormick, Ian; Phiri, Mwanaisha; Mulwafu, Wakisa; Burton, Matthew; Polack, Sarah; Mactaggart, Islay; Yip, Jennifer L Y; Swanepoel, De Wet; Kuper, Hannah | Rationale and feasibility of a combined rapid assessment of avoidable blindness and hearing loss protocol. | 2020 | PloS one |
| 17 | Byeon, Gihwan; Oh, Gyu Han; Jhoo, Jin Hyeong; Jang, Jae-Won; Bae, Jong Bin; Han, Ji Won; et al. | Dual Sensory Impairment and Cognitive Impairment in the Korean Longitudinal Elderly Cohort. | 2021 | Neurology |
| 18 | Caban, Alberto J; Lee, David J; Gomez-Marin, Orlando; Lam, Byron L; Zheng, D Diane | Prevalence of concurrent hearing and visual impairment in US adults: The National Health Interview Survey, 1997-2002. | 2005 | American journal of public health |
| 19 | Cacchione, Pamela Z; Culp, Kennith; Dyck, Mary J; Laing, Joan | Risk for acute confusion in sensory-impaired, rural, long-term-care elders. | 2003 | Clinical nursing research |
| 20 | Campbell, V A; Crews, J E; Moriarty, D G; Zack, M M; Blackman, D K | Surveillance for sensory impairment, activity limitation, and health-related quality of life among older adults--United States, 1993-1997. | 1999 | MMWR. CDC surveillance summaries : Morbidity and mortality weekly report. CDC surveillance summaries |
| 21 | Capella-McDonnall, Michele E | The effects of single and dual sensory loss on symptoms of depression in the elderly. | 2005 | International journal of geriatric psychiatry |
| 22 | Carvill, S | Sensory impairments, intellectual disability and psychiatry. | 2001 | Journal of intellectual disability research : JIDR |
| 23 | Chia, Ee-Munn; Mitchell, Paul; Rochtchina, Elena; Foran, Suriya; Golding, Maryanne; Wang, Jie Jin | Association between vision and hearing impairments and their combined effects on quality of life. | 2006 | Archives of ophthalmology |
| 24 | Chou, Kee-Lee | Combined effect of vision and hearing impairment on depression in older adults: evidence from the English Longitudinal Study of Ageing. | 2008 | Journal of affective disorders |
| 25 | Chou, Kee-Lee; Chi, Iris | Combined effect of vision and hearing impairment on depression in elderly Chinese. | 2004 | International journal of geriatric psychiatry |
| 26 | Cimarolli, Verena R; Jopp, Daniela S | Sensory impairments and their associations with functional disability in a sample of the oldest-old. | 2014 | Quality of life research |
| 27 | Cimarolli, Verena R; Jopp, Daniela S; Boerner, Kathrin; Minahan, Jillian | Depressive symptoms in the oldest-old: The role of sensory impairments. | 2018 | Archives of gerontology and geriatrics |
| 28 | Clark, Michael S; Bond, Malcolm J; Sanchez, Linnett | The effect of sensory impairment on the lifestyle activities of older people | 1999 | Australasian Journal on Ageing |
| 29 | Cosh, S; von Hanno, T; Helmer, C; Bertelsen, G; Delcourt, C; Schirmer, H; SENSE-Cog Group | The association amongst visual, hearing, and dual sensory loss with depression and anxiety over 6 years: The Tromso Study. | 2018 | International journal of geriatric psychiatry |
| 30 | Crews, John E; Campbell, Vincent A | Vision impairment and hearing loss among community-dwelling older Americans: implications for health and functioning. | 2004 | American journal of public health |
| 31 | Crews, John E; Chou, Chiu-Fang; Sekar, Swathi; Saaddine, Jinan B | The Prevalence of Chronic Conditions and Poor Health Among People With and Without Vision Impairment, Aged >=65 Years, 2010-2014. | 2017 | American journal of ophthalmology |
| 32 | Crowe K, Hovaldt HB, Dammeyer J. | Communication participation in older adults with dual sensory loss. | 2018 | Speech Lang Hear. |
| 33 | Dalby, Dawn M; Hirdes, John P; Stolee, Paul; Strong, J Graham; Poss, Jeff; Tjam, Erin Y; Bowman, Lindsay; Ashworth, Melody | Characteristics of individuals with congenital and acquired deaf-blindness | 2009 | Journal of Visual Impairment & Blindness |
| 34 | Dammeyer, Jesper | Congenital rubella syndrome and delayed manifestations. | 2010 | International journal of pediatric otorhinolaryngology |
| 35 | Dammeyer, Jesper | Deafblindness: a review of the literature. | 2014 | Scandinavian journal of public health |
| 36 | Dammeyer, Jesper | Characteristics of a Danish population of adults with acquired deafblindness receiving rehabilitation services | 2013 | British Journal of Visual Impairment |
| 37 | Dammeyer, Jesper | Symptoms of autism among children with congenital deafblindness. | 2014 | Journal of autism and developmental disorders |
| 38 | Dammeyer, Jesper | Prevalence and aetiology of congenitally deafblind people in Denmark. | 2010 | International journal of audiology |
| 39 | Dammeyer, Jesper | Mental and behavioral disorders among people with congenital deafblindness. | 2011 | Research in developmental disabilities |
| 40 | Davidson, Jacob G S; Guthrie, Dawn M | Older Adults With a Combination of Vision and Hearing Impairment Experience Higher Rates of Cognitive Impairment, Functional Dependence, and Worse Outcomes Across a Set of Quality Indicators. | 2019 | Journal of aging and health |
| 41 | Dawes, Piers; Dickinson, Christine; Emsley, Richard; Bishop, Paul N; Cruickshanks, Karen J; Edmondson-Jones, Mark; et al | Vision impairment and dual sensory problems in middle age. | 2014 | Ophthalmic & physiological optics |
| 42 | de la Fuente, Javier; Hjelmborg, Jacob; Wod, Mette; de la Torre-Luque, Alejandro; Caballero, Francisco Felix; Christensen, Kaare; Ayuso-Mateos, Jose Luis | Longitudinal Associations of Sensory and Cognitive Functioning: A Structural Equation Modeling Approach. | 2019 | The journals of gerontology. Series B, Psychological sciences and social sciences |
| 43 | Deardorff, William J; Sloane, Richard J; Pavon, Juliessa M; Hastings, Susan N; Whitson, Heather E | Hospitalization Risk Among Older Adults with Sensory Impairments: Development of a Prognostic Model. | 2020 | Journal of the American Geriatrics Society |
| 44 | Deardorff, William James; Liu, Phillip L; Sloane, Richard; Van Houtven, Courtney; Pieper, Carl F; Hastings, Susan Nicole; Cohen, Harvey J; Whitson, Heather E | Association of Sensory and Cognitive Impairment With Healthcare Utilization and Cost in Older Adults. | 2019 | Journal of the American Geriatrics Society |
| 45 | Dewan, Pooja; Gupta, Piyush | Burden of Congenital Rubella Syndrome (CRS) in India: a systematic review. | 2012 | Indian pediatrics |
| 46 | Dupuis, Kate; Pichora-Fuller, M Kathleen; Chasteen, Alison L; Marchuk, Veronica; Singh, Gurjit; Smith, Sherri L | Effects of hearing and vision impairments on the Montreal Cognitive Assessment. | 2015 | Neuropsychology, development, and cognition. Section B, Aging, neuropsychology and cognition |
| 47 | Ehn, Mattias; Wahlqvist, Moa; Danermark, Berth; Dahlstrom, Orjan; Moller, Claes | Health, work, social trust, and financial situation in persons with Usher syndrome type 1. | 2018 | Work (Reading, Mass.) |
| 48 | Figueiredo, Marilia Zannon de Andrade; Chiari, Brasilia Maria; Goulart, Barbara Niegia Garcia de | Communication in deafblind adults with Usher syndrome: retrospective observational study. | 2013 | CoDAS |
| 49 | Fischer, Mary E; Cruickshanks, Karen J; Klein, Barbara E K; Klein, Ronald; Schubert, Carla R; Wiley, Terry L | Multiple sensory impairment and quality of life. | 2009 | Ophthalmic epidemiology |
| 50 | Fisher, Diana E; Ward, Michael M; Hoffman, Howard J; Li, Chuan-Ming; Cotch, Mary Frances | Impact of Sensory Impairments on Functional Disability in Adults With Arthritis. | 2016 | American journal of preventive medicine |
| 51 | Fisher, Diana; Li, Chuan-Ming; Chiu, May S; Themann, Christa L; Petersen, Hannes; Jonasson, Fribert; et al. | Impairments in hearing and vision impact on mortality in older people: the AGES-Reykjavik Study. | 2014 | Age and ageing |
| 52 | Fletcher, Paula C; Guthrie, Dawn M | The lived experiences of individuals with acquired deafblindness: Challenges and the future | 2013 | ?? |
| 53 | Forbes W.F.; Hayward L.M.; Agwani N. | Factors associated with the prevalence of various self-reported impairments among older people residing in the community | 1991 | Canadian Journal of Public Health |
| 54 | Fuller, Spencer D; Mudie, Lucy I; Siordia, Carlos; Swenor, Bonnielin K; Friedman, David S | Nationwide Prevalence of Self-Reported Serious Sensory Impairments and Their Associations with Self-Reported Cognitive and Functional Difficulties. | 2018 | Ophthalmology |
| 55 | Gadkaree, S. K.; Sun, D. Q.; Li, C.; Lin, F. R.; Ferrucci, L.; Simonsick, E. M.; Agrawal, Y. | Does Sensory Function Decline Independently or Concomitantly with Age? Data from the Baltimore Longitudinal Study of Aging | 2016 | Journal of aging research |
| 56 | Ge, Shaoqing; McConnell, Eleanor S; Wu, Bei; Pan, Wei; Dong, XinQi; Plassman, Brenda L | Longitudinal Association Between Hearing Loss, Vision Loss, Dual Sensory Loss, and Cognitive Decline. | 2021 | Journal of the American Geriatrics Society |
| 57 | Glatz, Marlene; Riedl, Regina; Glatz, Wilfried; Schneider, Mona; Wedrich, Andreas; Bolz, Matthias; Strauss, Rupert W | Blindness and visual impairment in Central Europe. | 2022 | PloS one |
| 58 | Gopinath, B; Schneider, J; Flood, V M; McMahon, C M; Burlutsky, G; Leeder, S R; Mitchell, P | Association between diet quality with concurrent vision and hearing impairment in older adults. | 2014 | The journal of nutrition, health & aging |
| 59 | Gopinath, Bamini; Liew, Gerald; Burlutsky, George; McMahon, Catherine M; Mitchell, Paul | Association between vision and hearing impairment and successful aging over five years. | 2021 | Maturitas |
| 60 | Gopinath, Bamini; Liew, Gerald; Burlutsky, George; McMahon, Catherine M; Mitchell, Paul | Visual and hearing impairment and retirement in older adults: A population-based cohort study. | 2017 | Maturitas |
| 61 | Gopinath, Bamini; McMahon, Catherine M; Burlutsky, George; Mitchell, Paul | Hearing and vision impairment and the 5-year incidence of falls in older adults. | 2016 | Age and ageing |
| 62 | Gopinath, Bamini; Schneider, Julie; McMahon, Catherine M; Burlutsky, George; Leeder, Stephen R; Mitchell, Paul | Dual sensory impairment in older adults increases the risk of mortality: a population-based study. | 2013 | PloS one |
| 63 | Graue-Hernandez, Enrique O; Gomez-Dantes, Hector; Romero-Martinez, Martin; Bravo, Gerardo; Arrieta-Camacho, Jesus; Jimenez-Corona, Aida | [Self-reported hearing loss and visual impairment in adults from Central Mexico]. | 2019 | Salud publica de Mexico |
| 64 | Green, Kimberly A; McGwin, Gerald Jr; Owsley, Cynthia | Associations between visual, hearing, and dual sensory impairments and history of motor vehicle collision involvement of older drivers. | 2013 | Journal of the American Geriatrics Society |
| 65 | Grue, Else Vengnes; Kirkevold, Marit; Ranhoff, Anette Hylen | Prevalence of vision, hearing, and combined vision and hearing impairments in patients with hip fractures. | 2009 | Journal of clinical nursing |
| 66 | Grue, Else Vengnes; Ranhoff, Anette Hylen; Noro, Anja; Finne-Soveri, Harriet; Jensdottir, Anna Birna; Ljunggren, Gunnar; Bucht, Gosta; Bjornson, Leif Jan; Jonsen, Elisabeth; Schroll, Marianne; Jonsson, Palmi V | Vision and hearing impairments and their associations with falling and loss of instrumental activities in daily living in acute hospitalized older persons in five Nordic hospitals. | 2009 | Scandinavian journal of caring sciences |
| 67 | Guthrie, Dawn M; Davidson, Jacob G S; Williams, Nicole; Campos, Jennifer; Hunter, Kathleen; Mick, Paul; Orange, Joseph B; Pichora-Fuller, M Kathleen; Phillips, Natalie A; Savundranayagam, Marie Y; Wittich, Walter | Combined impairments in vision, hearing and cognition are associated with greater levels of functional and communication difficulties than cognitive impairment alone: Analysis of interRAI data for home care and long-term care recipients in Ontario. | 2018 | PloS one |
| 68 | Guthrie, Dawn M; Declercq, Anja; Finne-Soveri, Harriet; Fries, Brant E; Hirdes, John P | The Health and Well-Being of Older Adults with Dual Sensory Impairment (DSI) in Four Countries. | 2016 | PloS one |
| 69 | Guthrie, Dawn M; ThÃ©riault, Ã‰ric R; Davidson, Jacob GS | Self-rated health, cognition, and dual sensory impairment are important predictors of depression among home care clients in Ontario | 2016 | Home Health Care Management & Practice |
| 70 | Haanes, Gro Gade; Kirkevold, Marit; Horgen, Gunnar; Hofoss, Dag; Eilertsen, Grethe | Sensory impairments in community health care: a descriptive study of hearing and vision among elderly Norwegians living at home. | 2014 | Journal of multidisciplinary healthcare |
| 71 | Haanes, Gro Gade; Roin, Asa; Petersen, Maria Skaalum | Preventive Home Visit (PHV) Screening of Hearing and Vision Among Older Adults in Torshavn, Faroe Islands: A Feasibility Study in a Small-Scale Community. | 2021 | Journal of multidisciplinary healthcare |
| 72 | Hajek, Andre; Konig, Hans-Helmut | Dual sensory impairment and psychosocial factors. Findings based on a nationally representative sample. | 2020 | Archives of gerontology and geriatrics |
| 73 | Han, J H; Lee, H J; Jung, J; Park, E-C | Effects of self-reported hearing or vision impairment on depressive symptoms: a population-based longitudinal study. | 2019 | Epidemiology and psychiatric sciences |
| 74 | Harada, Sei; Nishiwaki, Yuji; Michikawa, Takehiro; Kikuchi, Yuriko; Iwasawa, Satoko; Nakano, Makiko; Ishigami, Ai; Saito, Hideyuki; Takebayashi, Toru | Gender difference in the relationships between vision and hearing impairments and negative well-being. | 2008 | Preventive medicine |
| 75 | Harithasan, Deepashini; Mukari, Siti Zamratol-Mai Sarah; Ishak, Wan Syafira; Shahar, Suzana; Yeong, Wong Lai | The impact of sensory impairment on cognitive performance, quality of life, depression, and loneliness in older adults. | 2020 | International journal of geriatric psychiatry |
| 76 | Hartshorne, Timothy S; Heussler, Helen S; Dailor, A Nichole; Williams, George L; Papadopoulos, Dimitrios; Brandt, Kimberly K | Sleep disturbances in CHARGE syndrome: types and relationships with behavior and caregiver well-being. | 2009 | Developmental medicine and child neurology |
| 77 | Hartshorne, Timothy S; Nicholas, Jude; Grialou, Tina L; Russ, Joanna M | Executive function in CHARGE syndrome | 2007 | Child Neuropsychology |
| 78 | Heine, Chyrisse; Browning, Colette | Dual Sensory Loss in Older Adults: A Systematic Review. | 2015 | The Gerontologist |
| 79 | Heine, Chyrisse; Browning, Colette J | Mental health and dual sensory loss in older adults: a systematic review. | 2014 | Frontiers in aging neuroscience |
| 80 | Heine, Chyrisse; Browning, Colette J; Gong, Cathy Honge | Sensory Loss in China: Prevalence, Use of Aids, and Impacts on Social Participation. | 2019 | Frontiers in public health |
| 81 | Heine, Chyrisse; Gong, Cathy Honge; Browning, Colette | Dual Sensory Loss, Mental Health, and Wellbeing of Older Adults Living in China. | 2019 | Frontiers in public health |
| 82 | Heine, Chyrisse; Gong, Cathy Honge; Feldman, Susan; Browning, Colette | Older Women in Australia: Facing the Challenges of Dual Sensory Loss. | 2019 | International journal of environmental research and public health |
| 83 | Hersh M.A. | Deafblind people, stigma and the use of communication and mobility assistive devices | 2013 | Technology and Disability |
| 84 | Heyl, Vera; Wahl, Hans-Werner | Managing daily life with age-related sensory loss: cognitive resources gain in importance. | 2012 | Psychology and aging |
| 85 | Hickson, Louise; Lind, Christopher; Worrall, Linda; Yiu, Edwin; Barnett, Heather; Lovie-Kitchin, Jan | Hearing and vision in healthy older Australians: Objective and self-report measures | 1999 | Advances in Speech Language Pathology |
| 86 | Ho, Kam Chun; Fenwick, Eva K; Gupta, Preeti; Gan, Alfred; Loo, Jenny Hy; Ma, Lina; Koh, Gerald; Wong, Tien Y; Lamoureux, Ecosse L; Man, Ryan Ek | Prevalence, Associated Factors and Health-related Quality of Life of Dual Sensory Impairment in Residential Care Facilities in Singapore. | 2021 | Ophthalmic epidemiology |
| 87 | Hong, Thomas; Mitchell, Paul; Burlutsky, George; Liew, Gerald; Wang, Jie Jin | Visual Impairment, Hearing Loss and Cognitive Function in an Older Population: Longitudinal Findings from the Blue Mountains Eye Study. | 2016 | PloS one |
| 88 | Hovaldt HB, Lund R, Lehane CM, Dammeyer J | Relational strain in close social relations among older adults with dual sensory loss | 2019 | Br J Vis Impair. |
| 89 | Hovaldt, Hanna B; Crowe, Kathryn; Dammeyer, Jesper | A cross-sectional study of prevalence and correlates of self-harm and suicidal ideation in older adults with dual sensory loss. | 2022 | Disability and health journal |
| 90 | Huddle, Matthew G; Deal, Jennifer A; Swenor, Bonnielin; Genther, Dane J; Lin, Frank R | The Association of Dual Sensory Impairment with Hospitalization and Burden of Disease | 2016 | Journal of the American Geriatrics Society |
| 91 | Hwang, Phillip H; Longstreth, W T Jr; Brenowitz, Willa D; Thielke, Stephen M; Lopez, Oscar L; Francis, Courtney E; DeKosky, Steven T; Fitzpatrick, Annette L | Dual sensory impairment in older adults and risk of dementia from the GEM Study. | 2020 | Alzheimer's & dementia (Amsterdam, Netherlands) |
| 92 | Jaiswal, Atul; Aldersey, Heather M; Wittich, Walter; Mirza, Mansha; Finlayson, Marcia | Meaning and experiences of participation: a phenomenological study with persons with deafblindness in India. | 2019 | Disability and rehabilitation |
| 93 | Jaiswal, Atul; Aldersey, Heather; Wittich, Walter; Mirza, Mansha; Finlayson, Marcia | Participation experiences of people with deafblindness or dual sensory loss: A scoping review of global deafblind literature. | 2018 | PloS one |
| 94 | Jin, Elvine Y W; Daly, Blanaid | The self-reported oral health status and behaviors of adults who are deaf and blind. | 2010 | Special care in dentistry |
| 95 | Jung, Younhea; Han, Kyungdo; Lee, Ji Min; Park, Hye Yeon; Moon, Jung Il | Impact of vision and hearing impairments on risk of cardiovascular outcomes and mortality in patients with type 2 diabetes: A nationwide cohort study. | 2022 | Journal of diabetes investigation |
| 96 | Keller, B K; Morton, J L; Thomas, V S; Potter, J F | The effect of visual and hearing impairments on functional status. | 1999 | Journal of the American Geriatrics Society |
| 97 | Khandekar, Rajiv; Al Khabori, Mazin | Double disability: the hearing-impaired blind in the Sultanate of Oman. | 2004 | International journal of audiology |
| 98 | Khil, Laura; Wellmann, Jurgen; Berger, Klaus | Impact of combined sensory impairments on health-related quality of life. | 2015 | Quality of life research |
| 99 | Khurana, Maitri; Shoham, Natalie; Cooper, Claudia; Pitman, Alexandra Laura | Association between sensory impairment and suicidal ideation and attempt: a cross-sectional analysis of nationally representative English household data. | 2021 | BMJ open |
| 100 | Kiely, Kim M; Anstey, Kaarin J; Luszcz, Mary A | Dual sensory loss and depressive symptoms: the importance of hearing, daily functioning, and activity engagement. | 2013 | Frontiers in human neuroscience |
| 101 | Kiely, Kim M; Mitchell, Paul; Gopinath, Bamini; Luszcz, Mary A; Jagger, Carol; Anstey, Kaarin J | Estimating the Years Lived With and Without Age-Related Sensory Impairment. | 2016 | The journals of gerontology. Series A, Biological sciences and medical sciences |
| 102 | Kiely, Kim M; Mortby, Moyra E; Anstey, Kaarin J | Differential associations between sensory loss and neuropsychiatric symptoms in adults with and without a neurocognitive disorder. | 2018 | International psychogeriatrics |
| 103 | Killeen O.J.; Xiang X.; Powell D.; Reed N.S.; Deal J.A.; Swenor B.K.; Ehrlich J.R. | Longitudinal Associations of Self-Reported Visual, Hearing, and Dual Sensory Difficulties With Symptoms of Depression Among Older Adults in the United States | 2022 | Frontiers in Neuroscience |
| 104 | Kim, Yoonjung; Kwak, Yeunhee; Kim, Ji-Su | The association between suicide ideation and sensory impairment among elderly Koreans. | 2015 | Aging & mental health |
| 105 | Klein, R; Cruickshanks, K J; Klein, B E; Nondahl, D M; Wiley, T | Is age-related maculopathy related to hearing loss?. | 1998 | Archives of ophthalmology |
| 106 | Kulmala, Jenni; Viljanen, Anne; Sipila, Sarianna; Pajala, Satu; Parssinen, Olavi; Kauppinen, Markku; Koskenvuo, Markku; Kaprio, Jaakko; Rantanen, Taina | Poor vision accompanied with other sensory impairments as a predictor of falls in older women. | 2009 | Age and ageing |
| 107 | Kuo, Pei-Lun; Huang, Alison R; Ehrlich, Joshua R; Kasper, Judith; Lin, Frank R; McKee, Michael M; Reed, Nicholas S; Swenor, Bonnielin K; Deal, Jennifer A | Prevalence of Concurrent Functional Vision and Hearing Impairment and Association With Dementia in Community-Dwelling Medicare Beneficiaries. | 2021 | JAMA network open |
| 108 | Kwan, Rick Yiu Cho; Kwan, Chi Wai; Kor, Patrick Pui Kin; Chi, Iris | Cognitive decline, sensory impairment, and the use of audio-visual aids by long-term care facility residents. | 2022 | BMC geriatrics |
| 109 | Kwon, Hye-Jin; Kim, Ji-Su; Kim, Yoon-Jung; Kwon, Su-Jin; Yu, Jin-Na | Sensory Impairment and Health-Related Quality of Life. | 2015 | Iranian journal of public health |
| 110 | Lach, Helen W; Lozano, Alicia J; Hanlon, Alexandra L; Cacchione, Pamela Z | Fear of falling in sensory impaired nursing home residents. | 2020 | Aging & mental health |
| 111 | Lam, Byron L; Lee, David J; Gomez-Marin, Orlando; Zheng, D Diane; Caban, Alberto J | Concurrent visual and hearing impairment and risk of mortality: the National Health Interview Survey. | 2006 | Archives of ophthalmology |
| 112 | Lee, David J; Gomez-Marin, Orlando; Lam, Byron L; Zheng, D Diane; Arheart, Kristopher L; Christ, Sharon L; Caban, Alberto J | Severity of concurrent visual and hearing impairment and mortality: the 1986-1994 National Health Interview Survey. | 2007 | Journal of aging and health |
| 113 | Lee, David J; Lam, Byron L; Gomez-Marin, Orlando; Zheng, D Diane; Caban, Alberto J | Concurrent hearing and visual impairment and morbidity in community-residing adults: the National Health Interview Survey, 1986 to 1996. | 2005 | Journal of aging and health |
| 114 | Lehane C.M.; Dammeyer J.; Wittich W. | Intra- and interpersonal effects of coping on the psychological well-being of adults with sensory loss and their spouses | 2019 | Disability and rehabilitation |
| 115 | Lehane, Christine M; Dammeyer, Jesper; Elsass, Peter | Sensory loss and its consequences for couples' psychosocial and relational wellbeing: an integrative review. | 2017 | Aging & mental health |
| 116 | Liljas, Ann E M; Walters, Kate; de Oliveira, Cesar; Wannamethee, S Goya; Ramsay, Sheena E; Carvalho, Livia A | Self-Reported Sensory Impairments and Changes in Cognitive Performance: A Longitudinal 6-Year Follow-Up Study of English Community-Dwelling Adults Aged 50 Years. | 2018 | Journal of aging and health |
| 117 | Lin, Michael Y; Gutierrez, Peter R; Stone, Katie L; Yaffe, Kristine; Ensrud, Kristine E; Fink, Howard A; et al. | Vision impairment and combined vision and hearing impairment predict cognitive and functional decline in older women. | 2004 | Journal of the American Geriatrics Society |
| 118 | Linden-Bostrom, Margareta; Persson, Carina | Disparities in mental health among adolescents with and without impairments. | 2015 | Scandinavian journal of public health |
| 119 | Liu P.L.; Cohen H.J.; Fillenbaum G.G.; Burchett B.M.; Whitson H.E. | Association of Co-Existing Impairments in Cognition and Self-Rated Vision and Hearing With Health Outcomes in Older Adults | 2016 | Gerontology and Geriatric Medicine |
| 120 | Liu, Wenwen; Yang, Chao; Liu, Lili; Kong, Guilan; Zhang, Luxia | Bidirectional associations of vision loss, hearing loss, and dual sensory loss with depressive symptoms among the middle-aged and older adults in China. | 2022 | Journal of affective disorders |
| 121 | Loprinzi P.D.; Smit E.; Pariser G. | Association among depression, physical functioning, and hearing and vision impairment in adults with diabetes | 2013 | Diabetes Spectrum |
| 122 | Loprinzi, Paul D; Smit, Ellen; Lin, Frank R; Gilham, Ben; Ramulu, Pradeep Y | Accelerometer-assessed physical activity and objectively determined dual sensory impairment in US adults. | 2013 | Mayo Clinic proceedings |
| 123 | Lundin, Elin; Widen, Stephen E; Wahlqvist, Moa; Anderzen-Carlsson, Agneta; Granberg, Sarah | Prevalence, diagnoses and rehabilitation services related to severe dual sensory loss (DSL) in older persons: a cross-sectional study based on medical records. | 2020 | International journal of audiology |
| 124 | Luo, Yanan; He, Ping; Guo, Chao; Chen, Gong; Li, Ning; Zheng, Xiaoying | Association Between Sensory Impairment and Dementia in Older Adults: Evidence from China. | 2018 | Journal of the American Geriatrics Society |
| 125 | Lupsakko, Taina; Mantyjarvi, Maija; Kautiainen, Hannu; Sulkava, Raimo | Combined hearing and visual impairment and depression in a population aged 75 years and older. | 2002 | International journal of geriatric psychiatry |
| 126 | Lyu, Jiyoung; Kim, Hae-Young | Gender-Specific Associations of Sensory Impairments with Depression and Cognitive Impairment in Later Life. | 2018 | Psychiatry investigation |
| 127 | Ma X.; Wei J.; Congdon N.; Li Y.; Shi L.; Zhang D. | Longitudinal Association Between Self-Reported Sensory Impairments and Episodic Memory among Older Adults in China: A Prospective Cohort Study | 2021 | Journal of Geriatric Psychiatry and Neurology |
| 128 | Mactaggart, Islay; Polack, Sarah; Murthy, Gvs; Kuper, Hannah | A population-based survey of visual impairment and its correlates in Mahabubnagar district, Telangana State, India. | 2018 | Ophthalmic epidemiology |
| 129 | Mah, Ho Y; Ishak, Wan S; Abd Rahman, Mohd H | Prevalence and risk factors of dual sensory impairment among community-dwelling older adults in Selangor: A secondary data analysis. | 2020 | Geriatrics & gerontology international |
| 130 | Maharani, Asri; Dawes, Piers; Nazroo, James; Tampubolon, Gindo; Pendleton, Neil; Sense-Cog WP1 Group | Associations Between Self-Reported Sensory Impairment and Risk of Cognitive Decline and Impairment in the Health and Retirement Study Cohort. | 2020 | The journals of gerontology. Series B, Psychological sciences and social sciences |
| 131 | Maharani, Asri; Dawes, Piers; Nazroo, James; Tampubolon, Gindo; Pendleton, Neil; Sense-Cog WP1 group | Visual and hearing impairments are associated with cognitive decline in older people. | 2018 | Age and ageing |
| 132 | Marmamula, Srinivas; Kumbham, Thirupathi Reddy; Modepalli, Satya Brahmanandam; Barrenkala, Navya Rekha; Yellapragada, Ratnakar; Shidhaye, Rahul | Depression, combined visual and hearing impairment (dual sensory impairment): a hidden multi-morbidity among the elderly in Residential Care in India. | 2021 | Scientific reports |
| 133 | Maruta, Michio; Tabira, Takayuki; Sagari, Akira; Miyata, Hironori; Yoshimitsu, Koji; Han, Gwanghee; Yoshiura, Kazuhiro; Matsuo, Takashi; Kawagoe, Masahiro | Impact of sensory impairments on dementia incidence and symptoms among Japanese older adults. | 2020 | Psychogeriatrics |
| 134 | Maruta, Michio; Tabira, Takayuki; Sagari, Akira; Miyata, Hironori; Yoshimitsu, Koji; Han, Gwanghee; Yoshiura, Kazuhiro; Matsuo, Takashi; Kawagoe, Masahiro | Impact of sensory impairments on dementia incidence and symptoms among Japanese older adults. | 2019 | Psychogeriatrics |
| 135 | McDonnall, Michele Capella | Physical status as a moderator of depressive symptoms among older adults with dual sensory loss. | 2011 | Rehabilitation psychology |
| 136 | McDonnall, Michele Capella | The effects of developing a dual sensory loss on depression in older adults: a longitudinal study. | 2009 | Journal of aging and health |
| 137 | McDonnall, Michele Capella | The Effect of Productive Activities on Depressive Symptoms Among Older Adults With Dual Sensory Loss. | 2011 | Research on aging |
| 138 | Meuwese-Jongejeugd, Anneke; van Splunder, Jacques; Vink, Marianne; Stilma, Jan Sietse; van Zanten, Bert; Verschuure, Hans; Bernsen, Roos; Evenhuis, Heleen | Combined sensory impairment (deaf-blindness) in five percent of adults with intellectual disabilities. | 2008 | American journal of mental retardation |
| 139 | Michalowsky, Bernhard; Hoffmann, Wolfgang; Kostev, Karel | Association Between Hearing and Vision Impairment and Risk of Dementia: Results of a Case-Control Study Based on Secondary Data. | 2019 | Frontiers in aging neuroscience |
| 140 | Mick, Paul Thomas; Hamalainen, Anni; Kolisang, Lebo; Pichora-Fuller, M Kathleen; Phillips, Natalie; Guthrie, Dawn; Wittich, Walter | The Prevalence of Hearing, Vision, and Dual Sensory Loss in Older Canadians: An Analysis of Data from the Canadian Longitudinal Study on Aging. | 2021 | Canadian journal on aging |
| 141 | Mick, Paul; Parfyonov, Maksim; Wittich, Walter; Phillips, Natalie; Guthrie, Dawn; Kathleen Pichora-Fuller, M | Associations between sensory loss and social networks, participation, support, and loneliness: Analysis of the Canadian Longitudinal Study on Aging. | 2018 | Canadian family physician |
| 142 | Mitoku, Kazuko; Masaki, Naoko; Ogata, Yukiko; Okamoto, Kazushi | Vision and hearing impairments, cognitive impairment and mortality among long-term care recipients: a population-based cohort study. | 2016 | BMC geriatrics |
| 143 | Miyawaki, Atsushi; Kobayashi, Yasuki; Kawachi, Ichiro | Self-Reported Hearing/Visual Loss and Mortality in Middle-Aged and Older Adults: Findings From the Komo-Ise Cohort, Japan. | 2020 | Journal of epidemiology |
| 144 | Moller, Kerstin | Deafblindness: a challenge for assessment--is the ICF a useful tool?. | 2003 | International journal of audiology |
| 145 | Morandi, Alessandro; Inzitari, Marco; Udina, Cristina; Gual, Neus; Mota, Miriam; Tassistro, Elena; et al. | Visual and Hearing Impairment Are Associated With Delirium in Hospitalized Patients: Results of a Multisite Prevalence Study. | 2021 | Journal of the American Medical Directors Association |
| 146 | Mudie, Lucy I; Varadaraj, Varshini; Gajwani, Prateek; Munoz, Beatriz; Ramulu, Pradeep; Lin, Frank R; Swenor, Bonnielin K; Friedman, David S; Zebardast, Nazlee | Dual sensory impairment: The association between glaucomatous vision loss and hearing impairment and function. | 2018 | PloS one |
| 147 | Mueller-Schotte, Sigrid; Zuithoff, Nicolaas P A; van der Schouw, Yvonne T; Schuurmans, Marieke J; Bleijenberg, Nienke | Trajectories of Limitations in Instrumental Activities of Daily Living in Frail Older Adults With Vision, Hearing, or Dual Sensory Loss. | 2019 | The journals of gerontology. Series A, Biological sciences and medical sciences |
| 148 | Ogliari, Giulia; Ryg, Jesper; Qureshi, Nadeem; Andersen-Ranberg, Karen; Scheel-Hincke, Lasse Lybecker; Masud, Tahir | Subjective vision and hearing impairment and falls among community-dwelling adults: a prospective study in the Survey of Health, Ageing and Retirement in Europe (SHARE). | 2021 | European geriatric medicine |
| 149 | Olakunde, Babayemi O; Pharr, Jennifer R | HIV-related risk behaviors and HIV testing among people with sensory disabilities in the United States. | 2020 | International journal of STD & AIDS |
| 150 | Pabst, Alexander; Bar, Jonathan; Rohr, Susanne; Lobner, Margrit; Kleineidam, Luca; Heser, Kathrin; et al. | Do self-reported hearing and visual impairments predict longitudinal dementia in older adults?. | 2021 | Journal of the American Geriatrics Society |
| 151 | Parada, Humberto; Laughlin, Gail A; Yang, Mingan; Nedjat-Haiem, Frances R; McEvoy, Linda K | Dual impairments in visual and hearing acuity and age-related cognitive decline in older adults from the Rancho Bernardo Study of Healthy Aging. | 2021 | Age and ageing |
| 152 | Pardhan, Shahina; Lopez Sanchez, Guillermo F; Bourne, Rupert; Davis, Adrian; Leveziel, Nicolas; Koyanagi, Ai; Smith, Lee | Visual, hearing, and dual sensory impairment are associated with higher depression and anxiety in women. | 2021 | International journal of geriatric psychiatry |
| 153 | Pardhan, Shahina; Smith, Lee; Bourne, Rupert; Davis, Adrian; Leveziel, Nicolas; Jacob, Louis; Koyanagi, Ai; Lopez-Sanchez, Guillermo F | Combined Vision and Hearing Difficulties Results in Higher Levels of Depression and Chronic Anxiety: Data From a Large Sample of Spanish Adults. | 2020 | Frontiers in psychology |
| 154 | Petrovsky, Darina V; Sefcik, Justine S; Hanlon, Alexandra L; Lozano, Alicia J; Cacchione, Pamela Z | Social Engagement, Cognition, Depression, and Comorbidity in Nursing Home Residents With Sensory Impairment. | 2019 | Research in gerontological nursing |
| 155 | Phua, June; Visaria, Abhijit; Ostbye, Truls; Malhotra, Rahul | Association of vision and hearing impairments with quality of life among older adults: Mediation by psychosocial factors. | 2022 | Geriatrics & gerontology international |
| 156 | R, Deepthi; Kasthuri, Arvind | Visual and hearing impairment among rural elderly of south India: a community-based study. | 2012 | Geriatrics & gerontology international |
| 157 | Raina P.; Wong M.; Massfeller H. | The relationship between sensory impairment and functional independence among elderly | 2004 | BMC Geriatrics |
| 158 | Ramamurthy D, Kasthuri A, Sonavane R | Dual sensory impairment among community dwelling rural elderly: concern for rehabilitation | 2014 | J Geriatr |
| 159 | Reed, Nicholas S; Assi, Lama; Pedersen, Emily; Alshabasy, Yasmeen; Deemer, Ashley; Deal, Jennifer A; Willink, Amber; Swenor, Bonnielin K | Accompaniment to healthcare visits: the impact of sensory impairment. | 2020 | BMC health services research |
| 160 | Reuben, D B; Mui, S; Damesyn, M; Moore, A A; Greendale, G A | The prognostic value of sensory impairment in older persons. | 1999 | Journal of the American Geriatrics Society |
| 161 | Roets-Merken, Lieve; Zuidema, Sytse; Vernooij-Dassen, Myrra; Dees, Marianne; Hermsen, Pieter; Kempen, Gertrudis; Graff, Maud | Problems identified by dual sensory impaired older adults in long-term care when using a self-management program: A qualitative study. | 2017 | PloS one |
| 162 | Rong, Hongguo; Lai, Xiaozhen; Jing, Rize; Wang, Xiao; Fang, Hai; Mahmoudi, Elham | Association of Sensory Impairments With Cognitive Decline and Depression Among Older Adults in China. | 2020 | JAMA network open |
| 163 | Schneck, Marilyn E; Lott, Lori A; Haegerstrom-Portnoy, Gunilla; Brabyn, John A | Association between hearing and vision impairments in older adults. | 2012 | Ophthalmic & physiological optics |
| 164 | Schneider, Julie M; Gopinath, Bamini; McMahon, Catherine M; Leeder, Stephen R; Mitchell, Paul; Wang, Jie Jin | Dual sensory impairment in older age. | 2011 | Journal of aging and health |
| 165 | Schneider, Julie; Gopinath, Bamini; McMahon, Catherine; Teber, Erdahl; Leeder, Stephen R; Wang, Jie Jin; Mitchell, Paul | Prevalence and 5-year incidence of dual sensory impairment in an older Australian population. | 2012 | Annals of epidemiology |
| 166 | Shakarchi, Ahmed F; Assi, Lama; Ehrlich, Joshua R; Deal, Jennifer A; Reed, Nicholas S; Swenor, Bonnielin K | Dual Sensory Impairment and Perceived Everyday Discrimination in the United States. | 2020 | JAMA ophthalmology |
| 167 | Shakarchi, Ahmed F; Assi, Lama; Gami, Abhishek; Kohn, Christina; Ehrlich, Joshua R; Swenor, Bonnielin K; Reed, Nicholas S | The Association of Vision, Hearing, and Dual-Sensory Loss with Walking Speed and Incident Slow Walking: Longitudinal and Time to Event Analyses in the Health and Retirement Study. | 2021 | Seminars in hearing |
| 168 | Simcock, Peter | Ageing with a unique impairment: a systematically conducted review of older deafblind people's experiences | 2017 | Ageing & Society |
| 169 | Simcock, Peter | One of society's most vulnerable groups? A systematically conducted literature review exploring the vulnerability of deafblind people. | 2017 | Health & social care in the community |
| 170 | Simning, Adam; Fox, Meghan L; Barnett, Steven L; Sorensen, Silvia; Conwell, Yeates | Depressive and Anxiety Symptoms in Older Adults With Auditory, Vision, and Dual Sensory Impairment. | 2019 | Journal of aging and health |
| 171 | Smith, Sherri L; Bennett, Loren W; Wilson, Richard H | Prevalence and characteristics of dual sensory impairment (hearing and vision) in a veteran population. | 2008 | Journal of rehabilitation research and development |
| 172 | Soto-Perez-de-Celis, Enrique; Sun, Can-Lan; Tew, William P; Mohile, Supriya Gupta; Gajra, Ajeet; et al. | Association between patient-reported hearing and visual impairments and functional, psychological, and cognitive status among older adults with cancer. | 2018 | Cancer |
| 173 | Steinman, Bernard A; Tabler, Jennifer; Mittlieder, Casandra M; Whitlock, Bremen; Goodman, Carrie E | Self-Reported Sensory Impairments in Older Adults and their Association with Self-Rated Health and Mortality Outcomes. | 2021 | Journal of aging and health |
| 174 | Sun, Ji; Li, Lin; Sun, Jiangwei | Sensory impairment and all-cause mortality among the elderly adults in China: a population-based cohort study. | 2020 | Aging |
| 175 | Swenor, Bonnielin K; Ramulu, Pradeep Y; Willis, Jeffery R; Friedman, David; Lin, Frank R | The prevalence of concurrent hearing and vision impairment in the United States | 2013 | JAMA internal medicine |
| 176 | Tan, Benjamin Kye Jyn; Ng, Faye Yu Ci; Song, Harris Jun Jie Muhammad Danial; Tan, Nicole Kye Wen; Ng, Li Shia; Loh, Woei Shyang | Associations of Hearing Loss and Dual Sensory Loss With Mortality: A Systematic Review, Meta-analysis, and Meta-regression of 26 Observational Studies With 1213756 Participants. | 2022 | JAMA otolaryngology |
| 177 | Tareque, Md Ismail; Chan, Angelique; Saito, Yasuhiko; Ma, Stefan; Malhotra, Rahul | The Impact of Self-Reported Vision and Hearing Impairment on Health Expectancy. | 2019 | Journal of the American Geriatrics Society |
| 178 | Teh ChuAi [Teh, C. A. R.]; Lim WeeShiong; Basri, R.; Ismail, N. H. | Utility of a patient-response screening question for visual impairment. | 2006 | Journal of the American Geriatrics Society |
| 179 | Tinetti, M E; Inouye, S K; Gill, T M; Doucette, J T | Shared risk factors for falls, incontinence, and functional dependence. Unifying the approach to geriatric syndromes. | 1995 | JAMA |
| 180 | Tseng, Ya-Chuan; Liu, Sara Hsin-Yi; Lou, Meei-Fang; Huang, Guey-Shiun | Quality of life in older adults with sensory impairments: a systematic review. | 2018 | Quality of life research |
| 181 | Turunen-Taheri, Satu; Skagerstrand, Asa; Hellstrom, Sten; Carlsson, Per-Inge | Patients with severe-to-profound hearing impairment and simultaneous severe vision impairment: a quality-of-life study. | 2017 | Acta oto-laryngologica |
| 182 | Urqueta Alfaro, Andrea; Guthrie, Dawn M; McGraw, Cathy; Wittich, Walter | Older adults with dual sensory loss in rehabilitation show high functioning and may fare better than those with single sensory loss. | 2020 | PloS one |
| 183 | Viljanen, A.; Kulmala, J.; Rantakokko, M.; Koskenvuo, M.; Kaprio, J.; Rantanen, T. | Accumulation of sensory difficulties predicts fear of falling in older women. | 2013 | Journal of Aging and Health |
| 184 | Viljanen, Anne; Tormakangas, Timo; Vestergaard, Sonja; Andersen-Ranberg, Karen | Dual sensory loss and social participation in older Europeans. | 2014 | European journal of ageing |
| 185 | Vreeken, Hilde L; van Rens, Ger H M B; Knol, Dirk L; van Reijen, Nadja A; Kramer, Sophia E; Festen, Joost M; van Nispen, Ruth M A | Dual sensory loss: A major age-related increase of comorbid hearing loss and hearing aid ownership in visually impaired adults. | 2014 | Geriatrics & gerontology international |
| 186 | Wahl, Hans-Werner; Heyl, Vera; Drapaniotis, Philipp M; Hormann, Karl; Jonas, Jost B; Plinkert, Peter K; Rohrschneider, Klaus | Severe vision and hearing impairment and successful aging: a multidimensional view. | 2013 | The Gerontologist |
| 187 | Williams, Nicole; Phillips, Natalie A; Wittich, Walter; Campos, Jennifer L; Mick, Paul; Orange, Joseph B; et al. | Hearing and Cognitive Impairments Increase the Risk of Long-term Care Admissions. | 2020 | Innovation in aging |
| 188 | Wittich, Walter; Watanabe, Donald H; Gagne, Jean-Pierre | Sensory and demographic characteristics of deafblindness rehabilitation clients in Montreal, Canada. | 2012 | Ophthalmic & physiological optics |
| 189 | Xie, Tian; Liu, Danxia; Guo, Jing; Zhang, Bo | The longitudinal effect of sensory loss on depression among Chinese older adults. | 2021 | Journal of affective disorders |
| 190 | Yamada, Yukari; Denkinger, Michael D; Onder, Graziano; Finne-Soveri, Harriet; van der Roest, Henriette; Vlachova, Martina; Richter, Tomas; Gindin, Jacob; Bernabei, Roberto; Topinkova, Eva | Impact of dual sensory impairment on onset of behavioral symptoms in European nursing homes: results from the Services and Health for Elderly in Long-Term Care study. | 2015 | Journal of the American Medical Directors Association |
| 191 | Yamada, Yukari; Denkinger, Michael D; Onder, Graziano; Henrard, Jean-Claude; van der Roest, Henriette G; Finne-Soveri, Harriet; Richter, Tomas; Vlachova, Martina; Bernabei, Roberto; Topinkova, Eva | Dual Sensory Impairment and Cognitive Decline: The Results From the Shelter Study. | 2016 | The journals of gerontology. Series A, Biological sciences and medical sciences |
| 192 | Yamada, Yukari; Denkinger, Michael D; Onder, Graziano; van der Roest, Henriette G; Finne-Soveri, Harriet; Bernabei, Roberto; Topinkova, Eva | Joint Associations of Dual Sensory Impairment and No-Activity Involvement With 1-Year Mortality in Nursing Homes: Results From the SHELTER Study. | 2016 | The journals of gerontology. Series A, Biological sciences and medical sciences |
| 193 | Yamada, Yukari; Vlachova, Martina; Richter, Tomas; Finne-Soveri, Harriet; Gindin, Jacob; van der Roest, Henriette; et al. | Prevalence and correlates of hearing and visual impairments in European nursing homes: results from the SHELTER study. | 2014 | Journal of the American Medical Directors Association |
| 194 | Yorgason J.B.; Tanner C.T.; Richardson S.; Hill M.M.Y.S.; Stagg B.; Wettstein M.; Ehrlich J.R. | The Longitudinal Association of Late-Life Visual and Hearing Difficulty and Cognitive Function: The Role of Social Isolation | 2022 | Journal of aging and health |
| 195 | Zhang, Y; Ge, M; Zhao, W; Liu, Y; Xia, X; Hou, L; Dong, B | Sensory Impairment and All-Cause Mortality Among the Oldest-Old: Findings from the Chinese Longitudinal Healthy Longevity Survey (CLHLS). | 2020 | The journal of nutrition, health & aging |
| 196 | Zhao, Xiaohuan; Zhou, Yifan; Wei, Kunchen; Bai, Xinyue; Zhang, Jingfa; Zhou, Minwen; Sun, Xiaodong | Associations of sensory impairment and cognitive function in middle-aged and older Chinese population: The China Health and Retirement Longitudinal Study. | 2021 | Journal of global health |
| 197 | Zhou Y.; Hu Y.; Luo J.; Li Y.; Liu H.; Sun X.; Zhou M. | Association Between Sensory Loss and Falls Among Middle-Aged and Older Chinese Population: Cross-Sectional and Longitudinal Analyses | 2022 | Frontiers in Medicine |
