# Supplementary material for: Prevalence and impact of combined vision and hearing (dual sensory) impairment: A scoping review
Source: PLOS Glob Public Health. 2023 May 16;3(5):e0001905. doi: 10.1371/journal.pgph.0001905 (PMC10187940; doi:10.1371/journal.pgph.0001905)
Supplement: Attachment — Submitted filename: Response to reviewers.docx [file pgph.0001905.s009.docx]

**REVIEWER 1**

**Prevalence and impact of combined vision and hearing (dual sensory) impairment: a scoping review**

Thank you for the opportunity to review this article. This review is important for the field of dual sensory impairment given the increasing aging population and the impacts of the sensory losses to daily life. A strong scoping review methodology was utilized, and a thorough analysis has been complete. A great figure has been developed to showcase the prevalence reported per each article by country. There are a few recommendations that have been highlighted below to ensure better clarity for readers and ensuring consistency throughout the article. Given the significance of this paper and the strong work completed by the team, revision of the manuscript with the below items would help improve the quality of the review and be significant to the dual sensory impairment field. Specific suggestions based on each main section of the paper are listed below.

We appreciate your positive comments.

**Abstract**

1. Overall, the abstract is well-structured and is easily understandable.

Thank you.

2. Line 43: The abstract indicates there are 183 reports of 153 unique primary studies and 14 review articles. In the PRISMA diagram, it indicates there are a total of 197 reports. Please double check the numbers.

Apologies for the confusion, 197=183+14. This has been clarified in the diagram.

**Introduction**

1. The World Federation of the DeafBlind report is an important global report on the experiences of individuals with deafblindness, highlighting key findings from LMICs, surveys, and academic articles. Please incorporate and cite key findings from this report into your introduction, where appropriate.

Thank you for highlighting the importance of this article. We have now referenced this in the introduction.

2. In lines 59, please include combined/concurrent vision and hearing impairment, as another term for DSI.

This has been addressed

3. Line 66: Incorporate the terms congenital, acquired, and age-related deafblindness when describing the different types (i.e., congenital deafblindness=people who have DSI from birth).

Thank you for the suggestion. This has been amended.

**Methods**

1. A strong methodology was employed, and an information specialist created/ran the search strategy, along with using PRISMA-ScR.

Thankyou

2. Eligibility Criteria: Suggest using a table for the inclusion/exclusion criteria for better organization.

Thank you for your suggestion, we prefer not to include another table in the paper.

3. I recommend using the updated 2020 PRISMA flowchart. This can be [found here](https://prisma-statement.org/prismastatement/flowdiagram.aspx). Please cite the flowchart too if utilized.

Thank you for the suggestion, we prefer the PRISMA flow chart that we have adapted for this paper as we did not search registers. We have added a reference.

4. In table 1, you have categorized older adults as (1) adults aged 40 and older and (2) adults aged 70 and older. It is unclear what age group is considered an older adult in this review. [The WHO](https://www.who.int/health-topics/ageing#tab=tab_1) has categorized individuals aged 60 and older as “older adults.” Can you include a sentence for the definition of older adults, including a rational for this decision or reference to other articles/organizations? One sentence will be sufficient.

Thank you for this feedback. As our report highlights, the age categories across included studies were extremely heterogeneous. We chose our categories after an initial review of the included studies, trying to show some differentiation. We have added to the discussion as you suggested that future studies should align with the WHO classifications.

5. A recommendation is to not include reviews in this paper and instead snowball them to ensure that the relevant articles have been picked up by your search. Given your paper conducted a search from inception to 2022, your search on databases/snowballing should have picked up these articles and will prevent duplication of the same articles. If you feel that including these reviews is important for your review, please include a rationale for this decision in the methodology section.

Thank you for this suggestion. We have now added a sentence to the methods section:

*We included systematic reviews to understand which aspects of DSI have received the most attention by researchers synthesizing the evidence.*

**Results**

1. Overall, it is unclear where the number 153 studies were obtained from given the PRISMA diagram says 183 primary studies and 14 reviews. For example, In the PRISMA flowchart, it says there are 187 primary studies and 14 reviews, but in line 178 it says 183 reported outcomes of 153 unique primary studies. This is consistent throughout the paper, please clarify and fix this throughout the paper.

In line 180, we state “Of the included reports, 14 were reviews and 183 reported outcomes of 153 unique primary studies”, i.e. the 183 primary studies were from 153 unique studies – i.e. multiple publications per study. This has been clarified in the text: “Of the included reports, 14 were reviews and the 183 primary reports came from 153 unique primary studies (i.e. multiple publications per study).”

2. Cite any studies you are discussing in the results section. For instance, in line 225, cite the study that you are discussing about. This comment applies for different section in the results section.

Citations have been added throughout the results section.

3. See comment #4 under methods section – The findings that are reported in line 216-220 can be included in the discussion section instead as this will likely be a similar finding you identified from your review.

We feel that this is appropriate for the results section, and have therefore left it as it is.

4. Based on the inclusion criteria, I do not see a relevant review (*Prevalence of Individuals With Deafblindness and Age-Related Dual-Sensory Loss*) included, that was published in Jan 2022. I recommend conducting a search on deafblindness Journals (*Journal of Visual Impairment and Blindness’ and the ‘British Journal of Visual Impairment’*) to see if there any relevant articles that are being missed that can be included in this review.

Thank you for highlighting this review article and thank you for the suggestion of searching the ‘Journal of Visual Impairment and Blindness’ and the ‘British Journal of Visual Impairment’. These two journals should have been already included in our searches as they are indexed on Medline. As the review article was published in January 2022 it should have been picked up in our searches in April 2022 but was not – it is likely that the article was not indexed by the date of our searches in April.

We have added the following text to the limitations section. “Fourth, we only conducted electronic searches and did not handsearch journals. It is possible that we may have missed articles that were not properly indexed on the electronic databases, or where indexing was delayed  – for example, a potentially relevant review on the prevalence of DSI (ref) published in January 2022 was not indexed by the time of our searches in April 2022. However, the findings are consistent with the findings of our scoping review – that prevalence increases by age, and the findings across studies were often not comparable”

5. In line 317, to make it clear that you are only looking at articles that reported ADL, I suggest changing it to: In studies reporting ADL, people with DSI had worse….. Likewise, in studies reporting social participation, 75% of the studies reported worse social participation.

Thank you for the suggestion, this has been addressed.

**Discussion**

1. Under the *Prevalence of DSI* section, it might be relevant to discuss what the issues regarding different age group can mean in interpreting prevalence rates, as well as an area of recommendation for future researchers to stick to specific age categories recommended by WHO (see comment 4 under introduction).

Thank you for the suggestion. We have added a sentence: “The age included in the prevalence estimates has implications for service planning and delivery, which will be different for children, working age adults, and the elderly. Future studies should align age categories with those recommended by WHO.”

2. As well, under this section, it will be important to highlight any differences in prevalence rate between different countries and the reasons for the difference, if any. For instance, this can relate to social services/systems available in these countries compared to others.

Thank you for your comment, we have added a sentence to the discussion: . The majority of the literature came from high-income countries, so any differences in prevalence rate between low- and high-income countries could not be examined in depth. This warrants further investigation, as the prevalence may be higher in LMICs due to factors such as poor access to services.

**Limitations**

1. This is a good limitation section. No other suggestions.

Thank you for the feedback.

**Conclusion**

1. This is a good conclusion. It can be strengthened by including a concluding sentence about prevalence, such as in Line 408.

Thank you for your suggestion. We feel that a concluding sentence about prevalence has already been included and therefore have not added anything further.

2. As well, answering questions like, what are some key implications for policy, practice and future research? Who needs to act on this information, and how can policies, organizations, and governments do to help make the required changes?

We have added a sentence to summarise the points raised about future research throughout the discussion and an overall sentence mentioning policy and practice. As this is a scoping review, it does not allow formulation of policy recommendations which are best developed for specific contexts by appropriately constituted guideline committees:

*Further there is an urgent need for research to identify the most effective strategies to improve access to health and wellbeing services for people with DSI. These findings are important for policy and practice when trying to address the additional needs of people experiencing DSI, beyond the impairments alone.*

3. Lastly, the conclusion will be a good opportunity to provide recommendations to future research/policymakers.

Thank you. We believe we addressed this in our response to the point directly above.

**REVIEWER 2**

Reviewer #2: Thank you for giving me the opportunity to review this excellent scoping review. I need to declare right at the beginning that this manuscript covers a topic that the field of deafblindness has been waiting for, and its content will likely make it a highly cited contribution to the literature. I am most excited that this work has been done and I congratulate the authors on their quality.

Thank you for the positive feedback.

Having said that, there are a few minor items that I believe are important to be incorporated, to give the work a touch more subtlety and context. I provide these here in the spirit of elevating an already excellent manuscript even further :

Thank you for your suggestions, we have addressed each of these in turn

The phrase “there is currently no consensus on how to define DSI,…” is not entirely correct, given that the Nordic definition of deafblindness is widely used and accepted in the field of deafblindness rehabilitation and service delivery: Centre for Welfare and Social Issues. (2016). The Nordic definition of deafblindness. <http://www.kuurosokeat.fi/tiedosto/nordic_definition.pdf>. In addition, there are definitions that follow the medical model in the context of eligibility criteria for services (e.g., the authors cite Wittich et al. 2012 which contains an eligibility grid for service access). It is however true that there is disagreement between whether a standard definition should rely on behaviorally measurable observations (e.g., acuity, field, audiogram) or functional impairment (subjectively reported). This disagreement is not well represented in the introduction at present.

Thank you for your comment. The introduction has been amended to reflect your feedback.

*There are internationally agreed World Health Organization (WHO) definitions of hearing and vision impairment separately, and some agreement on within the deafblindness field through the Nordic definition of deafblindness for rehabilitation and service delivery. However, there is currently no consensus on how to measure and define DSI, particularly in terms of the severity of the underlying impairments when they co-exist, and whether a definition should rely on behaviousally measurable observations (e.g. acuity, audiogram) or functional impairment (subjectively reported).*

As the authors mention, the variability in all aspects is remarkable, and problematic. I believe that the authors should use this variability to justify at the beginning of the methods section why a scoping review is indeed the correct choice of methodology, over other approaches to reviewing the literature.

Thank you for your feedback. We have added the following to justify the reason why we did a scoping review. This came from our review protocol.

*As this study only included published data, ethics approval was not sought. We decided to undertake a scoping review rather than a systematic review in order to identify and map the available evidence on these themes, and expected the results to be heterogenous.*

Given the variability and sparsity of information about deafblindness prevalence, many previous studies have had to rely on government sources for data on the topic. Methodologically here, it is solid to rely on the peer-reviewed literature, but given the history and young age of the field of deafblindness, I suggest that the authors include a short comparison of their findings with some of the sources that are widely known to researchers in deafblindness, such as :

- European Deafblind Network. (2014). European Deafblind Indicators: Mapping opportunities for deafblind people across Europe.
  United Nations Department of Economic and Social Affairs Population Division. (2022). World Population Prospects 2022: Summary of Results - DESA/POP/2022/TR/NO. 3. Author. <https://www.un.org/development/desa/pd/sites/www.un.org.development.desa.pd/files/wpp2022_summary_of_results.pdf>
- Robertson, J., & Emerson, E. (2010). Estimating the number of people with co-occurring vision and hearing impairments in the UK.
  World Federation of the Deafblind. (2018). At Risk of Exclusion from CRPD and SDGs Implementation: Inequality and Persons with Deafblindness. Initial Global Report 2018. <https://wfdb.eu/wfdb-report-2018/>

We have added a comparison to these findings to the World Federation of the Deafblind the discussion. Including the following statements :

Under impact of DSI

*The findings of grey literature sources such as the 2018 report by the World Federation of the Deafblind also agree with the findings of our review – people with deafblindness may have poorer levels of health, and poorer levels of participation in work and education.*

Under prevalence of DSI

*The World Federation for the Deafblind report, which analysed 22 population-based surveys conducted in LMICs that measured DSI using self-report, found a pooled prevalence of 0.2%. Although our review only included 3 all age population-based studies, the prevalence in these ranged between 0.003% and 1.91% consistent with the low pooled prevalence found in the report.*

The authors need to acknowledge somewhere that individuals cannot be simply categorized into deafblind or not deafblind. Part of the confusion about criteria is that this condition comes on a sliding scale and the interaction between the two impairment creates complexity that needs to be accommodated across a spectrum.

The reviewer raises a good point. We have added this point to the discussion section :

*Another challenge with definitions of DSI, is that individuals cannot be easily categorised in to deafblind or not deafblind – the interaction between the impairments creates complexity that needs to be accommodated across a spectrum.*

Line 298 “…but there was no evidence that this was correlated with DSI itself.” Do you mean correlated with level of severity or with the presence of DSI – here is one of those moments where that subtly of the spectrum may matter in the interpretation.

This has been clarified in the text:

*One review found that those with Usher’s syndrome, a major syndromic cause of DSI, were more at risk of developing psychological disorders, but there was no evidence that this was correlated with the presence of DSI*

If the authors want to incorporate possibilities for improvement in the future, one example is the presentation of the data that are by the Global Burden of Disease team on rehabilitation:

- Cieza, A., Causey, K., Kamenov, K., Hanson, S. W., Chatterji, S., Vos, T., Bill, F., & Foundation, M. G. (2020). Global estimates of the need for rehabilitation based on the Global Burden of Disease study 2019 : a systematic analysis for the Global Burden of Disease Study 2019. The Lancet, 6736(20), 1–12. <https://doi.org/10.1016/S0140-6736(20)32340-0>

Thank you for this suggestion, we have added the reference to the discussion and a statement to go alongside this.

*Future studies should align age categories with those recommended by WHO (e.g., older adults are aged 60 years and older), and present data in a clear and standardised format*

Both vision and hearing are reported, but not their overlap. This needs to be addressed in the future, and the current review should hint at this gap in the methodology.

Thank you for your feedback. We have suggested that future population based surveys of single impairments should consider the possible substantial overlap. In the discussion, we have added :

*Further, population-based surveys of single impairments (vision or hearing) should consider the potential overlap between the two conditions.*
